# The nature of complex structural variations in tomatoes

**DOI:** 10.1093/hr/uhaf107

**Published:** 2025-04-16

**Authors:** Xue Cui, Yuxin Liu, Miao Sun, Qiyue Zhao, Yicheng Huang, Jianwei Zhang, Qiulin Yao, Hang Yin, Huixin Zhang, Fulei Mo, Hongbin Zhong, Yang Liu, Xiuling Chen, Yao Zhang, Jiayin Liu, Youwen Qiu, Mingfang Feng, Xu Chen, Hossein Ghanizadeh, Yao Zhou, Aoxue Wang

**Affiliations:** College of Horticulture and Landscape Architecture, Northeast Agricultural University, Harbin 150030, China; College of Horticulture and Landscape Architecture, Northeast Agricultural University, Harbin 150030, China; State Key Laboratory of Forage Breeding-by-Design and Utilization, Key Laboratory of Plant Molecular Physiology, Institute of Botany, Chinese Academy of Sciences, Beijing 100093, China; College of Horticulture and Landscape Architecture, Northeast Agricultural University, Harbin 150030, China; National Key Laboratory of Crop Genetic Improvement, Huazhong Agricultural University, Wuhan 430070, China; Shenzhen Branch, Guangdong Laboratory for Lingnan Modern Agriculture, Shenzhen Key Laboratory of Agricultural Synthetic Biology, Genome Analysis Laboratory of the Ministry of Agriculture and Rural Affairs, Agricultural Genomics Institute at Shenzhen, Chinese Academy of Agricultural Sciences, Shenzhen 518124, China; National Key Laboratory of Crop Genetic Improvement, Huazhong Agricultural University, Wuhan 430070, China; Wuhan Jianbing Technology Co., Ltd., Wuhan, China; State Key Laboratory of Forage Breeding-by-Design and Utilization, Key Laboratory of Plant Molecular Physiology, Institute of Botany, Chinese Academy of Sciences, Beijing 100093, China; College of Life Sciences, Northeast Agricultural University, Harbin 150030, China; College of Life Sciences, Northeast Agricultural University, Harbin 150030, China; Shenzhen CEM Biomedical Technology Ltd., Shenzhen, China; College of Horticulture and Landscape Architecture, Northeast Agricultural University, Harbin 150030, China; College of Horticulture and Landscape Architecture, Northeast Agricultural University, Harbin 150030, China; College of Life Sciences, Northeast Agricultural University, Harbin 150030, China; College of Horticulture and Landscape Architecture, Northeast Agricultural University, Harbin 150030, China; College of Life Sciences, Northeast Agricultural University, Harbin 150030, China; College of Life Sciences, Northeast Agricultural University, Harbin 150030, China; College of Horticulture and Landscape Architecture, Northeast Agricultural University, Harbin 150030, China; College of Horticulture and Landscape Architecture, Northeast Agricultural University, Harbin 150030, China; State Key Laboratory of Forage Breeding-by-Design and Utilization, Key Laboratory of Plant Molecular Physiology, Institute of Botany, Chinese Academy of Sciences, Beijing 100093, China; Academician Workstation of Agricultural High-tech Industrial Area of the Yellow River Delta, National Center of Technology Innovation for Comprehensive Utilization of Saline-Alkali Land, Dongying 257300, China; College of Horticulture and Landscape Architecture, Northeast Agricultural University, Harbin 150030, China

## Abstract

Structural variations (SVs) in repetitive sequences could only be detected within a broad region due to imprecise breakpoints, leading to classification errors and inaccurate trait analysis. Through manual inspection at 4532 variant regions identified by integrating 14 detection pipelines between two tomato genomes, we generated an SV benchmark at base-pair resolution. Evaluation of all pipelines yielded F1-scores below 53.77% with this benchmark, underscoring the urgent need for advanced detection algorithms in plant genomics. Analyzing the alignment features of the repetitive sequences in each region, we summarized four patterns of SV breakpoints and revealed that deviations in breakpoint identification were primarily due to copy misalignment. According to the similarities among copies, we identified 1635 *bona fide* SVs with precise breakpoints, including substitutions (223), which should be taken as a fundamental SV type, alongside insertions (780), deletions (619), and inversions (13), all showing preferences for SV occurrence within AT-repeat regions of regulatory loci. This precise resolution of complex SVs will foster genome analysis and crop improvement.

## Introduction

Structural variations (SVs) are genomic alterations that can significantly influence phenotype by disrupting gene function or expression through alterations in regulatory elements or the three-dimensional (3D) structure [1, 2]. In plants, SVs play a significant role in responses to environmental stimuli and have a substantial impact on both yield and quality [3–5]. Therefore, given the importance of SVs, it is essential to deepen our understanding of their structure, prevalence, and the mechanisms by which they were involved in biological processes [6].

Currently, long-read-based algorithms for detecting SVs in genomes, including both *de novo* assembly and alignment-based methods, utilize diverse strategies such as identifying divergent alignments, junction-spanning sequences, clipped reads, and sequence clustering within specific genomic regions [7–10]. However, these methods may introduce biases due to the polymorphic, chimeric, and heterogeneous nature of sequences surrounding breakpoints, complicating the integration of SVs across multiple samples [11–14]. Consequently, attaining base-pair resolution for every SV remains challenging, thereby limiting our understanding of underlying biases [15].

Classifying SV types, such as deletions, duplications, insertions, inversions, and translocations, is theoretically straightforward; however, their practical categorization remains challenging [16]. For instance, distinguishing between duplications and insertions is often difficult due to potential alignment errors inherent in detection methodologies [17]. Additionally, the presence of complex genomic regions, such as homopolymers, segmental duplications, tandem repeats, and satellite DNA, further complicates this process [18, 19]. Therefore, treating SVs as single events in these regions makes interpreting the full scope of genetic information difficult, as the practical alignment results often obscure the repetitive sequence features [20]. This challenge is particularly evident in plant genomes, where repetitive sequences make up 35% to 90% of the genome, with polyploid species exhibiting highly repetitive regions that further complicate SV identification [21, 22]. Moreover, current variant benchmark sets exclude many of these regions, causing the performance of detection algorithms to be generally overestimated, highlighting the critical need for a more comprehensive and standardized benchmark to evaluate variant detection methods accurately.

In this study, we manually characterize complex SVs at base-pair resolution between two tomato genomes to shed light on the nature of SVs across boundaries, breakpoints, and types. This research aims to establish an SV benchmark for plant genomes and enhance our understanding of repetitive sequences involved in complex SVs.

**Figure 1:**
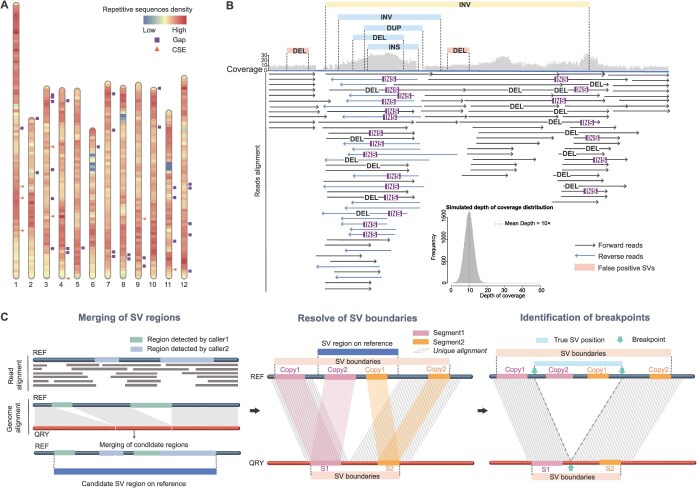
Challenges in the identification of complex SVs. A The distribution of repetitive sequences, gaps, and CSEs in the assembled genome. Repetitive sequences encompass tandem repeats and transposons. Gaps on the chromosome are indicated by squares, while CSEs are represented by triangles. B Inaccurate and false-positive SVs were identified in repetitive regions. The SVs detected by the callers, which conflicted with read alignments during visualization, revealed three distinct scenarios: (1) cases where no actual variants were present, yet deletions (DEL) were incorrectly identified (highlighted as false positive SVs); (2) regions where the entire area was misclassified as a single inversion (INV); and (3) regions where multiple types of SVs—such as INV, duplication (DUP), DEL and insertion (INS)—were detected by different callers, leading to conflicting interpretations. This figure is a schematic summary based on visual observations, with the histogram in the bottom-right corner illustrating the simulated distribution of sequencing coverage (mean depth is 10×). C The strategy for resolving complex SV identification. Firstly, SV loci identified by callers in similar regions using two methods were combined into a single SV region. Subsequently, manual analysis of repetitive sequence features through genome alignment helped determine SV boundaries and breakpoints in both genomes, facilitating the classification of SV types.

## Results

### Assembly of a high-quality tomato genome

Achieving a comprehensive understanding of complex SVs necessitates high-quality genome assemblies. By manually integrating results from three *de novo* assembly algorithms, we generated a high-quality genome for the cultivated tomato VF36, with a quality value (QV) of 63.895 (see Materials and methods, Supplementary Table S1 and Supplementary Fig. S1). The assembly consisted of 44 contigs, totaling approximately 798.8 Mb, with a contig N50 size of 43.36 Mb, which is about seven times larger than that of the reference genome Heinz 1706 [23] (SL4.0 contig N50 5.5 Mb) (Supplementary Table S2). Notably, the assembly contains only 32 gaps, primarily in regions rich in repetitive sequences (Fig. 1A), indicating high contiguity. We further validated the assembly's accuracy using the CRAQ method [24], based on re-mapping long and short reads (Supplementary Fig. S2). A genome-wide assessment revealed only eight large-scale clip-based structural errors (CSE) (S-AQI score of 99.00), distributed across chromosomes Chr01 (3), Chr03 (2), Chr04 (1), Chr09 (1), and Chr11 (1) (Fig. 1A, Supplementary Tables S3 and S4 and Supplementary Figs S3–S8). Overall, these results suggested that our assembly provided a robust foundation for detecting and analyzing SVs in tomatoes (Supplementary Fig. S9).

**Figure 2:**
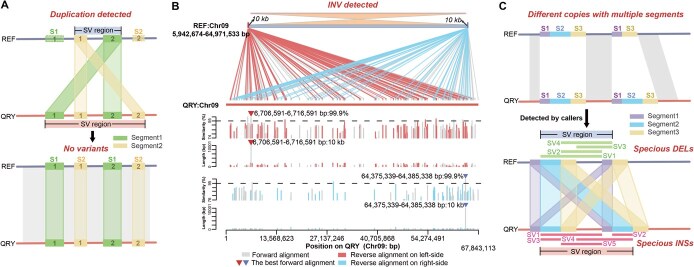
Complexity in determining SV boundaries. A The issue of copy number discrepancies. The segments of S1 and S2 are present as two copies, each in both the reference (REF) and query (QRY) genomes. However, all copies in the query genome align exclusively to a single copy in the reference genome, leading to false-positive SVs detected by callers. B Misdetection of SVs due to reverse-aligned sequences across the genome. The sequences at the start (10 kb; rhomboids on left-side) and end (10 kb; rhomboids on right-side) of an SV region from 5.94 to 64.97 Mb on Chr09 exhibited widespread alignment across the entire Chr09 in the query genome. Numerous segments on both sides aligned in reverse orientation, leading to erroneous detection as an inversion (INV). However, the best alignments for these two 10 kb sequences are in the forward orientation (triangles). C Misalignment of repetitive sequences. The SV region contains multiple copies with segments of S1, S2, and S3 in both genomes. The absence of one S2 copy in the reference genome results in incorrect alignments of these copies, leading to the erroneous detection of insertions (INSs) and deletions (DELs).

### Overview of complex SVs

In the absence of an algorithm capable of precisely localizing all SVs, we employed a total of 14 algorithms for SV detection between VF36 and SL4.0 (see Materials and methods and Supplementary Fig. S10), and identified 32 128 redundant SV loci (Supplementary Tables S5-S6). Following initial length and quality filtering, each SV locus was visually inspected using Samplot [25] and IGV [26], and manually consolidated into 4532 SV regions (see Materials and methods and Supplementary Table S7). The results showed consistent SV detection across non-repetitive regions by various callers, but detection varied significantly in repetitive regions (Fig. 1B). In these repetitive areas, the SV positions identified by the software often conflicted with those observed in the read alignments (Supplementary Figs S11–S13). Additionally, in highly repetitive regions where multiple SVs clustered together, different callers interpreted these clusters as single SVs or detected only parts of the variant sequence, leading to mixed reports of insertions, deletions, duplications, or inversions (Supplementary Figs S14 and S15).

Upon visualizing all SV loci, we identified three primary challenges in detecting complex SVs: inaccuracies in pinpointing SV regions, difficulty in determining precise start and end positions at base-pair resolution, and inconsistencies in defining different SV types. To address these issues, we performed an analysis focusing on SV boundaries, breakpoints, and types in relation to genome sequence features (Fig. 1C).

### Determination of SV boundaries

A key challenge in accurately identifying SVs is determining their precise boundaries. Through manual inspection of 4,532 SV regions based on the alignment of two genomes, we identified three recurring scenarios that underscore the complexities in defining these boundaries (Supplementary Figs. S16–S17). 

Firstly, there was an issue with copy number discrepancies (2026/4532), where multiple copies were present in both the reference and query genomes, but all copies in the query genome aligned to the same position in the reference genome, falsely indicating a deletion or insertion (Fig. 2A and Supplementary Fig. S22). Secondly, SVs could be misidentified due to sequences aligning in reverse orientation across the genome, leading to falsely detected large-scale SVs (87/4532) (Fig. 2B and Supplementary Fig. S23). For instance, a single SV misreported by Sniffles [11] spanned a large portion of a chromosome (5.94 to 64.97 Mb, covering 86.16% of Chr09). The third scenario involved the misalignment of repetitive sequences (2129/4532), where different copies were incorrectly paired, causing boundary shifts and erroneous SV calls (Fig. 2C and Supplementary Fig. S24). Additionally, 290 SV regions were located in highly complex genomic areas, displaying combinations of these issues.

**Figure 3:**
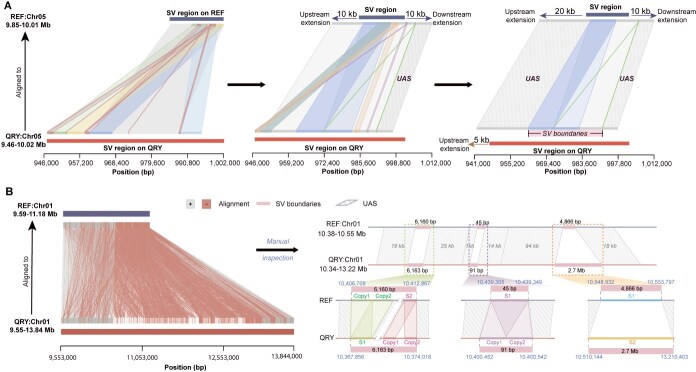
Approach for determining SV boundaries. A The pipelines for determining boundaries. For an SV region located on Chr05, the alignment of two genomes revealed multiple repetitive segments. SV boundaries were determined by extending 20 kb upstream and 10 kb downstream in the reference genome, and 5 kb upstream in the query genome. The length of the extension was based on UAS identification. B Tyfonas-like region was identified through manual inspection. Numerous alignment segments in forward (539 segments, denoted by “+” rhomboids) and reverse (16 K segments, denoted by “-” rhomboids) orientations were observed in the tyfonas-like region on Chr01. Three SV regions with different repetitive sequences were identified, each with clear boundaries determined after manual inspection.

All the aforementioned scenarios suggest that repetitive sequences were responsible for the mapping noise, making the precise detection of SVs dependent on the accurate alignment of these sequences. Instead of focusing solely on aligning repetitive sequences, our approach prioritized identifying uniquely aligned sequences (UAS) adjacent to repetitive regions in both the query and reference genomes (Fig. 3A and Supplementary Figs S18–S21). After precisely defining the UAS, we manually inspected repetitive regions, including complex areas resembling ‘tyfonas’ found in human cancer samples [27]. For instance, we analyzed a tyfonas-like SV region between 9.59 and 11.18 Mb on reference Chr01. Within this 1.59 Mb segment, various callers initially identified 289 SV regions. However, through manual inspection using 15–30 kb UAS, we observed that this region exhibited all three previously described scenarios (Supplementary Table S8 and Supplementary Figs S25–S29). The region contained 16 192 small fragments forming fold-back alignments, each up to 252 bp long, capable of aligning in reverse in both genomes. These fragments could junction with their copies and potentially overlap with other segments, contributing to the highly repetitive nature of the sequences. This abundance of similar sequences led to alignment errors, causing callers to mistakenly identify these misalignments as SVs. Ultimately, we accurately defined three distinct SV regions with specific boundaries: 6160 bp (10 406 708–10 412 867 bp), 45 bp (10 439 305–10 439 349 bp), and 4866 bp (10 548 931–10 553 798 bp) (Fig. 3B). Thus, the use of UAS not only reduces the impact of repetitive sequences, but also enhances the accuracy of detecting complex SVs through manual inspection.

### Base-pair resolution of SV breakpoints

**Figure 4:**
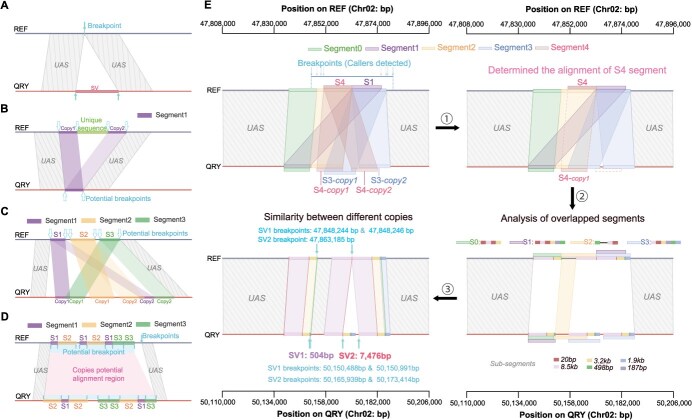
Categories of repetitive sequences for identifying SV breakpoints. A SV breakpoints (arrows) were identified when there were no repeats between upstream and downstream UAS. B Two copies of a segment (represented by rectangles) in the reference genome are joined with a unique sequence that is aligned to a single copy in the query genome. Multiple positions across copies are observed as potential breakpoints (dotted arrows). C A single copy with three segments (S1, S2, and S3) in the reference genome but two copies in the query genome, which may overlap each other. The increased complexity of these repeats raised the number of potential breakpoints. D Multiple distinct copies with three segments (S1, S2, and S3) are present in both genomes. The multiple alignments among these copies resulted in numerous potential breakpoints. E Identifying a complex region on Chr02 revealed multiple categories. Five segments (S0, S1, S2, S3, and S4) were aligned in this SV region. Since S4 is a sub-segment of S2 and overlaps only with S3, one copy of S4 was removed from the query genome to minimize the impact of repeats. Six sub-segments were identified by analyzing the overlapping segments to construct the remaining five segments. Ultimately, two SVs at base-pair resolution were identified.

After determining the UAS, we manually assessed the breakpoints of SVs at base-pair resolution, identifying a total of 1635 SVs. Identifying the positions of breakpoints was straightforward when the sequences were uniquely present in both the reference and query genomes within the SV regions (720/1635) (Fig. 4A and Supplementary Fig. S30). However, determining breakpoints at base-pair resolution became more challenging when repetitive sequences were involved in the SVs (915/1635).

We categorized the repeats into three types during the manual inspection of breakpoints. The first category included unique sequences inserted between two copies or directly connected copies in the query genome, with only one corresponding copy in the reference genome, and vice versa (417/915) (Fig. 4B and Supplementary Fig. S31). The second category involved a single copy with one or more potentially overlapping segments, present once in one genome but multiple times in the other (203/915) (Fig. 4C and Supplementary Fig. S32). The third category consisted of multiple distinct copies with one or more segments found in both genomes (295 out of 915), often characterized by the frequent occurrence of tandem repetitive sequences (46.8%) (Fig. 4D).

It was also observed that complex genomic regions often displayed multiple categories of SV breakpoints. A notable example occurred between 47.84 and 47.88 Mb on Chr02, where nine callers reported 28 SV loci collectively. After manually defining the SV boundaries (47.83–47.88 Mb, covering 41.3 kb), we identified five overlapping alignment segments (S0: 11.9 kb, S1: 12.5 kb, S2: 14.9 kb, S3: 14.5 kb, S4: 12.1 kb) within this genomic region (Fig. 4E). Upon resolving the multiple alignments among these segments, we identified two distinct SVs: one with breakpoints at 47 848 244–47 848 246 bp involving a substitution (3 bp in the reference genome and 504 bp in the query genome), and another at 47 863 185 bp involving an insertion (7476 bp) (Supplementary Figs S33 and S34).

### Definition of SV types

The ambiguous definition of SVs, which is often based on copy number, orientation, or the presence/absence of large genomic sequences, frequently results in discrepancies among callers. For example, copy number variations (CNVs) can sometimes be misinterpreted as insertions. To accurately categorize and convey information about complex SVs, we simplified SV classifications into four basic categories based on sequence features: insertion (INS), deletion (DEL), inversion (INV), and substitution (SUB).

**Figure 5:**
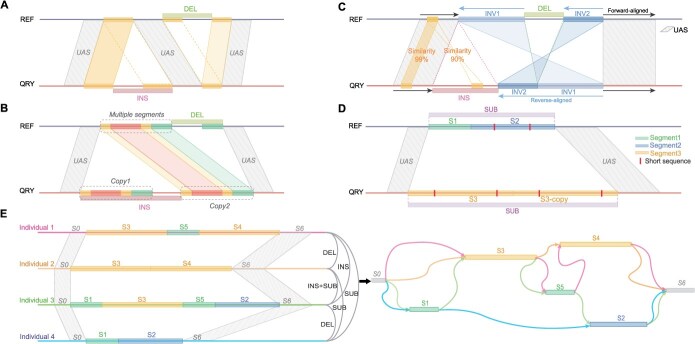
Defining of SV types. A INS and DEL in repetitive regions were identified by the presence of shared sequence segments in both genomes with differing copies. B Identification of INS and DEL in complex regions. A multiple segment consisting of four small segments has two copies (Copy1 and Copy2) in the query genome. An insertion (INS) is identified when the Copy1 junctions with a unique sequence in the query genome, whereas a deletion (DEL) is recognized when a unique sequence junction with a single copy of small segments in the reference genome. C The criteria for identifying INVs are as follows: Forward alignment of adjacent sequences is observed as UAS. A reverse-oriented alignment was excluded due to lower similarity (90% compared to 99% in the forward orientation). Two INVs were identified without any gap sequences. Additionally, INSs and DELs were frequently detected alongside INVs within the same structural variant regions. D Definition of SUBs: two segments (S1 and S2) in the reference genome, along with two copies of a segment (S3) in the query genome, could not be fully aligned. Consequently, they were categorized as SUBs, with potential alignment occurring in shorter sequences. E The importance of SUBs in population genomics. Four individual genomes exhibit varying numbers of three specific segments (S1, S2 and S3; S4 and S5 are the copies of S3 and S1, respectively), leading to structural variations (SVs) such as insertions (INSs), deletions (DELs), and substitutions (SUBs) when comparing genome pairs. By constructing these SVs into a graph-based pangenome, we can effectively represent genetic diversity as insertions or deletions across multiple samples.

INS and DEL refer to the presence and absence of sequences in the query genome compared to the reference genome. Among the 1635 SVs analyzed, the most common types were INSs (780/1635) and DELs (619/1635). While some INSs and DELs were found in simple regions, the majority (60.5%) were influenced by repetitive sequences, either through shared sequence segments with differing copy numbers between the genomes (Fig. 5A) or through varying copies of multiple segments (Fig. 5B). As a result, these variations were sometimes referred to as duplications (DUPs) or CNVs. However, the definitions of DUPs or CNVs often fail to accurately describe the true nature of these variations. For instance, when SVs involve copies containing multiple segments, classifying them as INSs and DELs allowed us to identify the specific segments and copies that form the variant sequences across different genomes, thereby clarifying the repeat patterns of these segments (Supplementary Figs S35 and S36). In contrast, DUPs and CNVs represent entire regions with multiple segments without this level of detailed resolution.

The definition of an INV is straightforward, but identifying INVs is challenging, primarily due to the presence of extensive repetitive sequences [28]. We classify a variation as INV if it meets the following three criteria: the adjacent sequences on both sides of an INV must be forward-aligned between the reference and query genomes; the inverted sequences should be reciprocally the best match; and there should be no assembly gap in the inversion region (Fig. 5C). Based on these criteria, our analysis identified a total of 13 INVs across six chromosomes (Supplementary Figs S37 and S38).

SUB refers to segments in the reference and query genomes that differ and remain unaligned (Fig. 5D). However, this type of SV typically goes unnoticed due to the difficulty of detecting them at base-pair resolution. Through manual inspection, we found that SUBs (223/1635) likely result from recurrent deletions or insertions in one genome, leading to significant sequence differences and complicating the accurate mapping of reads or alignment of genomes (Supplementary Figs S39 and S40). Therefore, SUBs represent a key type of SV in pairwise comparisons. While an SUB identified between two samples may appear as an INS or DEL in a population-wide alignment with other samples, accurately detecting these variations requires sufficient population data. As a result, some variants are classified solely as SUBs due to limited information (Fig. 5E). Once population-level assemblies become available, constructing an accurate pangenome graph will require distinguishing which samples classify the same SV as a SUB versus those classified as an INS or DEL. Integrating this detailed genomic information will enhance the precision of phylogenetic analyses in evolutionary studies.

Defining the four types of SVs based on sequence characteristics improves clarity, but these definitions may not fully capture their biological implications. To address this, our benchmark set provided detailed information for each SV, including breakpoints, types, and the unique alignment sequences from both the upstream and downstream regions in both genomes. This comprehensive representation aims to facilitate their use in biological studies (Supplementary Table S9 and Supplementary Fig. S43).

### Evaluation of callers with SV benchmark

The primary function of the benchmark is to evaluate the performance of different approaches, providing guidance for the development of new algorithms. Currently, the benchmark set of SVs is limited, particularly in its applicability to plant genomes. Therefore, our SV benchmark set allows for more precise comparisons of variant callers.

**Figure 6:**
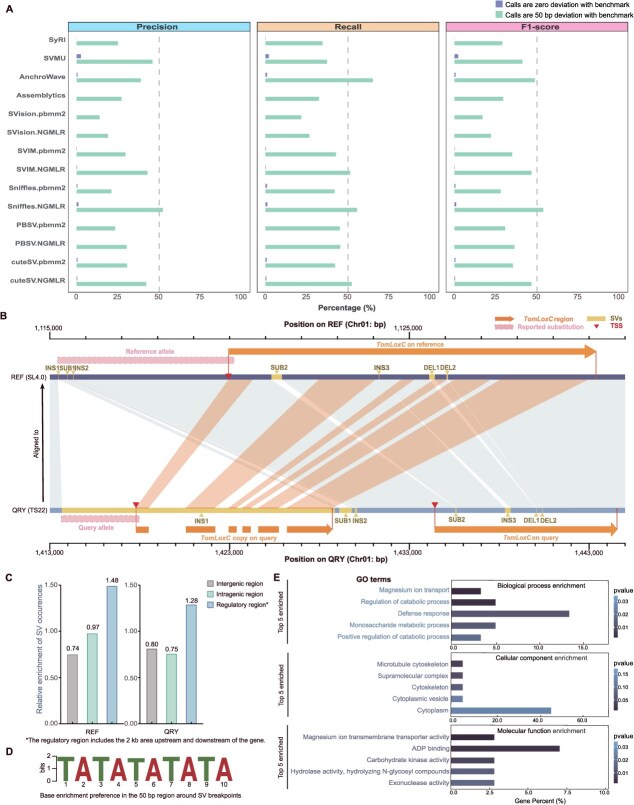
The functionality of SVs. A The performance of different SV callers with SV benchmark, evaluated under two conditions using three metrics. B The new understanding of SVs in the *TomLoxC.* Alignment between the currant tomato TS22 genome and the reference genome revealed two insertions (INS1 and INS2) and one substitution (SUB1) promoter region, along with one substitution (SUB2), one insertion (INS3), and two deletions (DEL1 and DEL2) in the gene region. Another copy of *TomLoxC* in the query genome is located within INS1. The rare substitution in the promoter region is marked with dotted-line rectangles [30]. C The frequency of SVs occurring in intragenic, intergenic, and regulatory regions. The relative rate was calculated by comparing the proportion of SVs present in each region to the proportion of that region within the entire genome. D The sequence motif enrichment preference in the 50 bp region around SV breakpoints. E The function enrichment analysis of the genes overlapping with SVs.

We assessed all 14 detection pipelines based on two distinct criteria. Under the strictest condition, the identified SV loci identified by callers were required to match the benchmark SVs, with no deviation allowed. The results were striking, five pipelines exhibited no overlap with the benchmark set, while the remaining pipelines demonstrated precision rates ranging from 0.04% (SyRI) to 2.56% (SVMU) and recall rates ranging from 0.06% (SyRI) to 2.08% (SVMU) (Fig. 6A). When we relaxed the criteria to allow for a 50-bp deviation from the benchmark and 90% similarity for SV loci to be considered true SVs, the highest precision achieved was only 52.22% (Sniffles with NGMLR) and the highest recall rate reached 65.02% (Anchorwave) (Fig. 6A). In addition, under the relaxed criteria, the overall best performance yielded an F1-score of 53.77% (Sniffles with NGMLR), and no significant differences were found between assembly-based and alignment-based callers.

Overall, since our SV benchmark includes numerous repetitive regions, we observed that these SV callers—developed based on human data—exhibited significantly lower performance metrics on the tomato genome compared to the human genome. When allowing a deviation of up to 1 kb between detected SVs and the benchmark set, previously reported F1-scores typically reported ranged from 0.6 to 0.9 with third-generation sequencing data [29]. However, under the same conditions, our evaluation showed that the highest F1-score achieved was only 53.77%. This highlights the urgent need for highly accurate callers, capable of effectively handling repetitive sequences in complex genomes, such as those of plants, while following a consistent standard to facilitate progress in breeding efforts.

### Functional analysis of SVs

SVs can directly impact phenotypes. For example, previous research has shown that a rare substitution in the promoter region of the *TomLoxC* gene affects tomato flavor by altering apocarotenoid production [30]. However, another study identified a duplication at the *TomLoxC* locus, highlighting the complexity of this genomic region beyond what is currently understood [31]. To explore this further, we manually validated this SV using 25 assemblies from the published tomato pangenome [32] (Supplementary Table S10).

We identified variations in seven samples within the gene and promoter regions compared to the reference genome, including four currant (TS22, TS265, TS413, and TS421), one cherry (TS623), and two cultivated (TS12 and TS331) tomato genomes (Supplementary Tables S11 and S12). In contrast to the previously identified substitution (reference allele 4724 bp, non-reference 4151 bp), located 149 bp upstream of the transcription start site (TSS), our results revealed that all samples except TS12 contained a 15 kb insertion (INS1), a substitution (SUB1, with 1 bp in the reference and 699–711 bp in samples), and a 133 bp insertion (INS2) within the region from 4325 to 4743 bp upstream of the TSS on the reference genome. Both SUB1 and INS2 were encompassed within the previously identified reference allele. Interestingly, these two SVs in the query genomes did not correspond to the non-reference allele sequence. However, INS1 was located 2 bp upstream of the reference allele, overlapping with both the non-reference allele and part of the query genome sequence (Fig. 6B).

Furthermore, INS1 contains a copy of the *TomLoxC*, as evidenced by seven segments originating from the gene region. This copy was absent only in sample TS12, which led to the discovery of four new SVs in the promoter regions. Samples TS413 and TS265 showed two additional INSs: one measuring 19 kb and the other 327 bp. Across all samples we identified seven distinct SVs in the gene region across all samples, including one SUB, two INSs, and four DELs. In total, we identified 16 new SVs within the *TomLoxC* region, providing a foundation for future genome-wide association studies on tomato flavor (Supplementary Figs S44–S51).

To explore the potential mechanisms behind SV formation, we analyzed the functional features of breakpoints and found that SV occurs more frequently in gene regulatory regions (relative enrichment of 1.48) compared to gene bodies (0.97) and intergenic regions (0.74) (Fig. 6C). Sequence motif analysis within 50 bp of the breakpoints revealed a preference for SV occurrence in AT-rich regions (Fig. 6D). Additionally, we identified 477 genes overlapping with 1635 SVs in both genomes. Gene ontology (GO) analysis showed that these genes were enriched in processes related to defense responses (Fig. 6E). These findings suggest that SVs predominantly affect gene regulatory regions and may play a role in adaptation mechanisms.

## Discussion

Through manual correction of the copy alignment between two cultivated tomato genomes, we curated an SV benchmark resolved at the base-pair level and uncovered the challenge of detecting SVs in repetitive regions. To address current inconsistencies in SV classification, we proposed four basic SV types based on sequence features as a robust standard, which can be easily integrated into a pangenome graph. Notably, substitutions may play a key role in population-level analyses due to their potential involvement in flexible classification as insertions or deletions across different samples.

Our benchmark revealed an underlying issue. While several functional SVs identified in previous research appear to support the accuracy of current detection methodologies, genome-wide detection still includes a substantial number of false identifications. Even with our manual inspection, undetected false-negative SVs and erroneous detections may still persist. These limitations primarily originate from three factors. First, our inspection relies on data generated by existing SV callers, inherently leading to the omission of regions where no variations were reported. Second, current alignment tools remain imperfect, making it difficult to fully correct alignment-induced errors. This challenge is particularly pronounced in repetitive sequences, especially in tandem repeat regions, where complexity often leads to ambiguous identification of repetitive units (Supplementary Figs S41 and S42). Third, although we excluded regions with known large-scale assembly errors and gaps, unidentified assembly inaccuracies may still influence the accuracy of SV identification, which is based on genome-to-genome alignments.

Additionally, our research focused on a single tomato sample closely related to the reference genome, leaving the exploration of special SV regions in populations and other species unaddressed. Furthermore, our proposed manual process is not scalable for large population analyses. We anticipate these limitations to be mitigated by future advancements in next-generation aligners and deep-learning-based SV algorithms, which could directly construct a reference-level pangenome graph encompassing all variants from multiple genomes within a species. Our benchmark dataset represents a valuable resource for training artificial intelligence algorithms, offering the potential to enhance the accuracy of SV detection. Moreover, key strategies from our manual inspection pipeline, such as employing UAS to confirm SV boundaries and utilizing copy similarity for breakpoint identification, could also inform the development of advanced computational methodologies for SV analysis.

The precise resolution of SVs at the base-pair level provides invaluable insights into their inherent characteristics, including the surrounding sequences and their potential biological impacts. The establishment of standardized classifications aids in achieving consistency in detection algorithms, thereby improving SV benchmarks and facilitating the sharing of SV databases within plant genomics. In the future, we anticipate that advancements in SV detection algorithms will enable the accurate identification of SVs at the population level, facilitating a comprehensive understanding of how SVs influence growth, development, and adaptive evolution.

## Materials and methods

### Plant materials and sequencing

The cultivar tomato VF36 was utilized for high-fidelity (HiFi) long-read sequencing with the PacBio sequel II platform in the circular consensus sequencing (CCS) model [33]. DNA samples were extracted from fresh leaves of VF36 seedlings. The sequencing library was prepared using an SMRT bell kit, incorporating an 11.5-kb insertion fragment length. Sequencing was conducted on the PacBio Sequel II instrument using the SMRT Cells 8 M chip. The raw reads were processed into HiFi reads using CCS software (v.3.4.1) (https://github.com/PacificBiosciences/ccs) with parameters ‘--minQ = 0.99 and --min-pass 3’.

### Genome assembly

Three different algorithm-based assemblers HiCanu (v.2.1.1) [34] with the option ‘correctedErrorRate = 0.01’, Flye (v.2.6-release) [35] with the parameters ‘--genome-size = 800000000, --min-ovlp = 5000, and --kmer = 17’, and Hifiasm (v.0.13) [36] with ‘-l = 0’, were employed to assemble the VF36 genome. Subsequently, the contigs were anchored to chromosomes using RagTag (v.2.0.0) [37] with the default parameters. Finally, to facilitate the visualization of complete chromosomes, assembled contigs were integrated and optimized using Genome Puzzle Master (GPM) [38].

### A**ssembly evaluation**

The completeness of the assembled genome was assessed using BUSCO (v.5.3.2) [39] with the parameters ‘solanales_odb10 -m = genome --cpu = 64 --offline’. Genome coverage and accuracy were assessed with Merqury (v.1.4.1) [40] based on k-mer statistics with default parameters. Base-pair level assembly errors for the VF36 genome were examined using CRAQ (v.1.0.9) [24], employing both short and HiFi reads. Errors and heterozygous sites were distinguished by the ratio of alignment coverage to effective clipped reads, classifying errors into clip-based regional errors (CREs) or large-scale clip-based structural errors (CSEs). Two assembly quality metrics, R-AQI and S-AQI, were used to provide insights into the accuracy of the assembled genome at various levels.

### Repetitive sequences identification

Repetitive sequences were identified in both the reference and query genomes to enhance genome annotation and SV calling. Tandem Repeat Finder (v.4.09) [41] with the parameters ‘2 7 7 80 10 50 500 -f -d -m’ was employed to detect all repetitive sequences. Transposable elements (TEs) were annotated using the EDTA pipeline (v.2.1.1) [42] with the ‘—curatedlib’ parameter, which incorporates additional SINE/LINE databases (https://sines.eimb.ru/) to improve the completeness of TE annotations.

### Genome annotation

Protein-coding genes were predicted using the MAKER2 pipeline (v.2.31.9) [43]. Transcriptome quality was controlled using FastQC (v.0.12.1) (https://www.bioinformatics.babraham.ac.uk/pro-jects/fastqc/) with default parameters. RNA evidence was gathered by aligning reads to the repeat-masked assembly using HISAT2 (v.2.2.1) [44]. StringTie (v.2.2.1) [45] was then utilized for transcriptome splicing and integration. Gene structure prediction was conducted through two rounds of training using SNAP (v.2013-02-16) [46], followed prediction with AUGUSTUS (v.3.4.0) [47] utilizing the ‘tomato’ model. Homology-based predictions employed protein sequences from the cultivated tomato Heinz 1706 ITAG4.0 database (ftp://ftp.sol-genomics.net/tomato_genome) and the SwissProt (Viridiplantae) (https://www.uniprot.org). The integrated model with annotation edit distance (AED) values below 0.5 were retained. During the EVM integration of gene prediction results, different weights were assigned to transcriptome evidence, protein evidence, and *de novo* prediction outcomes.

### SV loci detection

We implemented two distinct methodologies to identify SVs from the query genome. For SV calling with HiFi reads, pbmm2 (v.1.1.0) (https://github.com/PacificBiosciences/pbmm2) and NGMLR (v.0.2.8) [11] were utilized to map HiFi reads to the reference genome with parameters ‘--unmapped --preset CCS -N 2’ and default settings, respectively. Subsequently, five read-alignment-based SV callers, PBSV (v.2.4.0) (https://github.com/Pacific-Biosciences/pbsv), SVIM (v.1.4.2) [17], Sniffles (v.1.0.12) [11], cuteSV (v.1.0.10) [14], and SVision (v.1.3.6) [48], processed the alignments from the mappers to call SVs. The assembled query genome was aligned to the reference genome using LASTZ (v.1.04.10) [49] nucmer (v.4.0.0) [50], and minimap2 (v.2.17) [51]. For the assembly-alignment-based methods, SyRI (v.1.3) [52] was used to detect SVs from the minimap2 alignment, with anchors lifted over from the same alignment and SVs subsequently identified by AnchorWave (v.1.1.1) [53]. Additionally, SVMU (v.0.4) [6] and Assemblytics (v.1.2.1) [54] were applied to interpret the results from nucmer and LASTZ alignments.

### SV loci filtering and visual inspection

We retained only the SV loci flagged as ‘PASS’ or ‘PRECISE’ by certain callers, and all loci with variant lengths exceeding fifty base pairs. Following initial filtering, two visualization tools, Integrative Genomics Viewer (IGV, v.2.10.2) [26] and Samplot (v.1.1.6) [25], were employed for further examination of the SV loci. IGV showed read alignments from both mappers, facilitating the observation of depth distribution and the sequence changes in insertions or deletions for each variant. Samplot provided additional insights into inversions and duplications. Consequently, all filtered SV loci were visually inspected and categorized into insertion, deletion, inversion, and complex SVs involving multiple types. False-positive SV loci were excluded from the analysis.

### SV region merging

After applying a visual filter to all SV loci, redundancy was addressed manually. Variants within a 500 bp range were initially consolidated into a single SV region. For longer SV loci potentially containing other SV loci, a length threshold of over 20 kb was temporarily applied for unmerging. Precision in defining SV regions was further enhanced through manual inspection, with the goal of continuously merging adjacent ranges.

### UAS anchor

Manual inspection of base-pair resolution SVs relies on information from two genomes, making it crucial to first identify the SV regions in the query genome. The reads mapped to the merged SV regions in the reference genome were extracted using SAMtools (v.1.9) [55] and then realigned to the query genome by either pbmm2 or NGMLR. Subsequently, the SV regions in both genomes were aligned using MUMmer (v.4.0.0) [50] in ‘dnadiff’ or ‘mummer’ mode with default parameters. The alignments were visualized through manually analyzed or gnuplot (v.5.4) [56], which facilitated the determination of the necessary length of extension or contraction for the SV regions across both genomes, thereby identifying the UAS.

### SV breakpoint identification

To achieve base-pair resolution breakpoints, we initially performed a manual inspection of complex repetitive sequences within SV regions with accurate boundaries, resulting in multiple segments with multiple copies. LASTZ with default parameters was used to confirm the precise positions of all copies. Additionally, the segment units of tandem repeats were identified using blastn (v.2.5.0) [57]. MUMmer was then utilized to calculate the similarity between copies in both genomes, focusing on detecting small variants. Copies showing the highest similarity in alignment were designated as UAS, while the remaining copies were identified as SVs.

### Evaluation of all detection pipelines

We utilized filtered SV loci from all detection pipelines to evaluate their performance. The datasets from SVision, SVMU, Assemblytics, and SyRI were first normalized into a standard VCF format. All datasets were then sorted and compressed using BCFtools (v.1.8) [58] and indexed with tabix (v.1.3.1) [59]. Additionally, our SV benchmark was converted into VCF format. Truvari (v.4.1.0) [60] was employed to evaluate performance using the strict parameters ‘--pctseq=0 --sizemax 30000000 --sizemin 1 --refdist 0 --pctsize 1 --pctovl 1’ and relaxed parameters ‘--pctseq=0 --sizemax 30000000 --sizemin 1 --refdist 50 --pctsize 0.9 --pctovl 0.9.’ and ‘--pctseq=0 --sizemax 30000000 --sizemin 1 --refdist 1000 --pctsize 0.9 --pctovl 0.9.’.

### The functional features of SV breakpoints

We extracted upstream and downstream sequences ranging from 1 bp to 50 bp for each breakpoint. MEME (v.4.12.0) [61] was used to analyze sequence motifs of these sequences with the options ‘-nmotifs = 10 -minw = 2 -maxw = 10’. Furthermore, we assessed the genomic regional preferences of SV occurrences by converting the base pairs at breakpoints into SNPs. These SNPs were then annotated in genic, intergenic, and regulatory regions using snpEff (v.5.2) [62] with default settings.

### Functional analysis of SVs

Bedtools (v.2.29.1) [63] was employed to identify genes overlapping with SVs in both the reference and query genomes using the ‘-wb’ parameter. Subsequently, GO [64] and KEGG [65] pathway analyses were performed to elucidate the biological processes, molecular functions, and cellular components associated with the identified genes.

## Supplementary Material

Web_Material_uhaf107

## Data Availability

The sequencing data and assembly genome generated in this study are publicly available in the Sequence Read Archive (https://ncbi.nlm.nih.gov/sra) under BioProject PRJNA1135477. All SV datasets needed to reproduce the results of this study are available in the Article and Supplementary Data. All code used in the manuscript is publicly available at GitHub (https://github.com/xuecui1997/SVs_inspection).
