# Supplementary material for: The nature of complex structural variations in tomatoes
Source: Hortic Res. 2025 Apr 16;12(7):uhaf107. doi: 10.1093/hr/uhaf107 (PMC12096311; doi:10.1093/hr/uhaf107)
Supplement: Web_Material_uhaf107 [file web_material_uhaf107.zip › Supplementary Data.pdf]

Supplementary Dates for

The nature of complex structural variations in tomatoes

Xue Cui[1,9], Yuxin Liu[1,9], Miao Sun[2,9], Qiyue Zhao[1,9], Yicheng Huang[3,7], Jianwei Zhang[3], Qiulin Yao[4], Hang Yin[2], Huixin Zhang[5], Fulei Mo[5], Hongbin Zhong[6], Yang Liu[1], Xiuling Chen[1], Yao Zhang[5], Jiayin Liu[1], Youwen Qiu[5], Mingfang Feng[5], Xu Chen[1], Hossein Ghanizadeh[1,*], Yao Zhou[2,8,*], Aoxue Wang[1,10,*]

Affiliations:

[1] College of Horticulture and Landscape Architecture, Northeast Agricultural University, Harbin 150030, China

[2] State Key Laboratory of Forage Breeding-by-Design and Utilization, Key Laboratory of Plant Molecular Physiology, Institute of Botany, Chinese Academy of Sciences, Beijing 100093, China.

[3] National Key Laboratory of Crop Genetic Improvement, Huazhong Agricultural University, Wuhan 430070, China

[4] Wuhan Jianbing Technology Co., Ltd., Wuhan, China

[5] College of Life Sciences, Northeast Agricultural University, Harbin 150030, China

[6] Shenzhen CEM Biomedical Technology Ltd., Shenzhen, China

[7] Shenzhen Branch, Guangdong Laboratory for Lingnan Modern Agriculture, Shenzhen Key Laboratory of Agricultural Synthetic Biology, Genome Analysis Laboratory of the Ministry of Agriculture and Rural Affairs, Agricultural Genomics Institute at Shenzhen, Chinese Academy of Agricultural Sciences, Shenzhen 518124, China

[8]Academician Workstation of Agricultural High-tech Industrial Area of the Yellow River Delta, National Center of Technology Innovation for Comprehensive Utilization of Saline-Alkali Land, Dongying 257300, China

[9] These authors contributed equally

[10] Lead contact

[#]Corresponding authors: Aoxue Wang (axwang@neau.edu.cn), Yao Zhou (zhouyao@ibcas.ac.cn), Hossein Ghanizadeh (Wg0003@neau.edu.cn)

Contents

Supplementary note 1: *De novo* assembly and evaluation........ 3
1.1 Assembly pipeline........ 3
1.2 The results of the CRAQ evaluation........ 3
1.3 Verification of eight CSEs........ 4
1.4 The evaluation of assembly completeness........ 5
Supplementary note 2: Identification of SV regions........ 6
2.1 Pipeline for SV loci detection........ 6
2.2 Visualization and SV regions merging........ 6
Supplementary note 3: SV boundaries determination........ 8
3.1 Localization of SV regions in the query genome........ 8
3.2 Analysis of SV regions alignment........ 8
3.3 Determination of SV boundaries........ 9
3.4 Highly complex SV region boundary determination: Tyfonas-like region........ 12
Supplementary note 4: SV breakpoints identification........ 15
4.1 The characteristics of repetitive sequences........ 15
4.2 The analysis of similarity between repetitive segments........ 18
Supplementary note 5: SV types classification........ 19
5.1 Insertions and deletions........ 19
5.2 Inversions........ 20
5.3 Substitutions........ 20
Supplementary note 6: Benchmark........ 22
6.1 Tandem repeat sequences........ 22
6.2 New format........ 22
Supplementary note 7: Identification of SVs in *TomLoxC*........ 24
7.1 The mapping of *TomLoxC* within population........ 24
7.2 SVs detection of *TomLoxC* in population........ 24
Supplementary figures........ 27

Supplementary note 1: *De novo* assembly and evaluation

1.1 Assembly pipeline

For achieving a high-quality chromosome-level assembly of sample VF36, we used multiple *de novo* assemblers, including Hifiasm[1], Flye[2], and HiCanu[3]. To leverage the strengths of these diverse assembly algorithms, we utilized Genome Puzzle Master (GPM) pipeline[4] to integrate all *de novo* assemblies. The cultivated tomato Heinz 1706 version SL4.0[5] was used as the reference genome.

During the GPM editing, the assembled results from Hifiasm showed a higher contiguity with an N50 value of 38.87 Mb (compared to HiCanu at 18.61 Mb and Flye at 10.55 Mb), served as the backbone sequence for manual editing (Table S1). The complete editing process involved several steps: First, contigs from all assemblers were mapped to the reference genome SL4.0, and all contigs were grouped and ordered based on similarity and collinearity across the 12 chromosomes. Second, Hifiasm contigs formed the backbone, supplemented by outputs from Flye and HiCanu to bridge in series wherever possible. Third, considering chromosome location information and complementary potential, complementary contigs were found to fill gaps and merged with Hifiasm assembly to construct the final assembly (Supplementary Fig. 1).

For the nuclear genome, we obtained a chromosome-level assembly of the VF36 genome comprising 44 contigs, with a total length of 798.79 Mb (Table S2). Our assembly exhibits only 32 gaps, a substantial reduction compared to the 259 gaps in the SL4.0 reference genome (31 gaps in SL5.0[6]). This newly assembled genome demonstrates improvements in sequence continuity and completeness relative to the SL4.0 reference genome. The high-quality sequences of VF36 provide a clearer framework for studying variations within the tomato genome.

1.2 The results of the CRAQ evaluation

To validate the accuracy of our assembly, we utilized the Clipping information for Revealing Assembly Quality (CRAQ) algorithm[7] to evaluate the quality of the VF36 genome. By aligning 5X short reads and 30X HiFi reads from VF36 to the assembled genome, we quantified base-pair assembly errors. The analysis revealed two types of errors: Clip-based Regional Errors (CRE) demonstrated an exceptionally low occurrence, with an R-AQI score of 98.34, which exceeds the recommended AQI threshold of >90 for high-quality assemblies; large-scale Clip-based Structural Errors (CSE) were minimally reported, with only eight instances identified across five

chromosomes (Supplementary Fig. 2, Table S3). The infrequency of both error types confirms the reliability of our assembled genome.

1.3 Verification of eight CSEs

To investigate the causes of the eight CSEs, we conducted manual validations. However, for two CSEs found at 47.08 Mb on Chr09 and 54.99 Mb on Chr11, with reported segments of just 2 bp and 21 bp respectively, further validation was considered unnecessary due to their small size and potential widespread alignments across genomic regions. For the remaining six CSEs, since the assembly was anchored based on the reference genome (SL4.0), a detailed sequence feature analysis of the error segments between the two genomes was crucial. This analysis aimed to precisely identify the erroneous assembly intervals and the adjacent sequences contributing to these errors.

Initially, we utilized the blastn[8] algorithm to pinpoint the erroneous segments between VF36 and the reference genome. Subsequently, these error segments were extended by up to 5 kb upstream and downstream on both genomes. Finally, using MUMmer[9] with `dnadiff` mode to obtain alignment results.

As a result, we observed that all CSEs contained segments where the reference genome had a single copy, while VF36 harbored two copies (Table S4). For example, a CSE spanning from 63,440,872 to 63,441,760 bp on Chr01 (899 bp), showed 100 different alignments across the entirety of Chr01 in the reference genome. This suggests that assembly errors may stem from repetitive sequences within this segment. The approximate alignment region in the reference genome was identified from 59,038,922 to 59,039,959 bp on Chr01. After extending by 5 kb, detailed alignment revealed a 1,038 bp segment located between 59,038,922 and 59,039,959 bp in the reference genome, which had two copies linked by a 13 bp sequence in the VF36 genome (Copy1 at 63,440,088 to 63,441,125 bp; Copy2 at 63,441,139 to 63,442,174 bp) (Supplementary Fig. 3a). The region of this CSE be overlapped within these two copies.

To further verify assembly errors, HiFi reads from this two copies region were remapped to the assembled genome. Visualization with the Integrative Genomics Viewer (IGV)[10] showed a 1,051 bp deletion, corresponding to the insertion of 13 bp link sequence and Copy1 in VF36 (Supplementary Fig. 3b). This indicates redundant assembly of the 1,051 bp segment in VF36. Additionally, the interval for CSE1 can be revised to start with Copy1 and end at the linking sequence (from 63,440,088 to 63,441,138 bp).

The remaining five CSEs showed similar patterns, each redundantly assembling a segment once (Supplementary Fig. 4-8). The lengths of incorrectly assembled segments are 570 bp (CSE2, Chr01:67,392,107-67,392,676 bp), 466 bp (CSE3, Chr01:78,624,348-78,624,813 bp), 708 bp (CSE4, Chr03:20,995,230-20,995,937 bp), 1,439 bp (CSE5, Chr03:44,930,457-44,931,895 bp), and 1,972 bp (CSE6, Chr04:65,771,752-65,773,723 bp), respectively.

1.4 The evaluation of assembly completeness

By employing Benchmarking Universal Single-Copy Orthologs (BUSCO)[11] and Merqury[12] to assess the completeness of the assembly. The BUSCO analysis revealed that 98.3% of conserved genes were fully assembled. Utilizing Merqury for k-mer statistics-based identification, we found that 99.3% of k-mers were present in VF36 genome (Supplementary Fig. 9). Additionally, a QV (Quality Value) of 63.895 was calculated, indicating an assembly error rate of only 0.0041%. These results demonstrate that our assembly is highly accurate and well-suited for further structural variations (SVs) identification.

Supplementary Note 2: Identification of SV regions

2.1 Pipeline for SV loci detection

Due to our current inability to achieve base-pair resolution SVs directly through genome alignment, we rely on SV detection software to initially call SV loci. To enhance the detection, we constructed a comprehensive detection pipeline that incorporates nine callers (Supplementary Fig. 10). This pipeline integrates both reads alignment-based approach (PBSV (https://github.com/PacificBio-sciences/pbsv), cuteSV[13], Sniffles[14], SVIM[15] and SVision[16]) and assembly-based approach (Assemblytics[17], SVMU[18], AnchorWave[19] and SyRI[20]). The five alignment-based callers utilize results from two mappers (pbmm2 (https://github.com/PacificBiosciences/pbmm2) and NGMLR[14]) to identify SVs, while the four assembly-based callers used aligners such as nucmer[9], LASTZ (https://github.com/lastz/lastz/), and minimap2[21], which are capable of genome-wide alignment.

After applying quality and length filters (greater than or equal to 50 bp) and excluding non-biallelic sites, we identified a total of 32,128 redundant SV loci (Table S5). Among these, although some insertions are detected at the same positions in the reference genome, the lengths of these inserted segments differ. The counts for callers utilizing pbmm2 were higher than those using NGMLR, with the combination of Sniffles and pbmm2 yielding the highest number of SV loci (3,261, Table S6). Additionally, our analysis revealed that no SV locus was consistently identified by all callers with exactly matching types, breakpoints, and length details.

2.2 Visualization and SV regions merging

Merging SV loci in simple regions is relatively straightforward, typically showing discrepancies of less than 50 bp across different callers. However, in complex regions, significant positional and typological discrepancies are observed among different callers, leading to reduced accuracy in merging. This complicates subsequent manual inspection at base-pair resolution. Consequently, we employ visualization algorithms that represent reads alignments to accurately assess each SV locus and standardize its classification uniformly.

We utilized IGV and Samplot[22] to visualize each SV locus based on alignment data from two mappers, pbmm2 and NGMLR. During manual filtering and correction, we observed that some SV loci detected by callers were outside visualized variant regions or had limited sequences overlapped (Supplementary Fig. 11). Additionally, we removed 2,460 redundant loci that showed no variation (Supplementary Fig. 12). Furthermore, all SV loci were categorized into four primary

types: insertion (INS), deletion (DEL), inversion (INV), and duplication (DUP). SV loci featuring combinations of two or more types were classified as complex.

Following the sorting of remaining SV loci by genomic positions in ascending order, we initially merged loci that were less than 500 bp apart to remove redundancy. The start and end positions of the merged regions were defined by the smallest and largest loci within each range, respectively. However, merging complex SV loci presented challenges in this process, necessitating additional manual adjustments. We identified a total of 4,532 SV regions for further verification (Table S7). Among these, the complex SV regions (711), particularly those with INVs (212), covered extensive genomic regions with high depth distribution and often included multiple types such as DUPs, INSs and DELs (Supplementary Fig. 13-14). Similarly, regions featuring DUP without INVs also exhibited multiple INSs and DELs (Supplementary Fig. 15).

Supplementary Note 3: SV boundaries determination

3.1 Localization of SV regions in the query genome

Identification of SVs through alignment of two high-quality genomes ensures enhanced accuracy. However, the coordinates of SV regions (4,532) are initially derived from the reference genome. Therefore, accurately determining their positions in the query genomes is essential.

To achieve this, each SV region was initially extended by 500 bp upstream and downstream, and reads aligned within these regions were extracted using SAMtools[23] and remapped to the query genome using pbmm2 or NGMLR. Bedtools[24] was then employed to pinpoint the positions of these remapped sequences in the query genome. To mitigate issues arising from short repetitive sequences in complex regions, which can map to multiple genomic locations, we filtered out such alignments. The start and end positions of each SV region in the query genome were determined by the smallest and largest retained alignment sequences, respectively (Supplementary Fig. 16).

3.2 Analysis of SV regions alignment

3.2.1 Pre-filtering of SV regions containing INVs

The initial step of our manual inspection involves analyzing the sequence characteristics of SV regions. We used MUMmer to align SV regions between two genomes. Specifically, for SV regions containing inversions (300), we pre-processed these regions due to their broader spans. We employed `mummer` mode to align SV regions in the reference genome with the entire corresponding chromosomes of the query genome. To mitigate the impact of small-scale repetitive sequences, we applied the `-l` parameter to filter out alignments shorter than 200 bp. Both filtered and unfiltered alignment results were visualized using gnuplot[25], revealing the presence or absence of reverse alignment segments within SV regions (Supplementary Fig. 17). Retaining excessively long regions that lacked clear reverse alignment segments offered no benefit (Supplementary Fig. 17a), as they were neither feasible nor necessary for manual inspection. Consequently, 264 SV regions were removed.

During manual verification, we found that repetitive sequences within these SV regions could align to the query genome in both forward and reverse orientations. The abundance of reverse alignment fragments in the query genome suggests a potential source of errors detected by callers (Supplementary Fig. 17).

3.2.2 Sequence characteristics within SV regions

For the remaining SV regions, we utilized the `dnadiff` mode for alignment and conducted manual visualization of the results. Our findings consistently highlighted the presence of copies with multiple segments in both genomes complicates the detection of SVs.

For example, a complex SV region spanning 19 kb (from 70,659,848 to 70,679,076 bp) on Chr01 was detected by Sniffles and SVision. In the query genome, the corresponding SV region spanned 46 kb (from 75,048,688 to 75,094,946 bp). Detailed alignment analysis using MUMmer identified six distinct alignment segments (Supplementary Fig. 18). Further manual analysis to supplement alignment segments of repetitive sequences revealed that five segments from the reference genome (S1: 3,425 bp, S2: 2,215 bp, S3: 343 bp, S4: 289 bp, S5: 235 bp) each existed in two or more copies within the query genome (Supplementary Fig. 18).

Although we have analyzed the characteristics of repetitive sequences within SV regions, the complex alignments still hinder precise determination of SV breakpoints and types. We believe that a true SV region, whether containing a single SV or multiple nested SVs, should be bordered by unique non-SV sequences both upstream and downstream. However, such sequences are not observed in our current alignment results. It is crucial to verify the presence of additional copies of these segments within SV regions in their upstream and downstream regions.

3.3 Determination of SV boundaries

3.3.1 Locating uniquely aligned sequences

We defined SV boundaries as the interval between upstream and downstream uniquely aligned sequences (UAS), encompassing all copies of multiple segments with varying numbers across both genomes. To accurately determine these boundaries, we adjusted sequences flanking SV regions in both genomes, extending or contracting them as needed to precisely localize the UAS.

For instance, in the case of the SV region on Chr01 mentioned earlier, we extended the upstream and downstream regions by 10 kb across both genomes. Visualizing this extension revealed a 12 kb UAS (70,649,798-70,662,222 bp) in the upstream region (Supplementary Fig. 19). This contrasts with the original alignment results of the SV regions, where previously identified four multiple-copy segments (S2: 2,215 bp, S3: 343 bp, S4: 289 bp, S5: 235 bp) each aligned uniquely (Supplementary Fig. 19a). However, a new repetitive segment (S6: 5.7 kb) emerged, present once in the query genome and twice in the reference genome (Supplementary Fig. 19b). The 10 kb extension also uncovered an additional 12 kb of unaligned sequence in the downstream region of the query genome (Supplementary Fig. 19c). Subsequently, to accurately

locate the UAS in this region, further extension of the downstream sequence of the SV region in the reference genome was necessary.

Following a 36 kb extension, another 13 kb UAS was identified in the downstream region (Supplementary Fig. 20). To this point, all multi-copy segments identified in the original SV region alignment have been optimally unique aligned. Thus, the previously located end position of the UAS in the upstream region was adjusted to 70,669,862 bp, corresponding to a 20 kb UAS (Supplementary Fig. 20c). Simultaneously, a 5 kb segment showing varying copy numbers across the two genomes was newly observed, and the length of S6 increased to 8.7 kb due to the extension.

For clearer results, the upstream sequence in the reference genome was further extended by 28 kb, converting the 18 kb unaligned sequence in the upstream region of the query genome into the UAS. This discovery revealed a total of 38 kb UAS in the upstream region (Supplementary Fig. 21). Ultimately, the end position of the upstream UAS (REF: 70,669,863 bp) and the start position of the downstream UAS (REF: 70,702,571 bp) delineate the SV boundaries.

Additionally, we excluded certain SV regions for two primary reasons. Firstly, upon manual inspection, although the original alignment of these SV regions exhibited varying copy numbers of multiple segments across the two genomes, all copies aligned uniquely after extension. These SV regions were identified as false positives. Secondly, some SV regions overlapped completely after manual adjustment, prompting their consolidation into a single SV region. Besides, we have excluded all SV regions containing gap sequences, totaling 846.

3.3.2 Scenarios in SV boundaries determination

3.3.2.1 Copy number discrepancies

Through manual inspection, we discovered three common scenarios that can cause inaccuracies in detecting SV loci when determining SV boundaries. The first scenario involves copy number discrepancies, where callers identify partial copies of multiple segments within an SV region, resulting in apparent variability in copy number between genomes. However, these regions actually contain no variants, as the copy numbers of multiple segments are consistent across both genomes.

For instance, in the SV region spanning 18,542,473 to 18,553,931 bp on Chr03 (11 kb), SyRI identified an SV locus (DUP) corresponding to the region between 19,705,597 and 19,751,179 bp (45 kb) in the query genome. The longer length of the SV region in the query genome is primarily

due to an abundance of repetitive sequences. Alignment of the SV regions showed that the entire SV region from reference as one segment (S0: 11 kb) completely align to part of SV region in query genome. Moreover, we temporarily masked the alignment of seven small repetitive segments from S0 in the reference genome because they forcibly aligned to the additional 35 kb sequences in the query genome (Supplementary Fig. 22a).

Further analysis revealed that the two subsegments (S1: 6,191 bp, S2: 5,271 bp) within S0 overlapped by 2 kb in the reference genome, each copied twice in the query genome, indicating a duplication event in this region (Supplementary Fig. 22b). However, upon extending the flanking regions of the SV region in reference genome by 18 kb upstream and 17 kb downstream, we discovered another copy of S2 (upstream) and S1 (downstream) in the reference genome between two UAS (Supplementary Fig. 22c). Therefore, accurate determination of SV boundaries helped us verify that there are no variants in this region.

3.3.2.2 Incorrectly identified of reverse-aligned

The second scenario of misdetection arises from SV regions containing INVs. We conducted manual inspections to analyze the sequence characteristics of these regions to identify the reasons for detection errors.

For instance, Sniffles reported an SV locus (INV) spanning from 5.94 to 64.97 Mb (59 Mb) on Chr09, an excessively long region that raised doubts about its accuracy as an SV. To investigate this, we extracted sequences of 1 kb, 5 kb, 10 kb, and 20 kb from both ends of the SV region in the reference genome. These sequences were aligned against the entire Chr09 of the query genome. The results showed consistently optimal alignment positions. Sequences extracted from the start of the SV region consistently aligned from 6,706,591 bp, while those from the end aligned up to 64,385,338 bp. All optimally aligned segments were in the forward orientation, indicating that these positions accurately represent the correct alignment locations of the sequences within the SV region, rather than the INV positions reported by Sniffles (Supplementary Fig. 23).

Additionally, all alignment results revealed numerous reverse alignment segments across the entire chromosome. Focusing solely on the extracted sequence from the beginning of SV region, we observed that most reverse segments from the 1 kb extract sequence aligned at the terminus of Chr09 (Supplementary Fig. 23a). However, as the length of the extracted sequences increased, those within the SV region progressively aligned in reverse to sequences preceding the terminus

of Chr09 in the query genome (Supplementary Fig. 23c-d). The alignment results from the end sequence on SV region exhibited a similar pattern. Although the alignment from the 1 kb sequence was sparse, those from 5 kb to 20 kb sequences displayed concentrated reverse alignments at the beginning of Chr09 (from 1.82 Mb) in the query genome (Supplementary Fig. 23e-h). Consequently, Sniffles might mistakenly recognize these reverse repetitive sequences as the breakpoint of an INV due to erroneous determination of the UAS. Overall, the widespread reverse alignment across the chromosome indicates that callers might detect INVs throughout its entirety.

3.3.2.3 Misalignment of multiple repetitive sequences

The third scenario commonly occurs within highly repetitive regions. The presence of intricate repetitive sequences results in numerous overlapping alignment segments, complicating the accurate identification of genuine SVs. Furthermore, variations in copy numbers of multiple segments across two genomes hinder callers from correctly pairing these copies. Consequently, many SV loci are reported with varying breakpoints and types within a single SV region.

A typical instance was identified on Chr09 from 55,068,947 to 55,095,058 bp, where nine callers detected various types of SV loci such as DELs, INSs, and DUPs within this region. The alignment of SV regions across both genomes revealed multiple copies with thirteen segments. We observed that the segments S1-S6 collectively possess a single copy in the reference genome, while two discontinuous copies exist in the query genome, one of which exhibits lower similarity. A similar pattern is seen in segments S7-S9 (Supplementary Fig. 24a).

Following extensions of 18 kb upstream and 20 kb downstream in the reference genome, along with 5 kb downstream in the query genome, we successfully localized the UAS (Supplementary Fig. 24b). Characterization of sequences spanning from 55,065,876 to 55,100,226 bp across both genomes revealed a significant segment comprising sequences of 1.8 kb (S1), 2.1 kb (S2), and 5.2 kb (S3). In the query genome, this segment is represented by two discontinuous copies (Copy1 and Copy2). However, in the reference genome, we observed only one complete large segment from 55,065,876 to 55,075,142 bp and another incomplete copy lacking the S2 sequence. This incomplete copy could potentially lead to misalignment of the S1 and S3 sequences from the reference to the query genome, resulting in varied SV loci reports by different callers (Supplementary Fig. 24c).

3.4 Highly complex SV region boundary determination: Tyfonas-like region

We discovered a complex region on Chr01, spanning from 9,592,007 to 11,186,704 bp (1.59 Mb). All callers identified a total of 1,397 SV loci within this region, which were consolidated into 289 SV regions (Table S8). These SV regions encompassed various types (INS, DEL, INV, DUP, and Complex), with approximately 81% of those exceeding 10 kb classified as complex types (Supplementary Fig. 25). During the localization of query SV regions, many were found to align with two or more regions on Chr01. For instance, the SV region from 9,592,007 to 9,592,074 bp in the reference genome aligned with both 9.53-9.56 Mb and 10.91-10.98 Mb in the query genome. Moreover, the alignment regions overlapped among SV regions, suggesting that the 1.59 Mb complex region contains numerous copies of repetitive sequences present in both genomes.

We conducted detailed manual inspections of each SV region, categorizing our findings into four sections.

Among the 70 SV regions before 10,373,440 bp, seven false-positive complex SV regions containing INVs were initially excluded. For the remaining SV regions in the reference genome, which aligned with multiple regions in the query genome, alignments starting from the smallest positions in the query genome revealed that these SV regions aligned in the forward orientation without repetitive segments within the upstream and downstream UAS across both genomes. Further analysis of other regions aligned in the query genome showed no identifiable UAS despite adjustments to flanking sequences, indicating misalignment due to repetitive sequences in these regions. All the findings indicate that the 70 SV regions were all false positives with no actual SVs present in the entire region. To confirm this, we aligned the entire SV regions in the reference genome, spanning from 9,592,007 to 10,373,440 bp (78 kb), with Chr01 of the query genome. The alignment revealed that the entire region aligned in the forward orientation to positions 9,553,044-10,334,586 bp in the query genome, showing unique alignments without any detected SVs. (Supplementary Fig. 26). Furthermore, it is evident that between 15 Mb and 16 Mb in the query genome, there exists a fragmented copy of the entire region, consisting of clusters of reverse-aligned segments, each less than 200 bp in length (Supplementary Fig. 26a). This observation underscores that callers were misled by repetitive sequences, resulting in the reporting of false-positive SV loci.

The second section included three SV regions spanning from 10.40 Mb to 10.43 Mb in the reference genome, with only one SV region pinpointed in the query genome, facilitating clear determination of SV boundaries. Two of these SV regions at 10,406,706-10,406,811 bp and

10,412,836-10,412,836 bp established a shared SV boundary (10,406,708 to 10,412,867 bp). Two segments including S1 (53 bp) and S2 (31 bp) were identified within this boundary linked by a 6 kb uniquely aligned sequence, each exhibiting varying copy numbers between the two genomes. Specifically, S1 was found to have two copies in the reference genome and one in the query genome, while S2 displayed the opposite pattern. For the SV region spanning 10,438,760 to 10,438,810 bp, after a 15 kb extension both upstream and downstream in the reference genome, the boundary was defined as 10,439,305 to 10,439,349 bp. Two copies with a 45 bp segment in the query genome. The upstream UAS of this region overlapped with the downstream UAS of the two previously extended SV regions. All the distinct copies with multiple segments causing insertions or deletions.

For the third section, manual inspection of SV regions spanning 10.51 to 10.57 Mb (54 kb) failed to identify UAS downstream even with a 100 kb extension, and the alignment complexity increased with further extension. Consequently, we utilized the `mummer` mode to align the entire SV region from the reference genome to the Chr01 of the query genome for repositioning. Surprisingly, the region from 10.51 to 10.54 Mb in the reference genome aligned with 10.47 to 10.53 Mb in the query genome, while the region from 10.55 to 10.57 Mb corresponded to 13.21 to 13.28 Mb in the query genome (Supplementary Fig. 27a-b). This suggests a large insertion sequence of approximately 2.7 Mb in the query genome, with small fragments within this sequence capable of aligning to multiple positions across both genomes. After filtering noisy repetitive sequences, the SV boundary was clearly determined from 10,548,931 to 10,553,798 bp, with a 30 kb UAS upstream and an 18 kb UAS downstream (Supplementary Fig. 27c-d).

Similarly, the remaining 201 SV regions (10.57-11.18 Mb) aligned to the query genome spanning 13.23 to 13.84 Mb revealed no SVs (Supplementary Fig. 28a-b). There are 2,362 reverse-aligned repetitive fragments within this entire region, each smaller than 219 bp (Supplementary Fig. 28c). Additionally, many sequences were aligned to the 2.7 Mb insertion sequence in reverse, contributing to false-positive detections by callers (Supplementary Fig. 29). Following our manual inspection, we delineated the entire complex tyfonas-like region into three SV regions with distinct boundaries.

Supplementary Note 4: SV breakpoints identification

4.1 The characteristics of repetitive sequences

4.1.1 No repetitive segments

The primary challenge in identifying SV breakpoints at the base-pair resolution is influenced by repetitive sequences present within the upstream and downstream UAS. Therefore, it is critical to analyze the characteristics of these sequences meticulously. An increase in both the number and complexity of repetitive segments can lead to the presence of multiple SVs within a single SV region, thereby complicating the identification of breakpoints.

The most straightforward case occurs in instances without repetitive alignments, exemplified by Chr07 at 19,527,615 bp (INS), identified as an SV region by eight callers. Through manual inspection, the boundary was pinpointed at 19,527,618 bp in the reference genome and from 19,631,471 to 19,633,626 bp in the query genome. Two non-overlapping segments aligned between the genomes without repetitive sequences, defining four breakpoints at 19,527,618 bp, 19,527,618 bp, 19,631,471 bp, and 19,633,626 bp (Supplementary Fig. 30).

4.1.2 The categories of repetitive segments

4.1.2.1 Single repetitive segment

The repetitive segments surrounding SV regions are classified into three categories, each necessitating manual inspection using the `dnadiff` mode of the MUMmer. The first category involves a single segment that appears as two copies within exclusively one genome. For example, in the SV region spanning 7,842,427 to 7,848,018 bp on Chr11, SV boundaries were determined at 7,842,428 to 7,843,453 bp in the reference genome and at 7,848,786 to 7,855,389 bp in the query genome. Two segments aligned with a 1,026 bp overlapped in the reference genome, indicating duplication of this overlapped segment in the query genome, aligning at 7,848,786 to 7,849,810 bp (1,025 bp) and 7,854,376 to 7,855,389 bp (1,014 bp), exhibiting small variants compared to the reference genome (Supplementary Fig. 31). Moreover, these two copies are linked by a unique sequence, serving as an insertion segment in the query genome.

4.1.2.2 Multiple repetitive segments in one genome

The second category involves multiple repetitive segments found exclusively in one genome. As the example on Chr12, two SV regions spanning from 24,824,994 to 24,825,044 bp and from

24,826,948 to 24,826,998 bp shared the same boundary (24,823,838 to 24,831,248 bp) as determined through manual inspection, totaling 7 kb in the reference genome. Detailed inspection of one SV region revealed five segments aligning across both genomes after an 18 kb extension upstream and downstream of the reference genome. These segments (S0: 19 kb, S1: 19 kb, S2: 1.2 kb, S3: 2.2 kb, S4: 1.2 kb) each occur once in the reference genome (Supplementary Fig. 32a). However, further analysis showed that S2 is a sub-segment of S0, and S3 and S4 are sub-segments of S1 in the reference genome, suggesting additional copies of S2 and S3 exist in the query genome. Furthermore, a 1.9 kb overlap between S0 and S1 in the reference genome resulted in the formation of a new repetitive segment (S5), which was also duplicated in the query genome. (Supplementary Fig. 32b).

4.1.2.3 Multiple repetitive segments in both genome

The third category involves multiple copies present in both genomes, often observed alongside other repetitive categories. The region spanning from 47,845,873 to 47,880,655 bp was identified to contain such repetitive sequences. This region comprises five SV regions, with a common boundary established at 47,836,321 to 47,877,712 bp (41.3 kb in the reference genome) through manual inspection. Five segments (S0: 11.9 kb, S1: 12.6 kb, S2: 14.9 kb, S3: 14.5 kb, S4: 12 kb) aligned within this boundary, each overlapped with at least one other segment in both genomes. Therefore, to accurately identify the SV breakpoints, the next step involves systematically analyzing all overlapped segments to deconstruct the complex alignment into simpler sub-segments.

Firstly, we analyzed the repeat patterns of these five segments. We observed that S3 and S4 each have two copies in the query genome but only one in the reference genome, whereas S0 shows the opposite pattern. S1 and S2 each present a single copy in both genomes. Additionally, we noted that S4 is completely a sub-segment of S2 in the reference genome and only intersects with S3 in the query genome. Consequently, we can temporarily mask the copy of S4 in the query genome that are not sub-segments of S2 to simplify the analysis. Among the remaining segments, only S1 overlapped with others in both genomes (Supplementary Fig. 33a). Thus, our objective is to clarify the repetitive sequence characteristics in this region by analyzing the interactions between S1 and the other segments.

In the initial phase, we focused on the overlapped segments among S0 to S3 separately in each genome. In the reference genome, only S1 overlapped with S2 and S3, yielding overlaps of 20 bp (R1) and 12.5 kb (R2) respectively. Additionally, S1 can be subdivided into two sub-segments, R1 and R2 (Supplementary Fig. 33a). In the query genome, S1 completely encompassed S0 and overlapped with S2 by 187 bp (Q1). Furthermore, there was an 8.6 kb overlap (Q2) between S2 and S3.

In the second phase, our goal was to ascertain the alignment of these overlapped segments with others in both genomes to identify additional sub-segments. For instance, by regarding S0 as a sub-segment of S1, we identified a copy overlapped with S2 and S3 in the reference genome, which the aligner did not report. Our analysis revealed that a portion of the S0 sequence overlapped with S2, labeled as R1, while the remaining sequence overlapped with S3 (also overlapped with R2), forming the sub-segment R3 (11.9 kb). Consequently, S0 could be subdivided into sub-segments R1 and R3, while R2 could be further divided into R3 and R4 (662 bp). At this juncture, the composition of S1 was updated to encompass R1, R3, and R4. Likewise, segments R1 to R4 identified in the reference genome were also detected in the query genome, with R1 temporarily exhibiting two copies (Supplementary Fig. 33b).

Subsequently, we analyzed the remaining overlapped segments Q1 and Q2 in the query genome. Q1 is naturally present as two copies in the reference genome arising from the overlap between S1 and S2. The copy overlapped with S1 also constitutes a sub-segment of R4, thereby enabling the subdivision of R4 into Q1 and R6 (475 bp). The identified of Q2 in the query genome included a R1 at the end, stemming from the overlap between the end of S2 and the beginning of S1 in the reference genome. Alignment of Q2 to the reference genome revealed two copies, one is a sub-segment of S1 and coincides with the sub-segment R3. Thus, Q2 is divided into R5 (8.5 kb) and R1. As a result, S1 is ultimately deconstructed into segments sequentially arranged as R1, R5, R1, R7, R6, and Q1 (Supplementary Fig. 33c).

In the third phase, we deconstructed four segments (S0, S1, S2, and S3), utilizing the sub-segments of S1 as a framework. The analysis indicated that S0 comprises two instances of R1, and one each of R5, R7, and Q1. S2 includes one Q1, one R5, and one R1, while the remaining sequence located between Q1 and R5 was identified as F1 (6.1 kb), unique alignment in both genomes. S3 consists of one each of R5, R1, R7, R6, and Q1, accompanied by a residual segment measuring 1.9 kb at the terminal, designated as F2. Furthermore, the two copies of S3 in the query

genome are interconnected by a 1.5 kb sequence (F3) that also constitutes part of S4 (Supplementary Fig. 34a).

Our meticulous manual inspection has verified that within this intricate SV boundary, seven sub-segments are present in varying copy numbers across both genomes.

4.2 The analysis of similarity between repetitive segments

To identify breakpoints from repetitive segments, our approach involves analyzing the alignment similarity between each copy, with copies exhibiting greater differences classified as SVs. For instance, considering the second category as illustrated by the example on Chr12, four single-copy segments (S2: 1.2 kb, S3: 2.2 kb, S4: 1.2 kb, S5: 1.9 kb) in the reference genome were aligned to their corresponding two copies in the query genome to detect variants, respectively. Using one of two modes from MUMmer, either `show-snps` mode to identify the number of small variants between different copies or `dnadiff` mode to directly assess the similarity of alignment. The results showed that S2 from the reference genome was more similar to S2-copy1 (99.75%, S2-copy2: 87.28%), S3 to S3-copy2 (99.82%, S3-copy1: 88.57%), S4 to S4-copy2 (99.69%, S4-copy1: 89.47%), and S5 to S5-copy2 (99.49%, S5-copy1: 96.98%). Consequently, S2-copy2, S3-copy1, S4-copy1, and S5-copy1 were identified as SVs, with the base-pair resolution breakpoints located at 24,825,045 bp and 24,825,045 bp in the reference, corresponding to 25,598,152 bp and 25,604,508 bp in the query genome (Supplementary Fig. 32c-d).

Likewise, two SVs were identified after calculating similarity in the above case involving Chr02 with seven sub-segments copied across two genomes. Initially, the unique segment F1 (part of S2) was transformed into a new UAS (Supplementary Fig. 34b). Consequently, the upstream region of F1 could be divided into two alignment parts, one consisting of R1, R5, R1, and R7, the other along with a single sub-segment Q1. This configuration leads to a R6 in the query genome become the first SV, with breakpoints at 47,848,244 bp and 47,848,246 bp (QRY: 50,150,488 bp and 50,150,991 bp). In the downstream region, the combination of R7, R6, Q1, F2, and F3 forms the second SV, with the breakpoints identified at 47,863,185 bp and 47,863,185 bp (QRY: 50,165,939 bp and 50,173,414 bp).

Supplementary Note 5: SV types classification

5.1 Insertions and deletions

INSs and DELs not influenced by repetitive sequences are categorized as simple SVs, which are readily identifiable by callers. However, most INSs and DELs are typically mediated by repetitive sequences, often presenting as variations in the number of certain segment copies between two genomes. We classify these repetitive sequences involved in INSs and DELs into two main types.

5.1.1 Interspersed repetitive segments

The first category entails copies of one or more segments that vary in number across both genomes, with segments repeating independently. For example, within the boundary at 32,866,644 to 32,875,843 bp on Chr12, a 1,567 bp segment (S1, spanning 32,874,277 to 32875843 bp) in the reference genome, which has two copies in the query genome linked by a 6,469 bp sequence (Copy1: 33,684,045 to 33,685,613 bp, Copy2: 33,692,083 to 33,693,649 bp). An 18 kb unique segment was closely adjacent to Copy1 (Supplementary Fig. 35a). This SV region is easily classified as a DUP, copy number variation (CNV), or INS, with entire sequences within the boundary in the query genome considered as SV, potentially overlooking the true variant sequences. In fact, our manual inspection revealed another incomplete copy of S1 in the reference genome, missing 395 bp sequence at the beginning of the copy (Supplementary Fig. 35b). Therefore, all copies in the query genome aligned to the complete S1 in the reference genome, with Copy2 exhibiting higher similarity (99.93%, Copy1: 94.72%). Ultimately, the unique segment and the sequence missing from the incomplete copy in the reference genome constitute the variant segments defined as an INS.

5.1.2 Composite segments repetition

The second category involves multiple segments forming a cohesive unit that repeats in both genomes, where the presence or absence of certain segments results in SVs. For instance, within the boundary at 51,600,019 to 51,620,088 bp on Chr08, two complete copies of a large segment consisting of 6 kb (S1), 1 kb (S2), and 0.9 kb (S3) were identified in the query genome (Copy1: 54,324,085 to 54,332,354 bp, Copy2: 54,336,992 to 54,345,203 bp, Supplementary Fig. 36). However, one of the two copies in the reference genome lacked S2, rendering one of S2 in the

query genome as an INS. This classification provides a more precise understanding of the variant segments between the two genomes, offering clarity beyond traditional DUPs or CNVs.

5.2 Inversions

5.2.1 Incorrectly reverse-aligned segments

INVs are relatively rare between two cultivated tomato genomes (13 cases) but are prone to misidentification, especially when multiple copies of segments aligned in both reverse and forward orientation. Consider the region on Chr02 from 29,595,023 to 29,610,742 bp, where the aligner reported two reverse-aligned segments with lengths of 6,221 bp (V1) and 384 bp (V2), respectively (Supplementary Fig. 37). However, in the query genome, both V1 and V2 have forward-aligned copies that exhibit higher similarity (V1: 99.97%, V2: 100%) compared to their reverse-aligned counterparts (V1: 99.71%, V2: 95.47%). Additionally, a reverse-aligned copy in the downstream region of V1 in the reference genome aligns with V1 in the query genome with 99.88% similarity. Notably, there are no unique forward-aligned segments flanking the reverse-aligned ones. Based on our three criteria, classifying these as INVs is not suitable. Therefore, all evidence suggests that the reverse alignments of V1 and V2 are misalignments caused by repetitive sequences. After removing all reverse-aligned segments, a substitution (REF: 10 bp, QRY: 1,050 bp) was identified.

5.2.2 INVs with multiple SVs identification

The regions surrounding INVs are often complex, frequently featuring multiple INVs or various SVs simultaneously, as observed in all 13 cases examined. For instance, within the region from 57,352,484 to 57,352,970 bp on Chr12, there are two consecutive INVs: INV1 (274 bp) ending at 57,352,758 bp and INV2 (212 bp) starting at the same point in the reference genome. No small variations are present between the two INVs, but there is a 4 bp insertion (INS1) between INV1 and the upstream UAS, an 8 bp insertion (INS2) upstream of INV2, and a 65 bp insertion (INS3) between INV2 and the downstream UAS (Supplementary Fig. 38). Therefore, precise base-pair resolution determination of breakpoints is essential for accurate INV identification. Additionally, many false-positive INVs have been identified around genomic gaps, highlighting that telomere-to-telomere (T2T) genome assemblies are beneficial for SV identification.

5.3 Substitutions

Segments on both genomes without alignment were defined as substitution (SUB). Although there were no segments aligned detected by the aligner, manual inspection of the segment sequences revealed three main types of true alignment scenarios.

Firstly, the most stringent scenario occurs when segments from both genomes lack any aligned base pairs. In this case, the segment in the reference genome is considered a deletion relative to the query, and vice versa for an insertion.

The second scenario involves a single base-pair sequence from one genome aligning to multiple positions within a variant segment of another genome. Therefore, classifying this alignment as an INS or DEL would be inaccurate, warranting classification as a SUB. Like the region within the boundary at 55,065,876 to 55,075,142 bp on Chr09, a 9 kb large segment comprising three sub-segments of 5 kb (S1), 2 kb (S2), and 1.5 kb (S3) has two complete copies in the query genome, but one copy in the reference genome lacks S2 and replaces the segment with 1 bp (Supplementary Fig. 39). Thus, the breakpoint in the reference genome is ultimately identified at 55,095,002 bp and classified as a SUB.

The third scenario involves partial alignments between two segments across both genomes, as discovered through manual inspection. The aligner likely missed these alignments due to the short lengths of the segments or the limited sample availability. It means the lack of genetic diversity constrained to current only two genomes; however, segments that are presently unaligned could likely appear as either insertions or deletions in at least one individual within the population. But for now, it can only temporarily define as a SUB. For instance, within the region from 311,175 to 311,208 bp on Chr01, the unaligned segments are 32 bp and 153 bp in the reference and query genomes, respectively. Despite the aligner reporting no alignment between these two segments, manual inspection revealed that 14 bp from the reference could align to the query genome (Supplementary Fig. 40). Such partially alignment situations often involve homopolymers, indicating that repetitive sequences of a single base-pair type could affect the judgment of the aligner. Increasing the population size might allow for a more accurate explanation of SVs.

Supplementary Note 6: Benchmark

6.1 Tandem repeat sequences

Following manual inspection of the SV regions across the tomato VF36 genome, we compiled a dataset containing 1,635 SVs (Table S9). Of these, 14.73% are located in tandem repeat (TR) regions, which are challenging to identify due to their complex sequence patterns.

The features of TR sequences include a single segment serving as the unit with varying copy numbers between the reference and query genomes, where the unit may contain smaller segments. As an illustration, within a TR region spanning from 13.00 to 13.26 Mb on Chr10, we initially aligned sequences in both genomes using blastn, resulting in 32 alignment segments with numerous overlaps. Visual analysis of all overlapping segments revealed that this region comprises multiple copies of four distinct segments (S1, S2, S3, and S4) (Supplementary Fig. 41a-b). This repeating unit consists of one S1, two S2, two S3 and one S4 with a length of 2.6 kb, repeated three times in the reference genome and five times in the query genome (Supplementary Fig. 41c). Breakpoint identification involved comparing the similarity across each copy between the two genomes, revealing the second and third copies in the query genome as an INS with five SNPs in other copies (Supplementary Fig. 41d).

Although we achieved base-pair resolution and determined all repeat patterns in this instance, we recognize that identifying true units in more intricate regions may pose challenges. Aligners may overlook copies of segments if there is substantial disparity between them. (Supplementary Fig. 42a). Additionally, relying solely on similarity for breakpoint identification has its limitations, as cross-alignment of copies may occur (Supplementary Fig. 42b).

Hence, we propose an approach to address these challenges. Through manual inspection, TR regions were recognized as two segments aligned in both genomes with varying overlap lengths, enabling us to identify two copies of a segment overlapped in one genome and one copy in the other genome within this region. This segment can be regarded as a unit. Further calculation of similarity between the copies allows us to identify the non-overlapping sequence from the less similar copy as an INS or DEL with base-pair resolution breakpoints (Supplementary Fig. 42c). To gain a more precise understanding of repeat patterns in TR regions, advanced methodologies are essential to overcome these limitations.

6.2 New format

In contrast to the traditional Variant Call Format (VCF), we arranged the SVs in a tabular format, organized by chromosome and genomic position. The initial eight columns offer detailed information on SV positions in both the reference and query genomes at base-pair resolution. The ninth column delineates SV types, including insertions (INSs), totaling 778 instances ranging from 3 to 126,654 bp in the query genome; deletions (DELs), totaling 619 instances ranging from 13 to 73,144 bp in the reference genome; inversions (INVs), totaling 13 instances ranging from 122 to 214,898 bp in the reference genome and 121 to 214,863 bp in the query genome; and substitutions (SUBs), totaling 224 instances ranging from 1 to 40,197 bp in the reference genome and 1 to 2,700,260 bp in the query genome.

Columns ten to eighteen detail the positions of unique alignment sequences for each SV in both genomes. A "0" marking signifies that this SV shares the same unique alignment sequences with the preceding or subsequent SV or is directly connected to the previous SV without any intervening sequence, suggesting that these SVs were identified from the same SV region. Moreover, we recommend employing graph-based formats like Graphical Fragment Assembly (GFA) or reference GFA for the future storage and management of SV information due to their suitability (Supplementary Fig. 43).

Supplementary Note 7: Identification of SVs in *TomLoxC*

7.1 The mapping of *TomLoxC* within population

We utilized 25 relatively homozygous tomato genomes from previous graph-based tomato pan-genome studies[6] to identify SVs within the gene and the promoter regions of *TomLoxC* gene. This set included genomes from 16 cultivated tomatoes (including the reference genomes Heinz 1706 and VF36), 4 cherry tomatoes, and 8 currant tomatoes.

A previously conducted study identified a rare substitution in the promoter region[26]. To investigate further, we aligned the 10 kb upstream region and the gene region of *TomLoxC* between the reference genome and 27 genomes. The analysis revealed that seven samples, including TS12 (cultivated), TS331 (cultivated), TS623 (cherry), TS22 (currant), TS265 (currant), TS413 (currant), and TS421 (currant), show alignment with repetitive segments due to the presence of variants in both regions, with a higher incidence observed in currant tomatoes (Table S10).

7.2 SVs detection of *TomLoxC* in population

The manual inspection was utilized to meticulously analyze the SVs in these seven samples. To accurately determine the boundaries, it was first essential to ascertain the SV regions in the query genomes. Given the considerable divergence in the *TomLoxC* and its upstream region among the samples, resulting in fragmented alignments. Thus, the longest segment aligned in the query genome was designated as the SV region.

For the cultivated tomato TS331, the longest aligned segment spanned 5,520 bp (1,743,468 to 1,748,987 bp) and mapped to the reference genome from 1,119,976 to 1,130,190 bp. We extended 0 bp, 5 kb, 20 kb, and 15 kb upstream and downstream of this SV region in the reference and query genomes respectively, eventually pinpointing the boundary at 1.11 to 1.12 Mb. Our analysis identified three SVs in the promoter region, including two insertions (INS1: 15 kb, INS2: 133 bp) and a substitution (SUB1, REF: 1 bp, QRY: 703 bp) (Supplementary Fig. 44). Two additional SVs were found in the gene region involves a substitution (SUB2, REF: 291 bp, QRY: 5 bp) and a deletion (DEL3: 38 bp, Table S11).

Similarly, for the cherry tomato TS623, the SVs were identical to TS331 in both the promoter and gene regions, except for a 4 bp reduction in INS1 (Supplementary Fig. 45, Table S11).

Among the four currant tomato samples, greater differences were observed compared to cultivated and domesticated tomato species. Sample TS22 exhibited the same SVs in the promoter

region but had two additional SVs identified in the gene region, including an insertion (INS5: 317 bp) and a deletion (DEL2: 158 bp) (Supplementary Fig. 46, Table S11).

Sample TS265 exhibited no SVs in the gene region aligned to the reference genome (Supplementary Fig. 47, Table S11). However, in the promoter region, besides INS1 (15,122 bp), SUB1 (REF: 1 bp, QRY: 699 bp), and INS2 (133 bp), an additional 327 bp insertion (INS4) was identified downstream of these SVs, starting at 1,117,029 bp in the reference genome, approximately 2.9 kb from the transcription start site (TSS).

Sample TS413 displayed further deviations. In addition to INS1 (15,147 bp), SUB2 (REF: 1 bp, QRY: 701 bp), and INS2 (133 bp), a 19,138 bp insertion (INS3) was identified 902 bp upstream from the TSS. The SV landscape within the gene region differed notably from other samples, featuring a new deletion (DEL1: 799 bp) and a new insertion (INS6: 69 bp) in addition to DEL3 (Supplementary Fig. 48, Table S11).

SVs in sample TS421 were simpler, exhibiting the three SVs (INS1: 15,178 bp, SUB1: 701 bp, INS2: 133 bp) in the promoter region and only DEL3 (38 bp) in the gene region (Supplementary Fig. 49, Table S11).

In summary, all six samples consistently showed an INS1 of approximately 15 kb, a SUB1 around 700 bp, and an INS2 of 133 bp in the promoter region, while exhibiting variable SVs within the gene region (Table S12). Notably, INS1 encompassed a copy of *TomLoxC*.

Additionally, in contrast to other samples, the variants observed in the alignment of sample TS12 with the reference genome were not attributable to the copy of *TomLoxC*. The sequence complexity in this region initially hindered precise localization of the gene region in the TS12 genome. Identifying the UAS between TS12 and the reference genome was challenging due to downstream complex repetitive sequences in the TS12 genome (Supplementary Fig. 50). After mitigating the impact of repetitive sequences, the gene region of sample TS12 revealed the presence of SUB2 and INS5 when aligned to the reference genome, along with a new deletion (DEL4 of 100 bp). Notably, SUB2 in TS12 differed from SUB2 in other samples, exhibiting changes in variant segments. It partially overlapped with other SUB2 ranges but also introduced novel variant sequences.

The situation in the promoter region was notably unusual compared to other variable samples, as there appeared to be no additional copy of *TomLoxC*. This discrepancy led to the identification of four new SVs, including three substitutions (SUB3, REF: 2.2 kb, QRY: 616 bp; SUB4, REF:

2.6 kb, QRY: 3.1 kb; and SUB5, REF: 92 bp, QRY: 119 bp) and an insertion (INS7: 228 bp) (Supplementary Fig. 51a). Subsequently, alignment of TS22 with TS12 in both regions confirmed the absence of an additional copy of *TomLoxC* in TS12 (Supplementary Fig. 51b). This case underscores the significance of substitutions as a primary SV type, particularly in population studies.

Supplementary figures

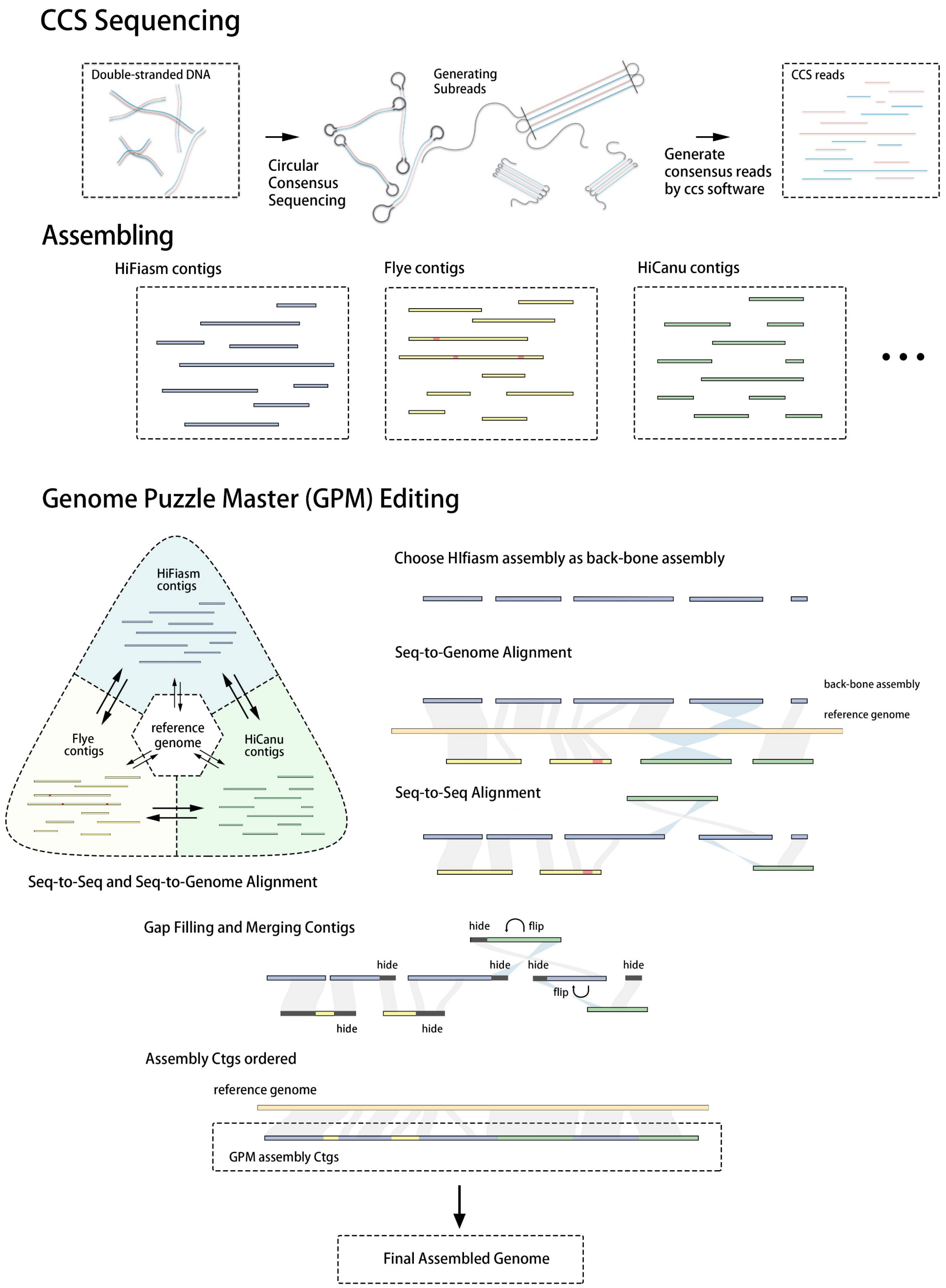

Supplementary Fig. 1 | Pipeline of genome assembly. The contigs assembled by Hifiasm, Flye, and HiCanu were edited using the GPM to produce the final VF36 assembly.

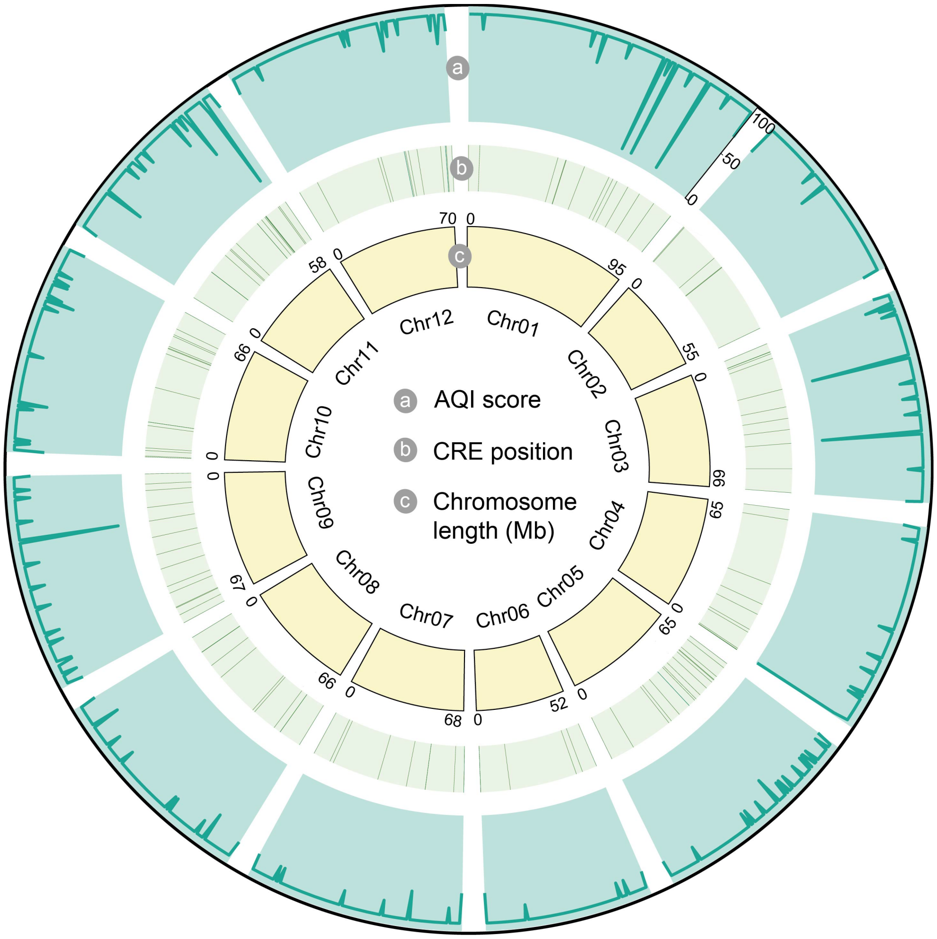

Supplementary Fig. 2 | Assembly quality evaluation of VF36 based on CRAQ results. a, Assembly Quality Indicators (AQIs) score. b, Clip-based Structural Errors (CSEs) positions. c, Chromosome names and sizes.

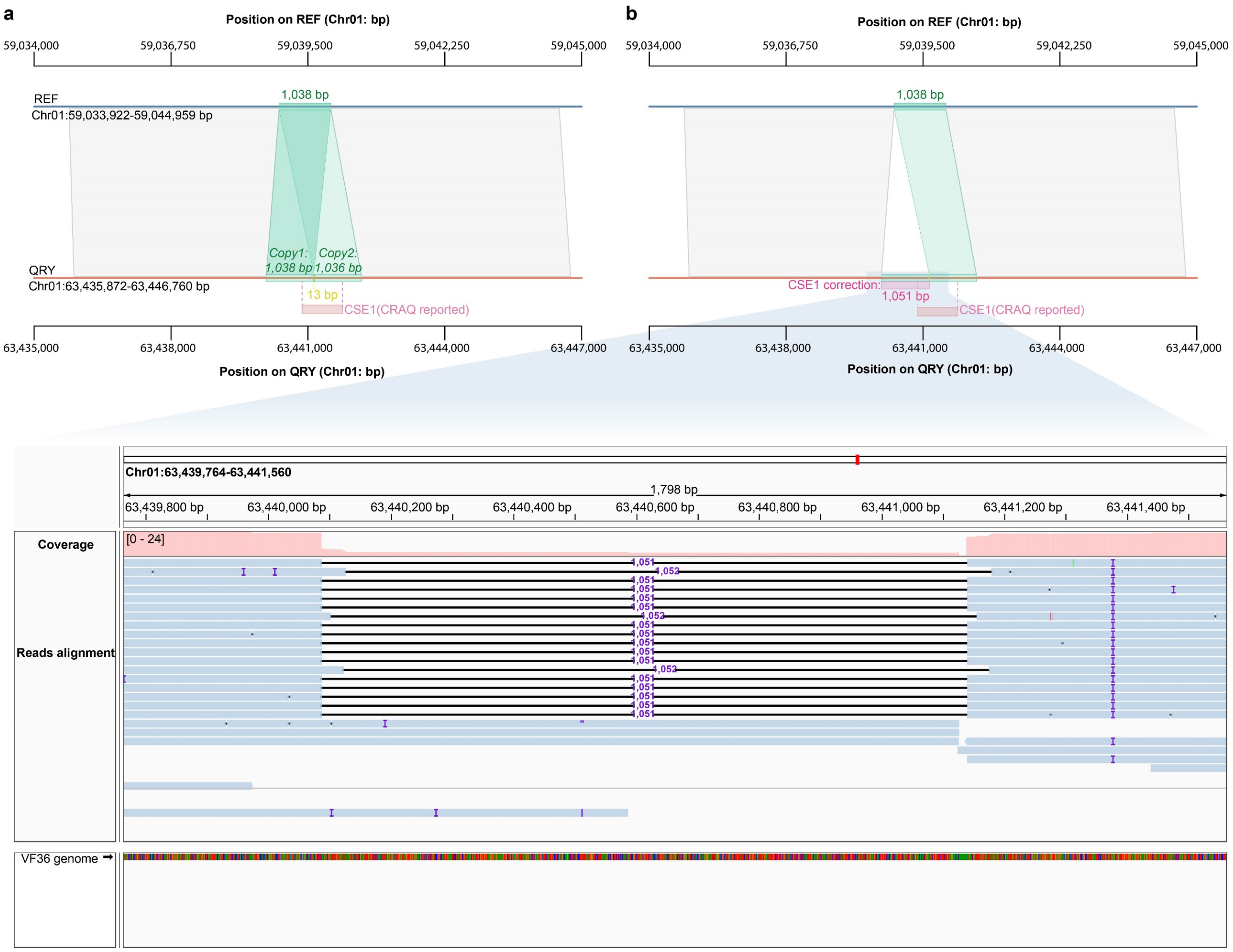

Supplementary Fig. 3 | Manual inspection of CSE1. a) Alignment of CSE1 between the reference genome (SL4.0) and the query genome (VF36) after a 5 kb extension both upstream and downstream. A segment (green rectangles) approximately 1 kb in length appeared as two copies linked by 13 bp sequence (yellow rectangle) in the query genome but as a single copy in the reference genome. **b)** Verification of assembly error within CSE1 and correction of the true error positions. Visualization by IGV of the two copies (blue zoom region) re-mapped to the query genome revealed a 1 kb deletion, indicating that an additional copy of the segment was redundantly assembled by mistake.

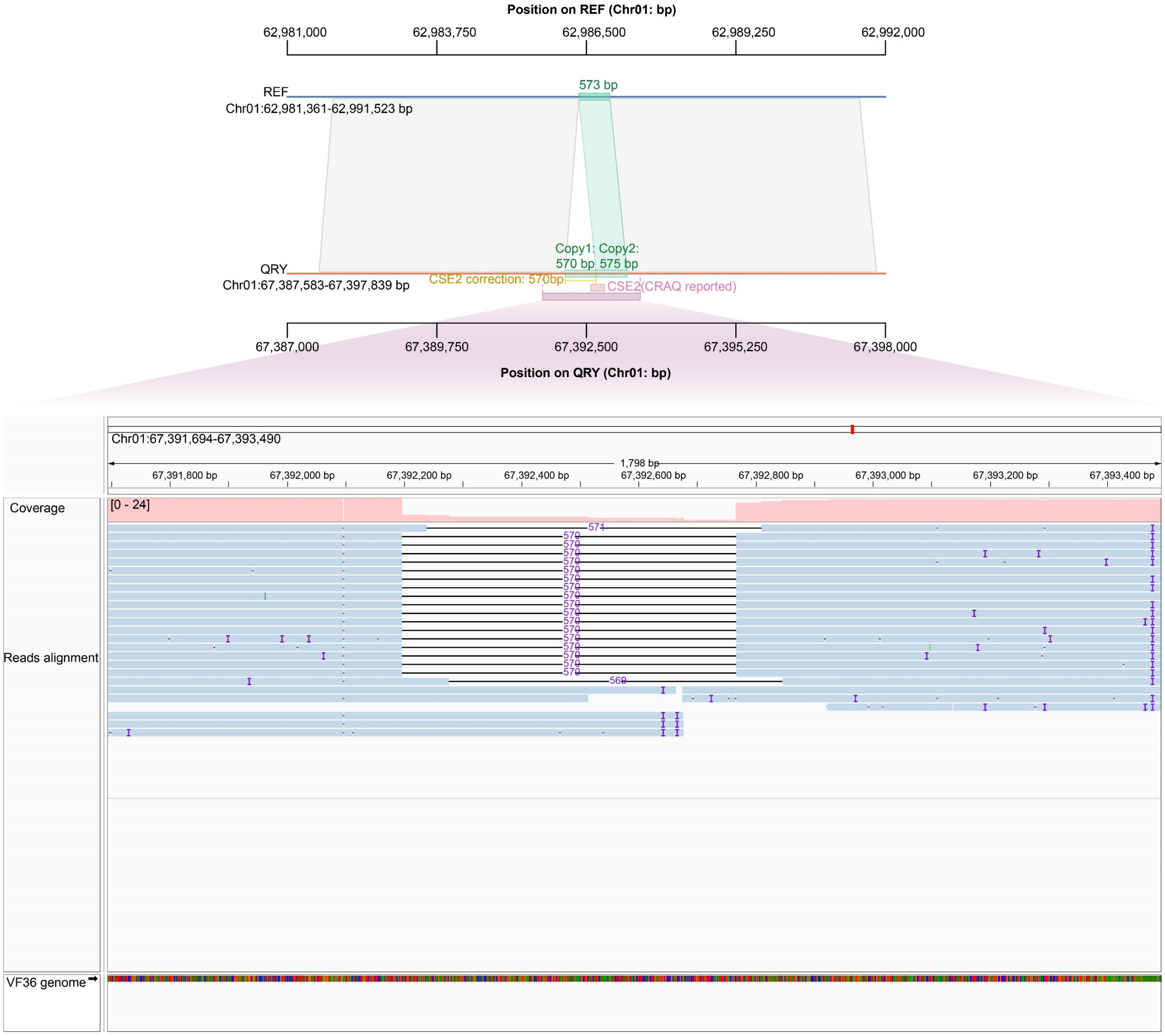

Supplementary Fig. 4 | Verification of the assembly error within CSE2 and correction of the true error positions by visualization.

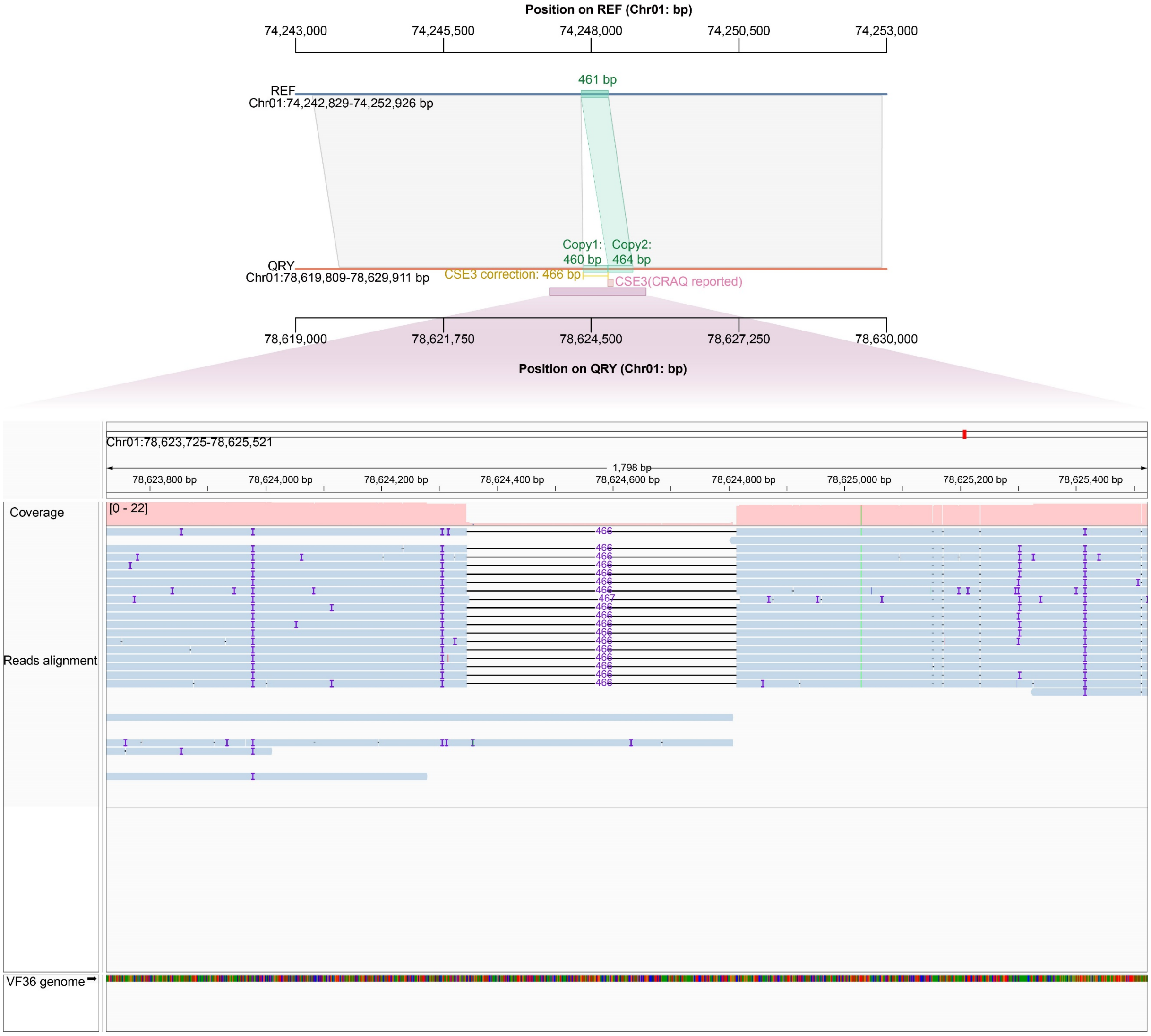

Supplementary Fig. 5 | Verification of the assembly error within CSE3 and correction of the true error positions by visualization.

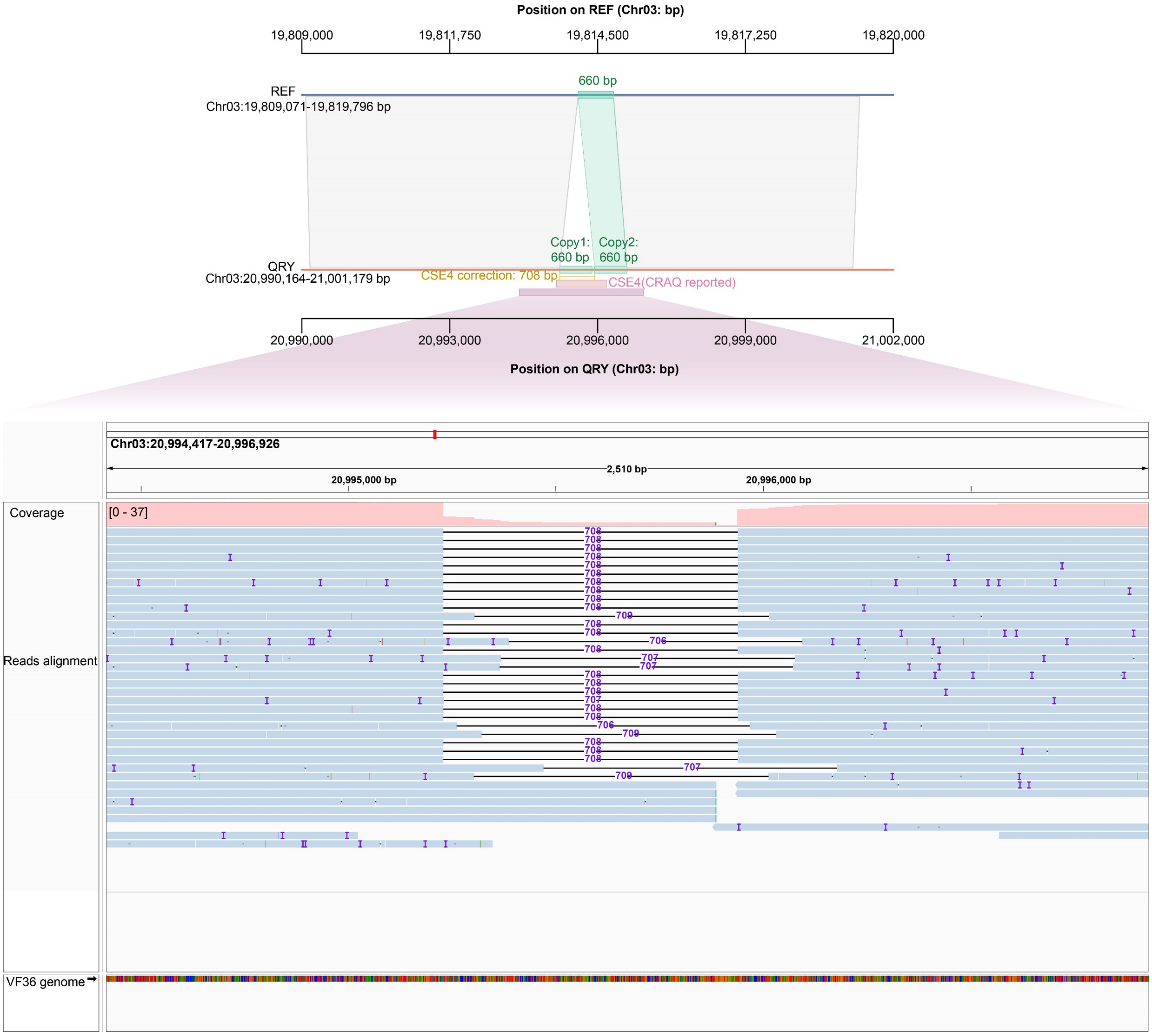

Supplementary Fig. 6 | Verification of the assembly error within CSE4 and correction of the true error positions by visualization.

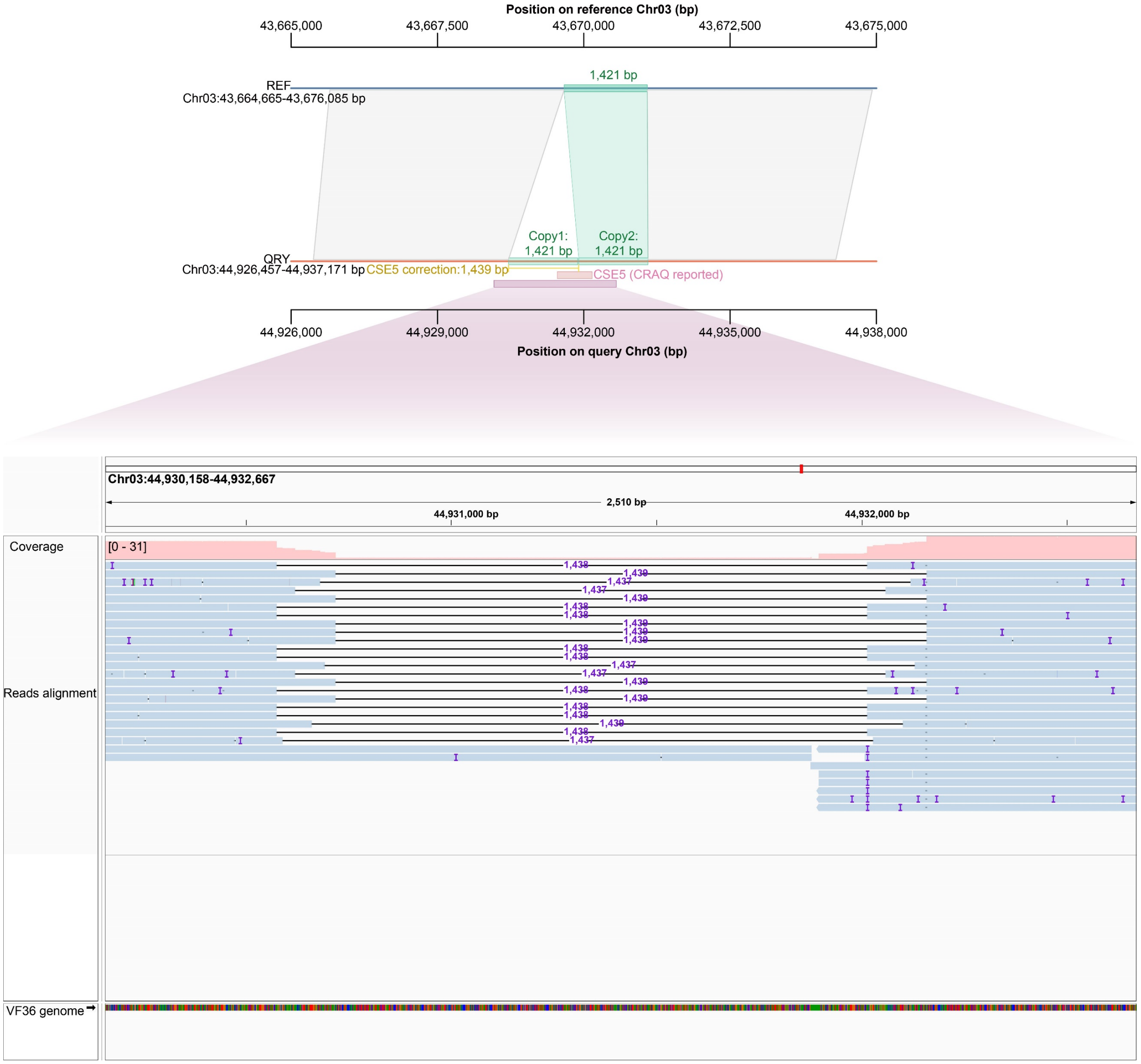

Supplementary Fig. 7 | Verification of the assembly error within CSE5 and correction of the true error positions by visualization.

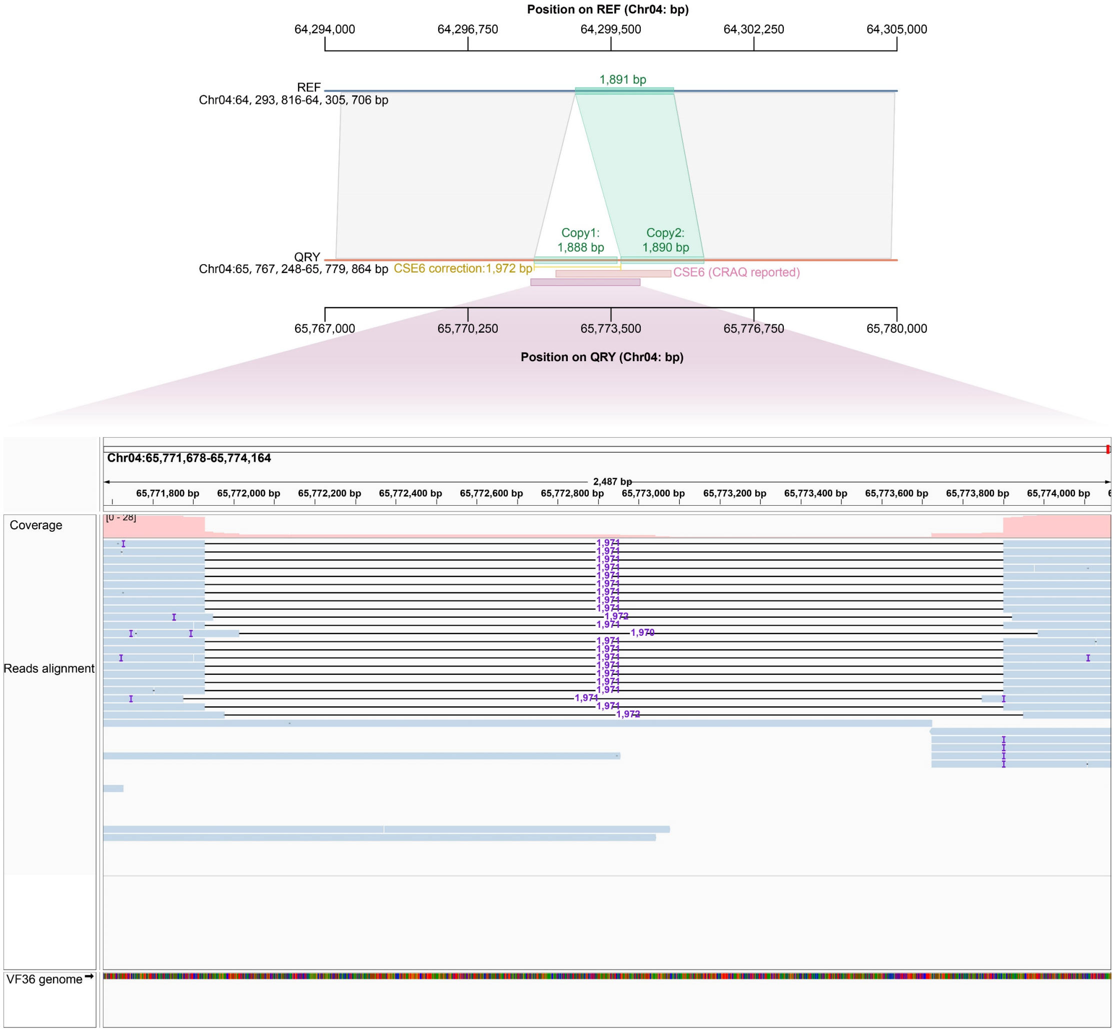

Supplementary Fig. 8 | Verification of the assembly error within CSE6 and correction of the true error positions by visualization.

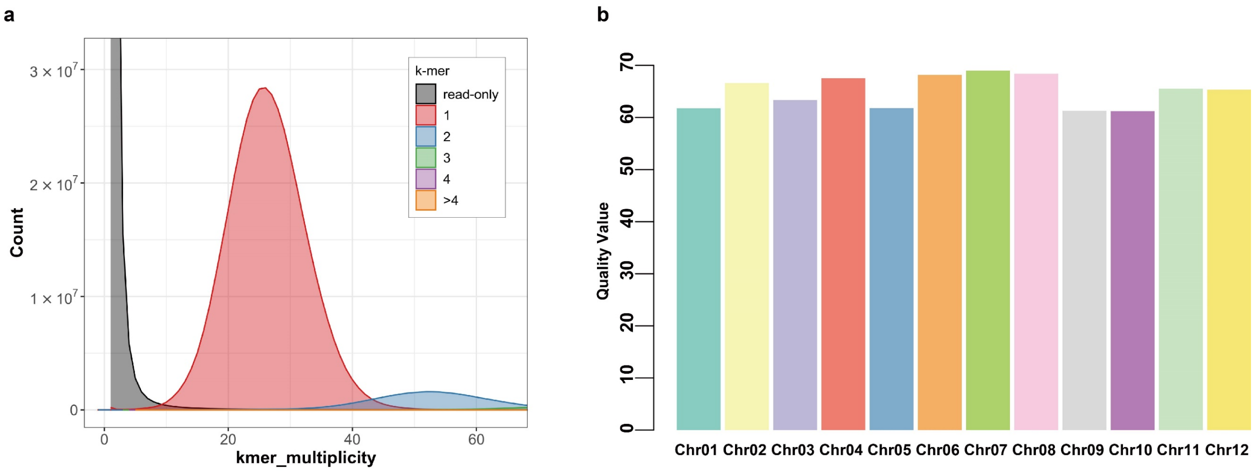

Supplementary Fig. 9 | Evaluation of VF36 assembly. a) K-mer spectrum plots of VF36 assembly. The missing k-mers are depicted in black; k-mers that appear once are depicted in red; blue represents twice etc. The k-mer is set to 19. **b)** The bar chart of Quality Value for each chromosome in VF36.

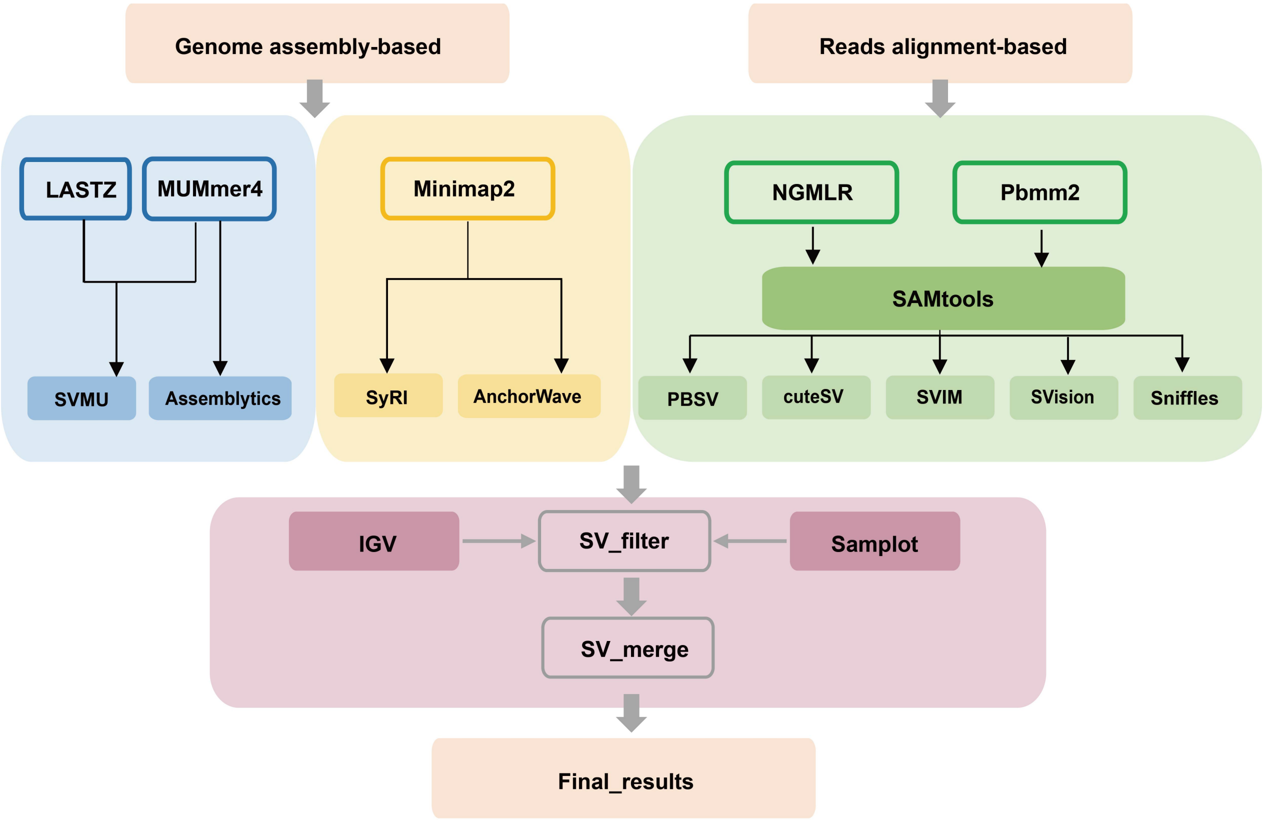

Supplementary Fig. 10 | Overview of the SV regions detection pipeline. Blue box and yellow box represent SV callers and aligners based on genome alignment. Green box represents SV callers and mappers based on read alignment. Purple box represents the pipeline capable of filtering and merging all SV regions.

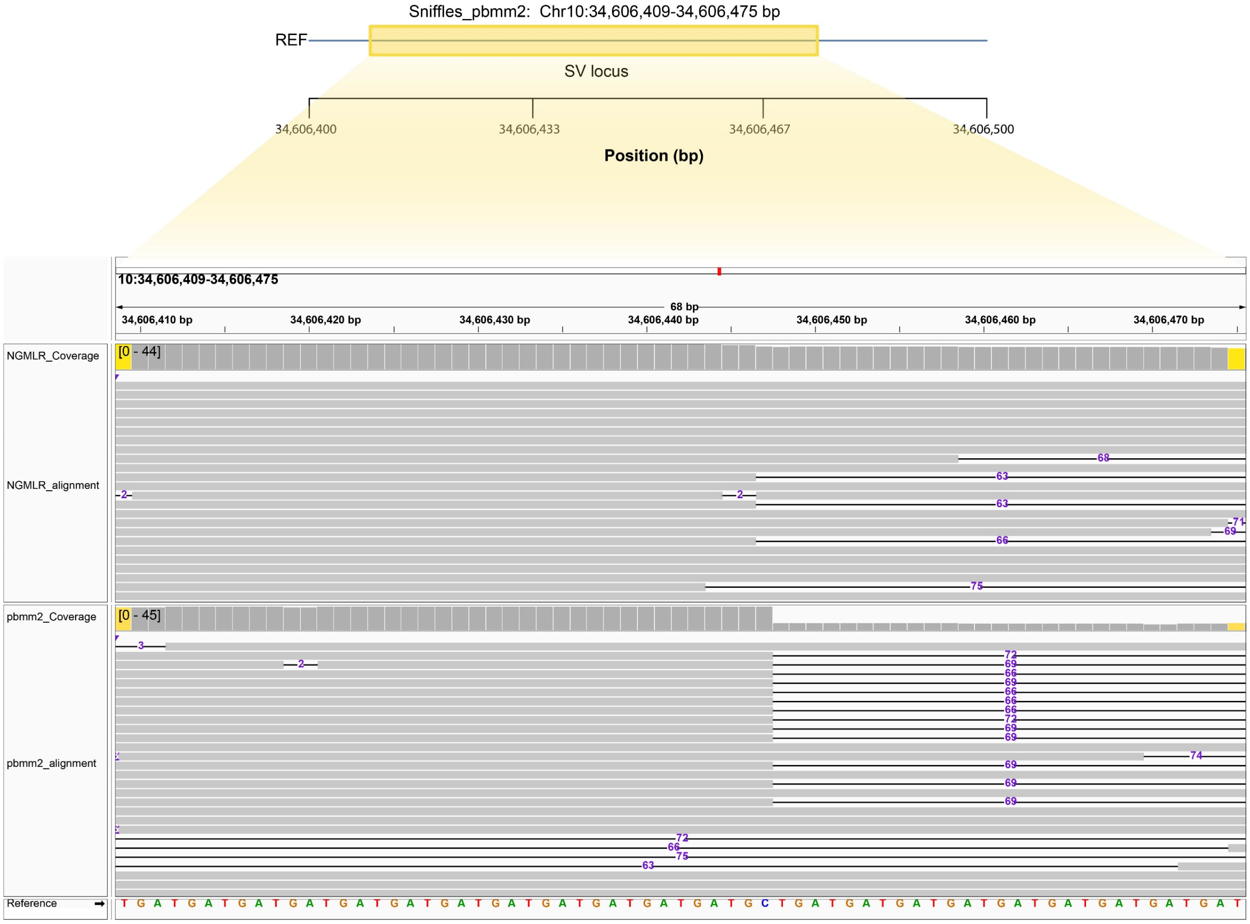

Supplementary Fig. 11 | Deviation of SV loci detected by callers. An SV locus detected by Sniffles with pbmm2 at Chr10:34,606,409-34,606,475 (64 bp) contained only part of the variant visualized by IGV.

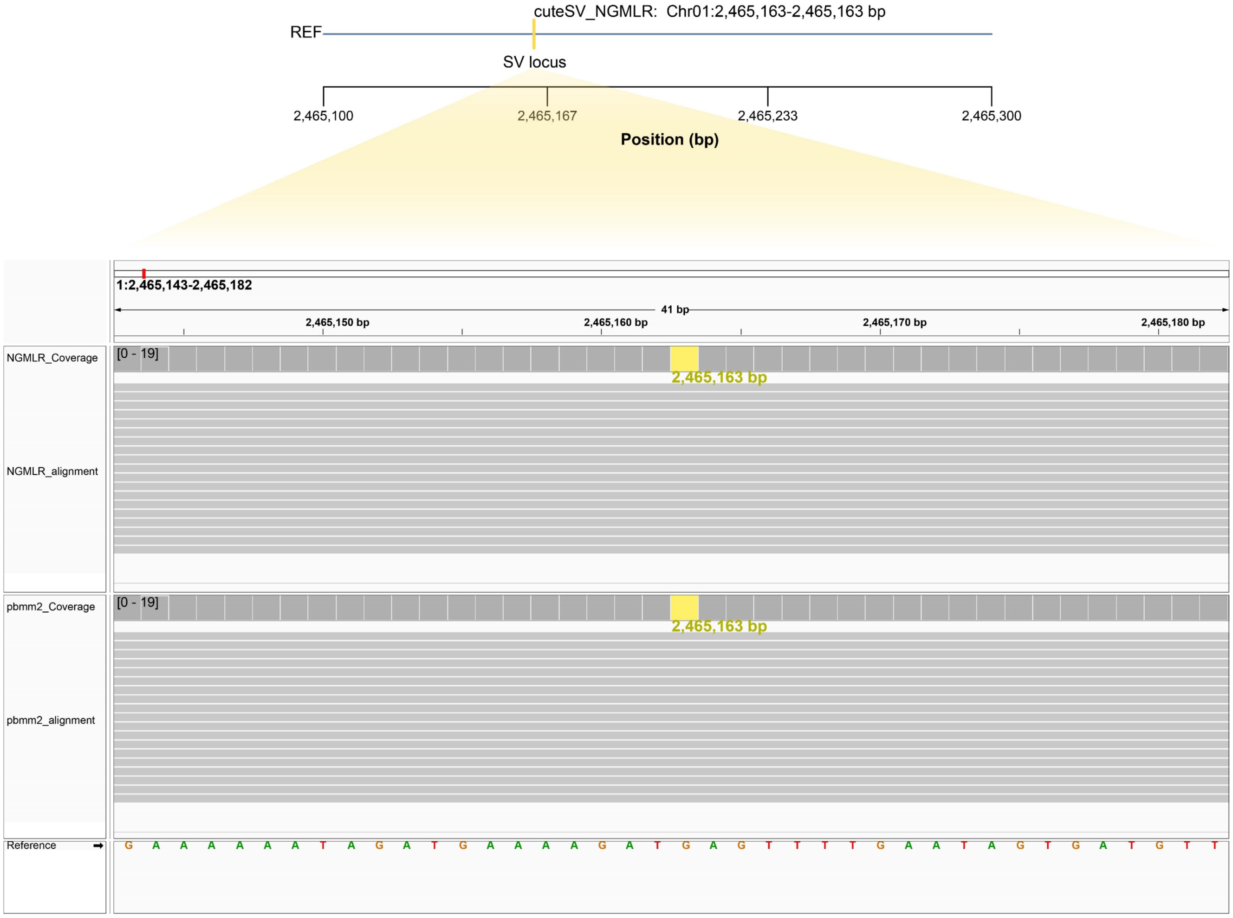

Supplementary Fig. 12 | False-positive SV loci detected by callers. An SV locus detected by cuteSV with NGMLR at Chr01:2,465,163-2,465,163 bp showed no variation upon visualization.

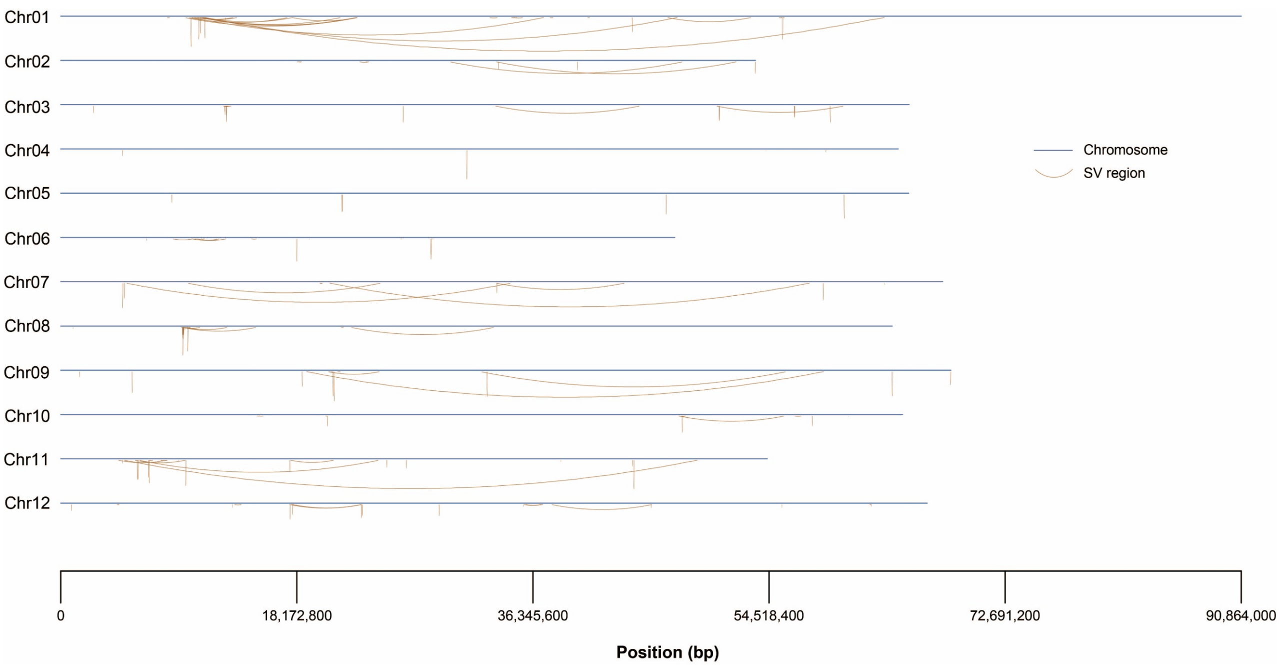

Supplementary Fig. 13 | Distribution of complex SV regions containing INVs across each chromosome. The width of the brown arcs is directly proportional to the size of the SV regions. The blue straight line indicates the 12 chromosomes.

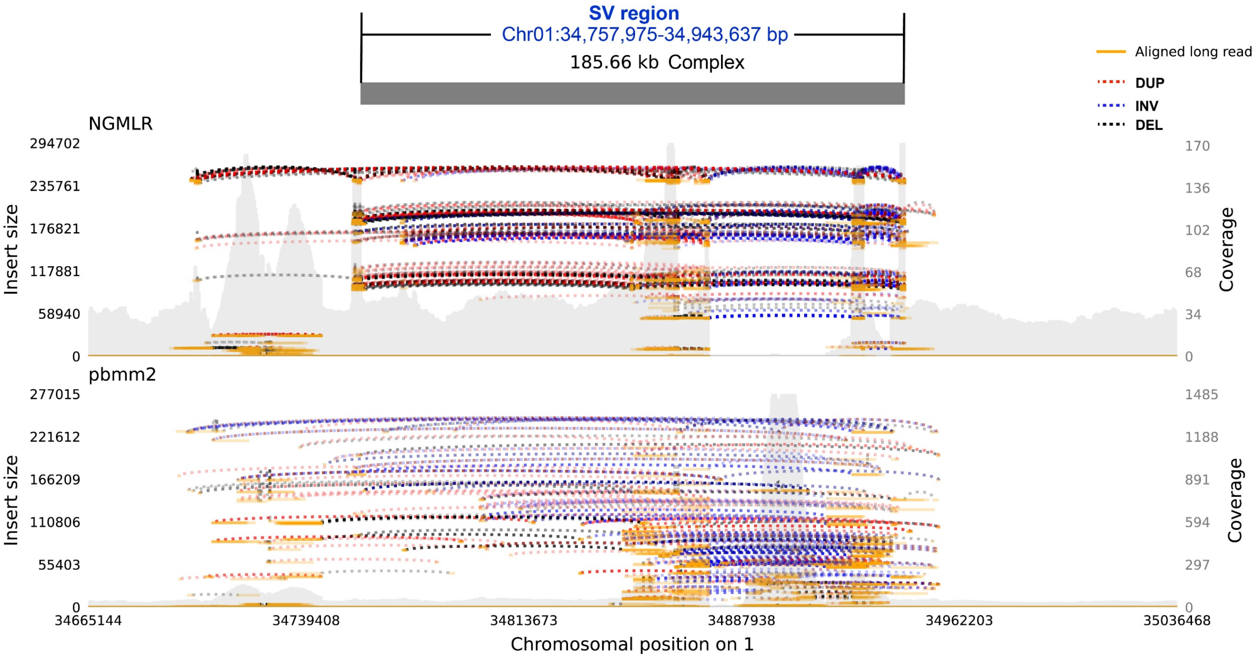

Supplementary Fig. 14 | Multiple SV types in complex SV regions containing INVs. A complex SV region at Chr01:34,757,975-34,943,637 bp (185 kb) exhibits three SV types simultaneously, visualized using Samplot. The grey density curve indicates read coverage.

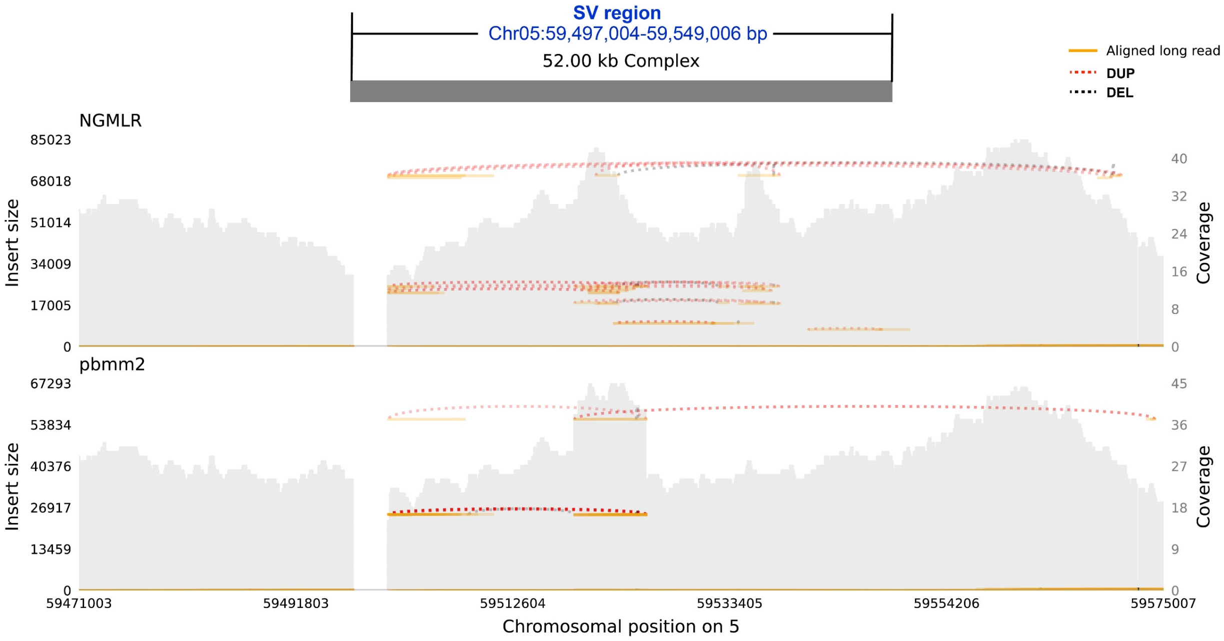

761

762 **Supplementary Fig. 15 | Multiple SV types in complex SV regions without INVs.** A complex
763 SV at Chr05:59,497,004-59,549,006 bp exhibits two SV types.

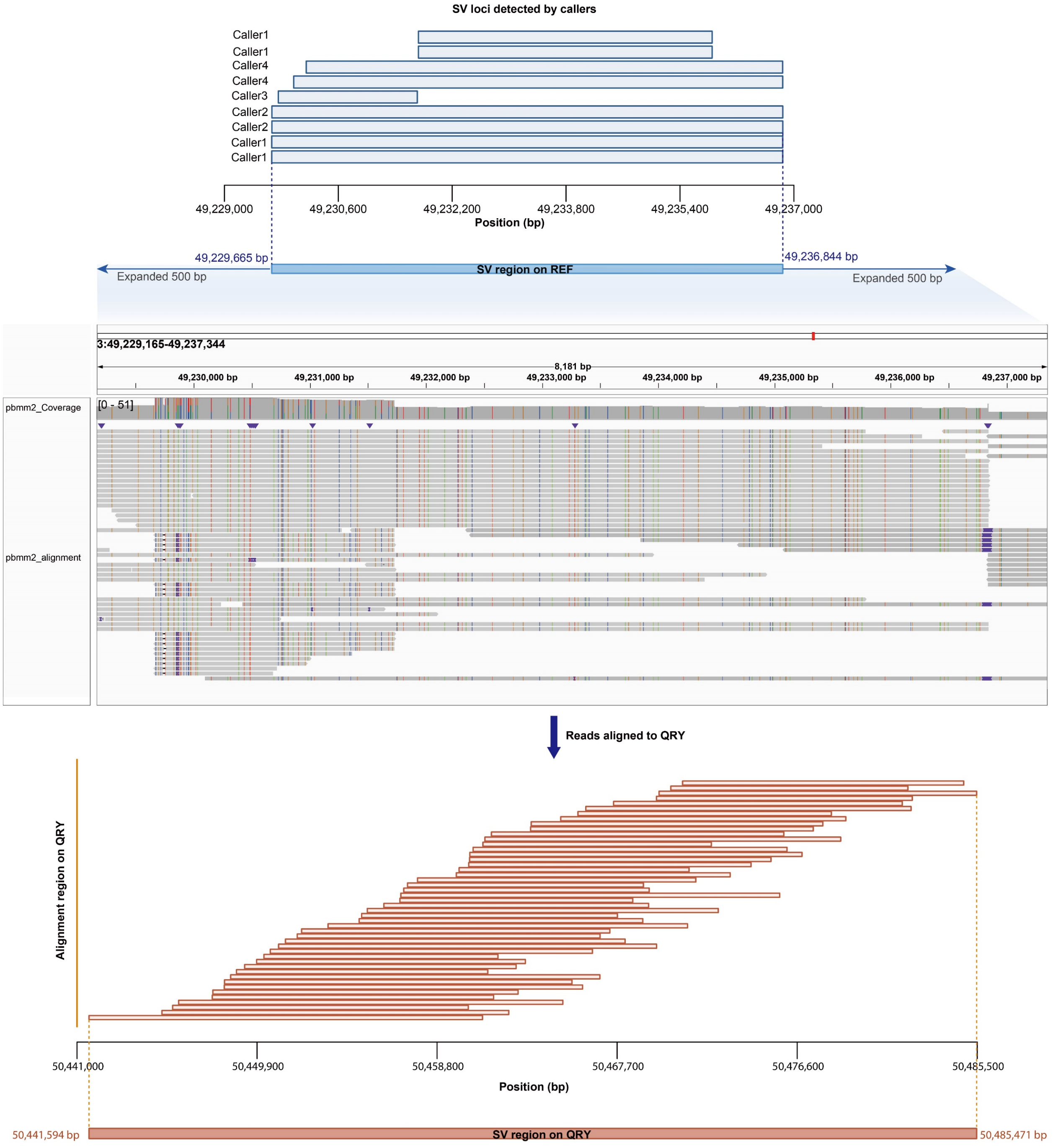

Supplementary Fig. 16 | Methods for obtaining SV regions in the query genome. SV regions in the reference genome were derived from SV loci detected by callers and manually merged. These regions were subsequently extended by 500 bp upstream and downstream. Reads mapped to the reference genome within these regions were extracted and re-mapped to the query genome. The positions of the re-mapped reads determined the positions of the SV regions in the query genome.

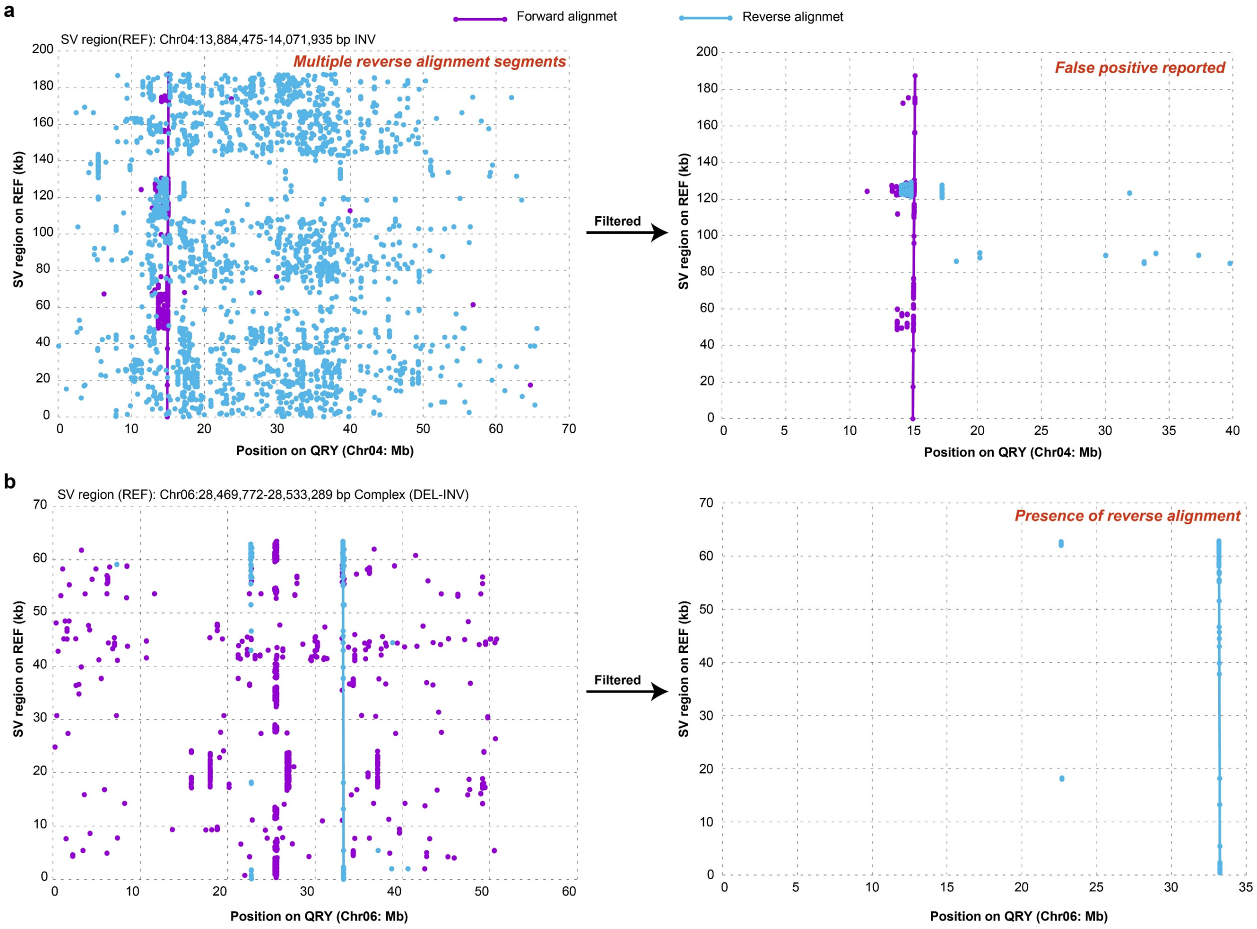

Supplementary Fig. 17 | Alignment of SV region containing INV. a) Visualization of an SV region on Chr04 showing multiple reverse alignments (blue lines) before filtering, which disappeared after filtering out segments shorter than 200 bp. The remaining long forward-aligned segments (purple lines) suggest false-positive reporting of INV by callers. **b)** Presence of reverse alignments observed both with and without filtering, indicating true INV.

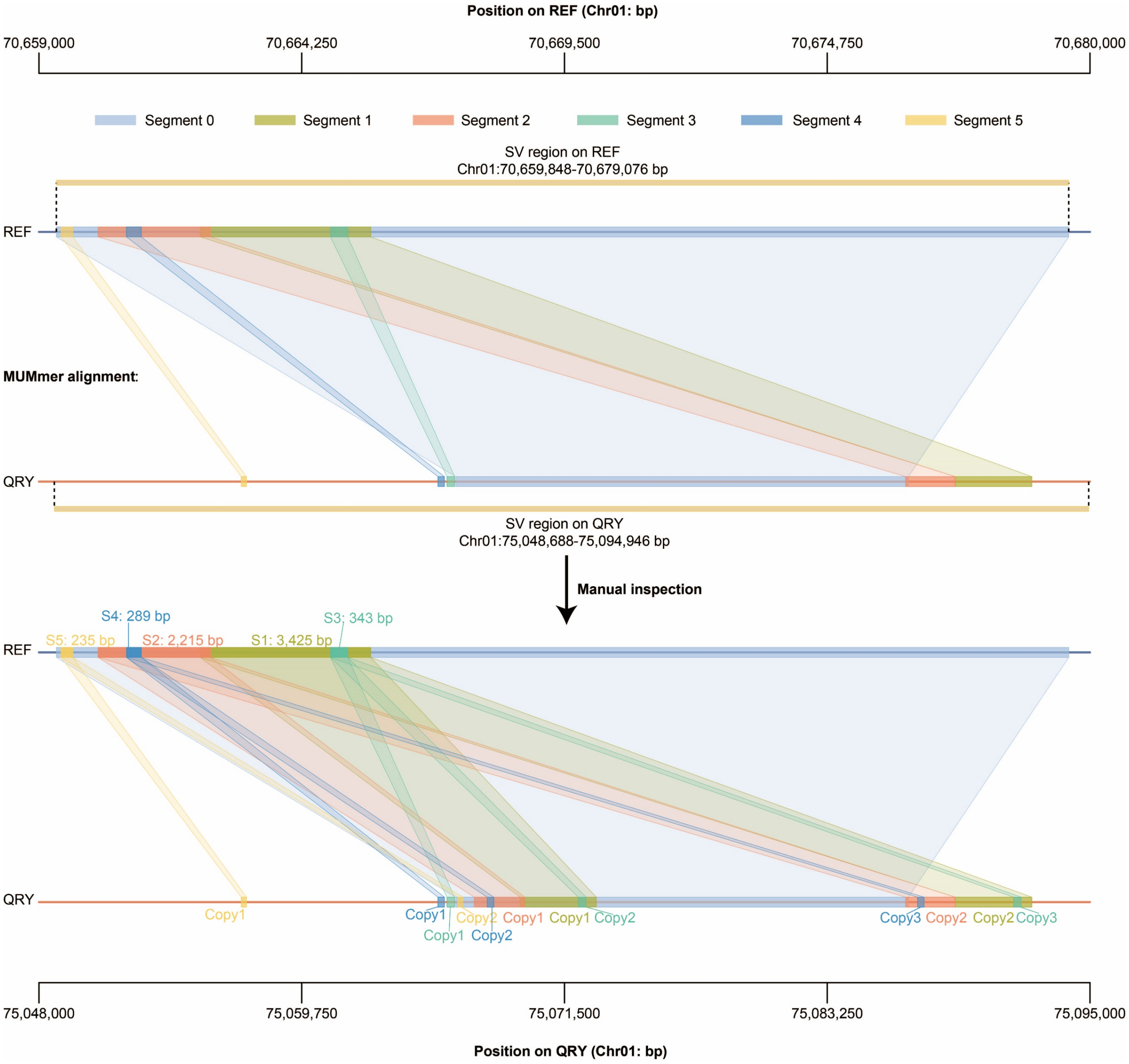

Supplementary Fig. 18 | Analysis of repetitive sequences within SV regions. The complex SV region at 70,659,848 to 70,679,076 bp on Chr01 in the reference genome was aligned to the SV region in the query genome, resulting in six alignment segments reported by MUMmer. The copies of five segments in the query genome, excluding Segment 0, were identified after manual analysis.

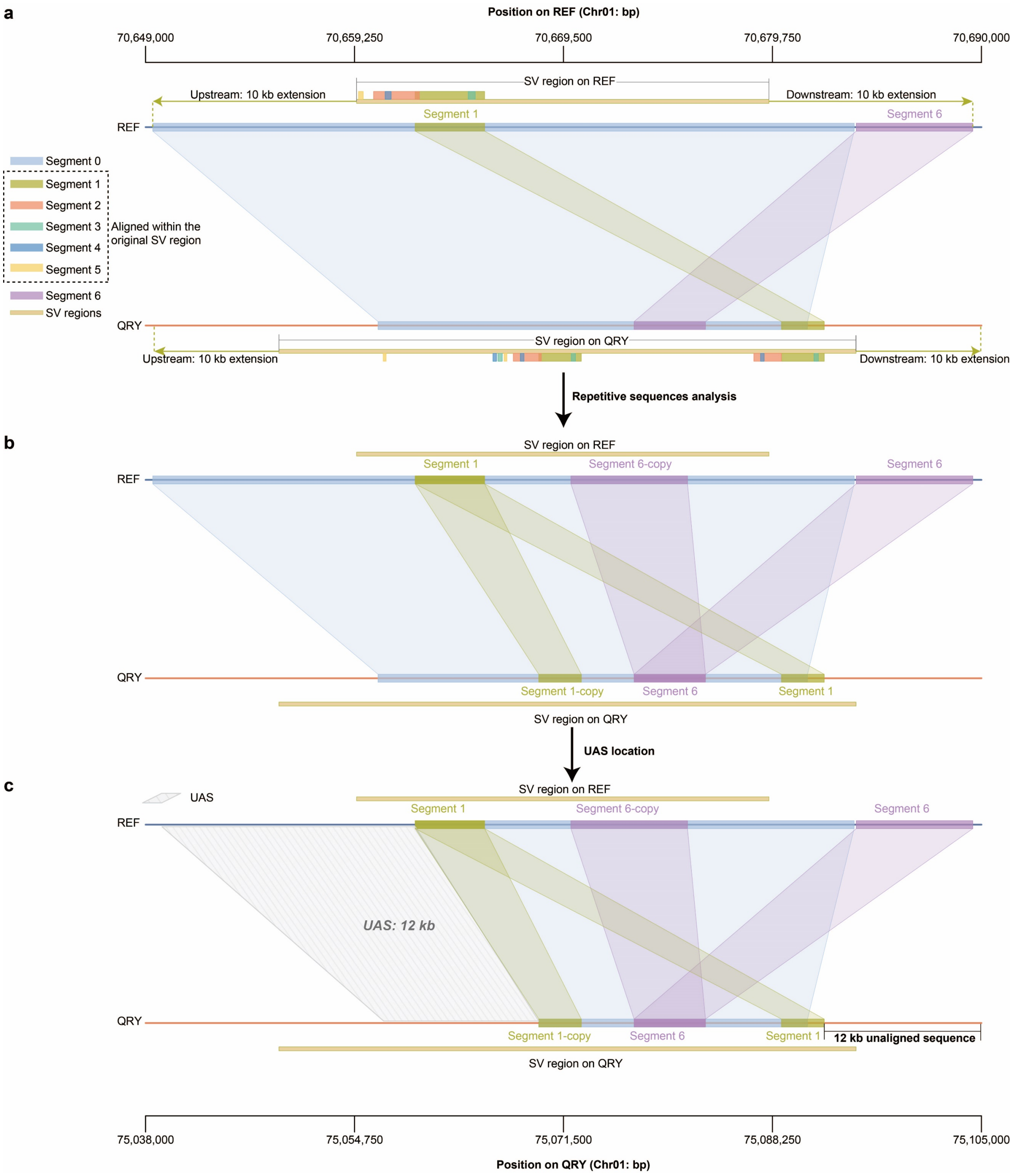

Supplementary Fig. 19 | Analysis of the complex SV region after 10 kb extension. a) Three segments were identified, with segments 2 to 5 (before extension) showing the best alignment. Segment 6 emerged as a new alignment after extending both upstream and downstream. **b)** Identification of multiple copies of the repetitive segments. Segment 1 and Segment 6 exhibit varying copy numbers across both genomes. **c)** Localization of UAS in the upstream region. A 12 kb segment was unaligned to the reference genome, and no UAS was found in the downstream region in the query genome.

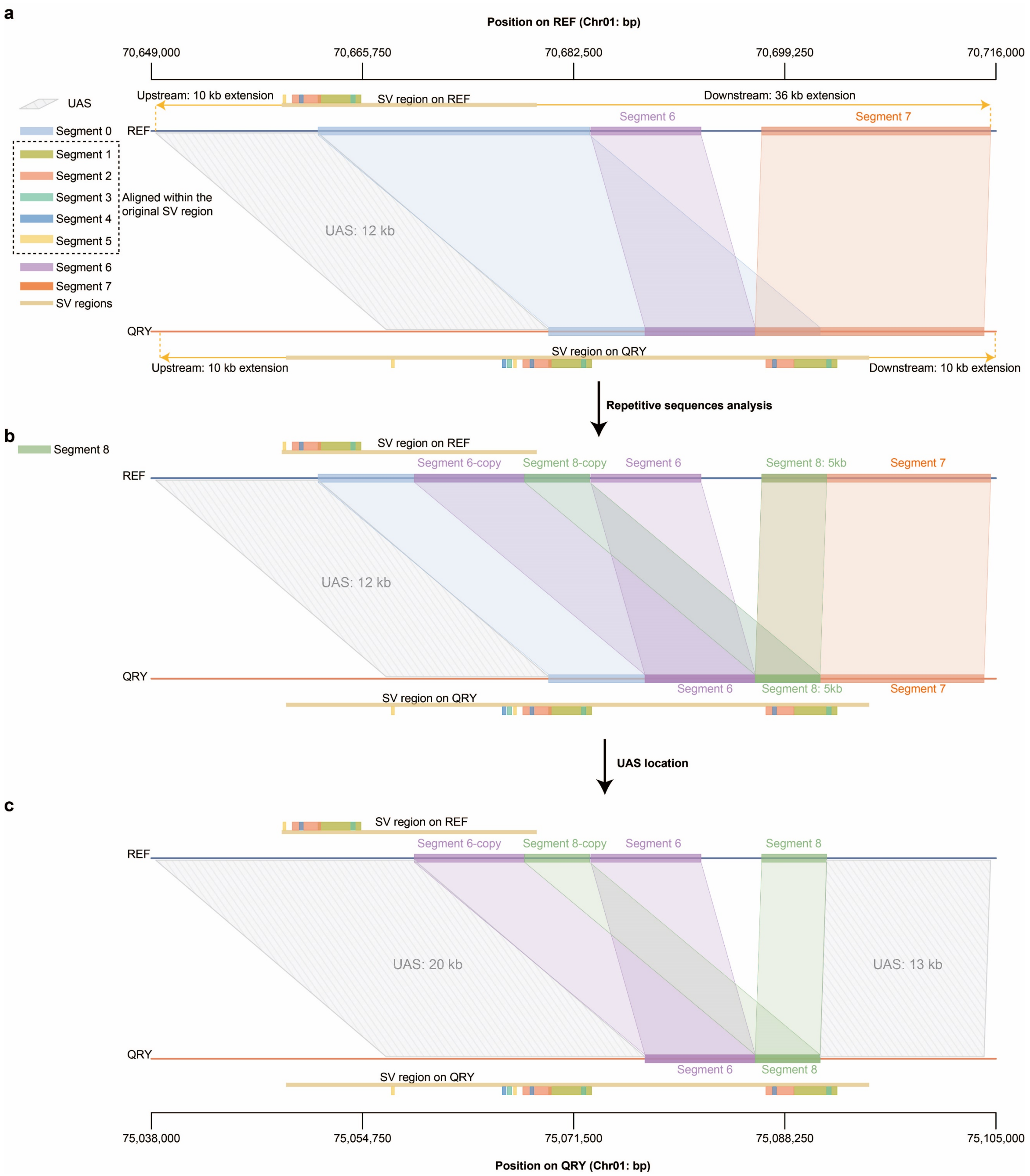

Supplementary Fig. 20 | Localization of downstream UAS in complex SV region at 70,659,848-70,679,076 bp on Chr01. a) Segment 7 emerged as new alignment after extending 10 kb upstream in the query genome and 36 kb downstream in the reference genome. **b)** Identification of multiple copies of the repetitive segments. Two segments (Segment 6 and Segment 8) have one copy in the query genome but two copies in the reference genome. Segment 8 is an overlapped sequence of Segment 6 and Segment 7. **c)** UAS located in the downstream region (13 kb).

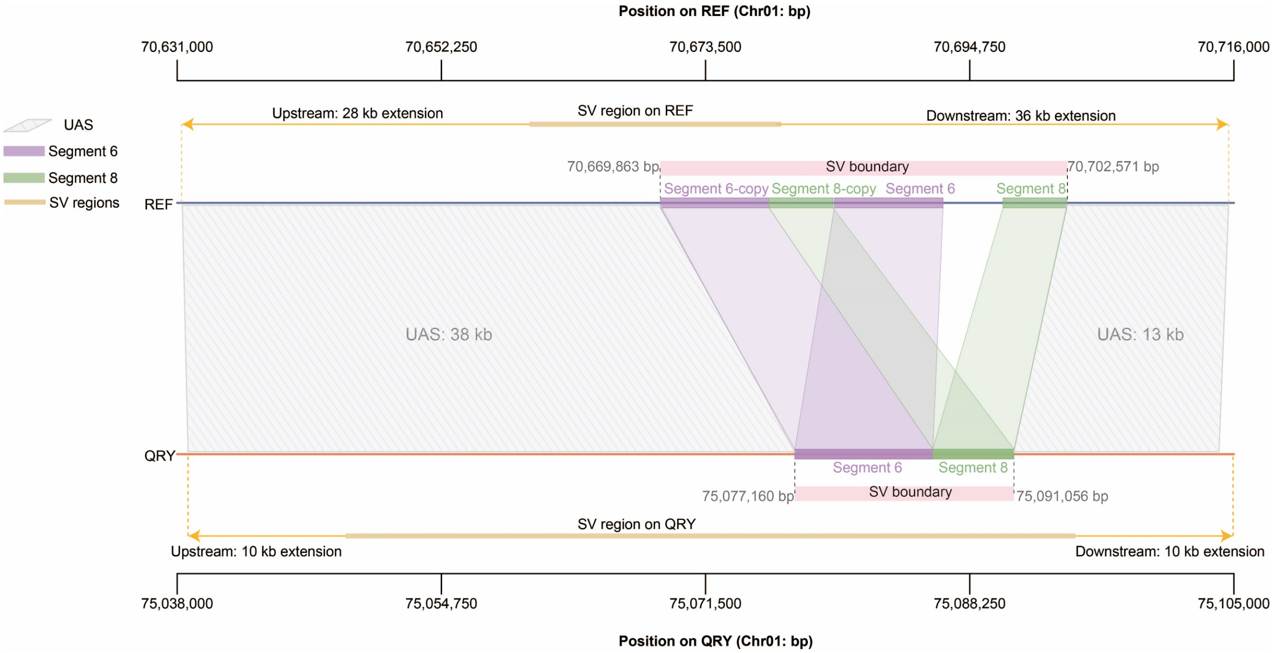

Supplementary Fig. 21 | Determination of SV boundaries in the complex SV region at 70,659,848 to 70,679,076 bp on Chr01.

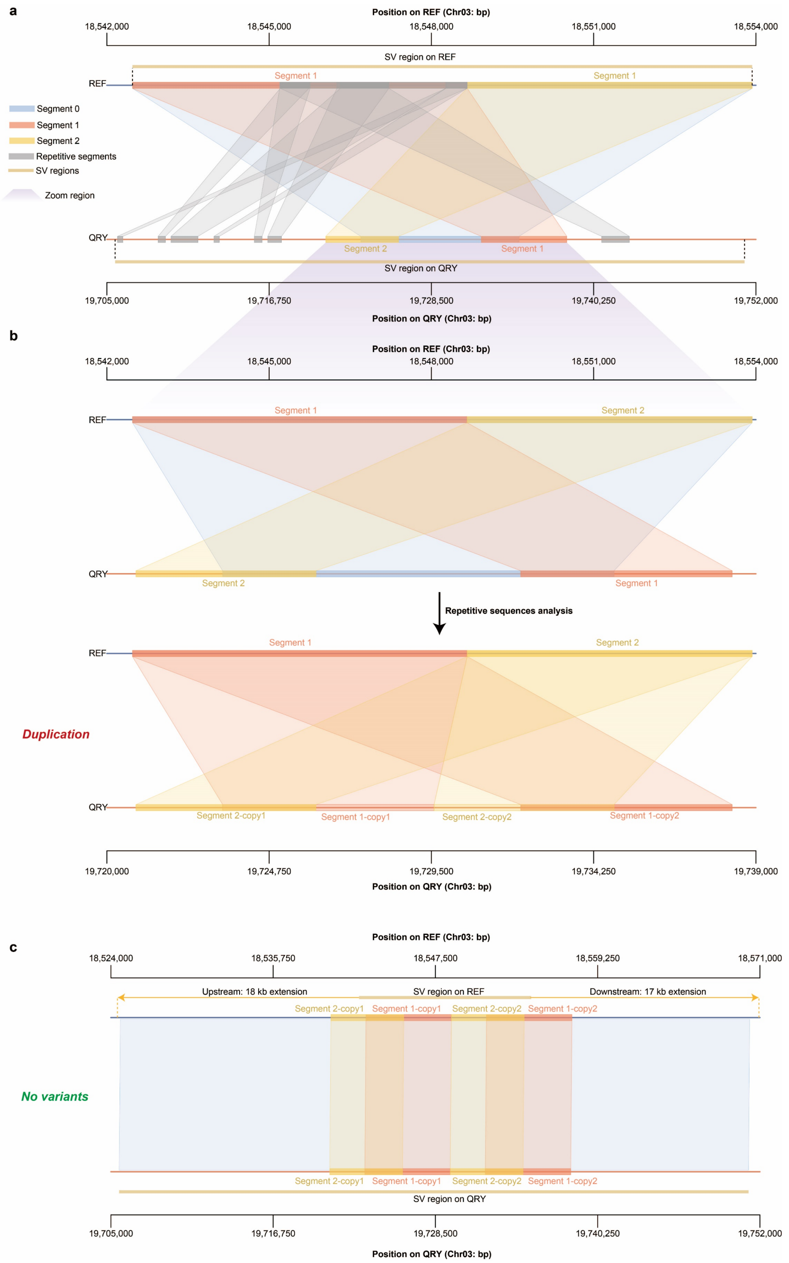

Supplementary Fig. 22 | Missing copies resulting in false positive SV regions. a) Alignments of the SV regions between 18,542,473 and 18,553,931 bp on Chr03 in both genomes. Ten segments are aligned in total. Seven repeat-aligned segments, marked by gray squares, are distributed in the redundant sequences in the query genome. **b)** Segment 1 and Segment 2 each have one copy in the reference genome and two copies in the query genome in the initial alignment, indicating duplication in this region. **c)** Extension flanking regions by 18 kb upstream and 17 kb downstream in the reference genome. Both Segment 1 and Segment 2 have two copies in both genomes, indicating no actual variants.

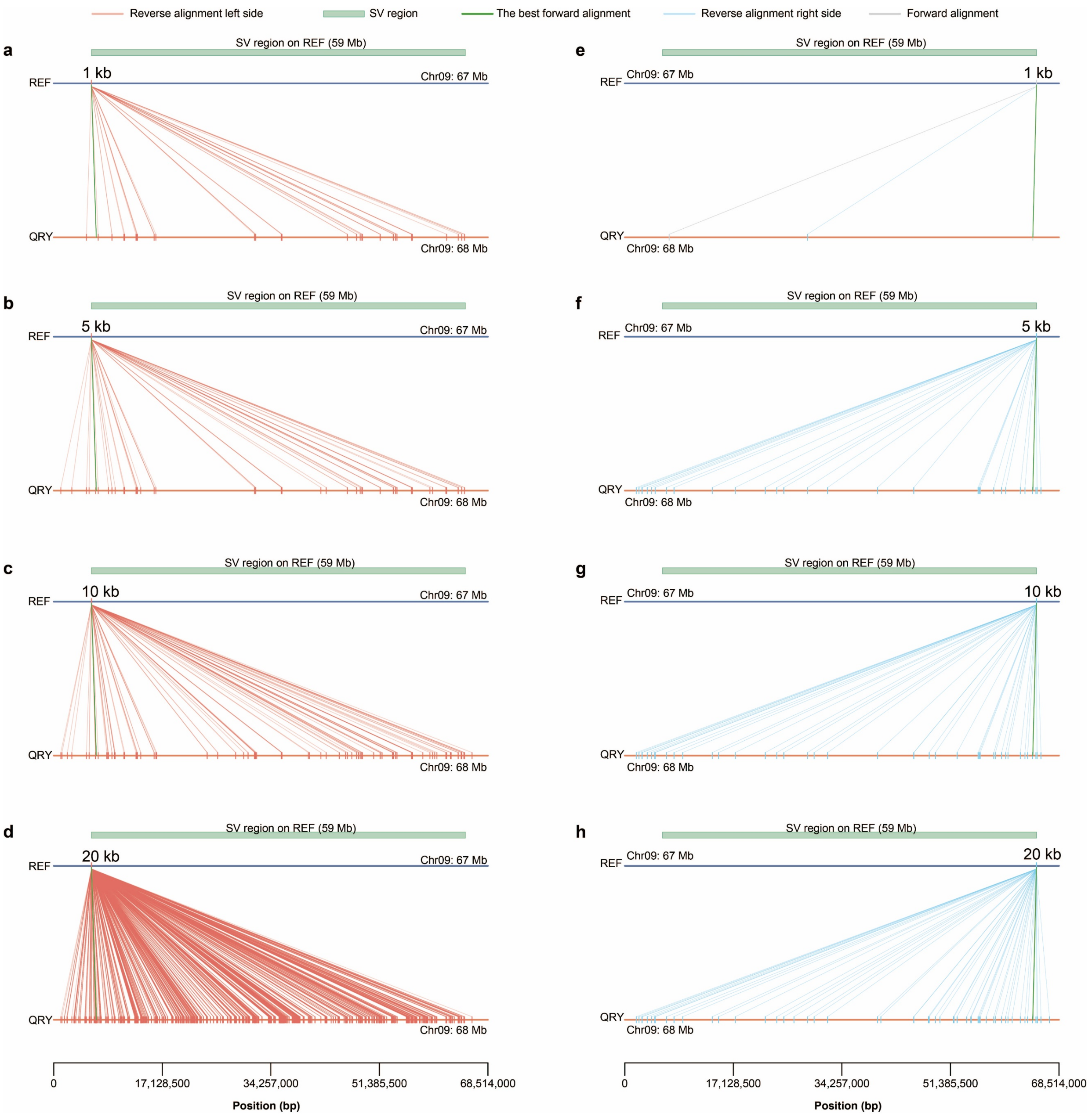

Supplementary Fig. 23 | Reverse-aligned across whole chromosomes causing misdetection of the SV region. a)-d) Segments of 1 kb, 5 kb, 10 kb, and 20 kb from the start of the SV region spanning from 5.94 to 64.97 Mb on Chr09 in the reference genome were aligned to the query genome. The number of reverse-aligned segments increased with the length of the extracted sequences in the reference genome across Chr09 in the query genome. However, the longest and best-aligned segment (green lines) consistently remained forward-aligned at the same position. **e)-h)** Segments of 1 kb, 5 kb, 10 kb, and 20 kb from the end of the SV region spanning from 5.94 to 64.97 Mb on Chr09 in the reference genome were aligned to the query genome.

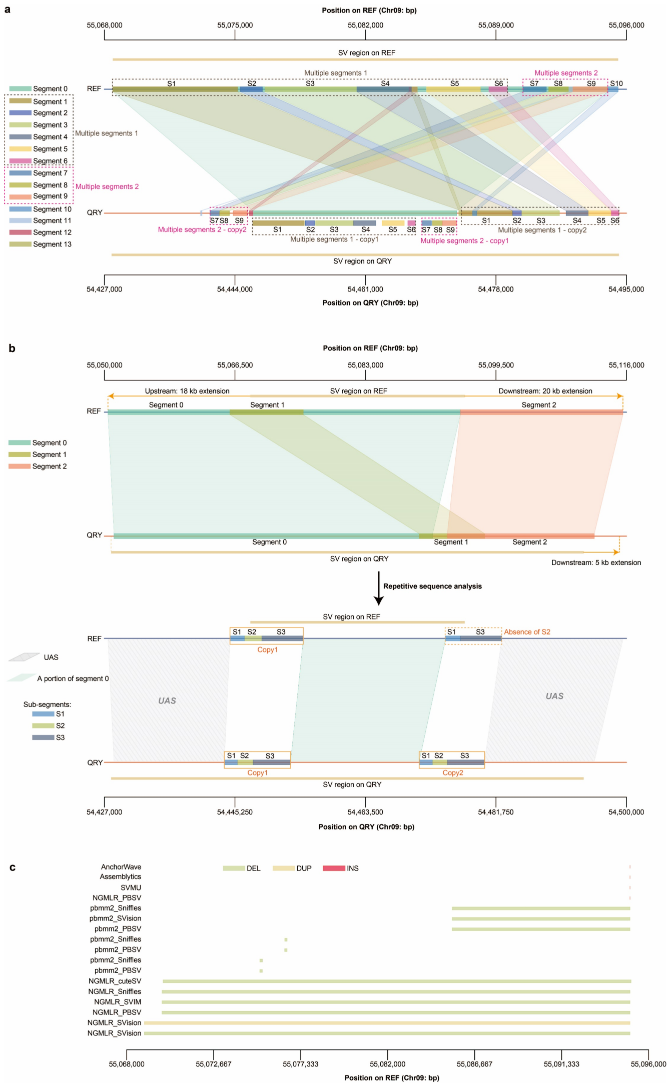

Supplementary Fig. 24 | Complex repetitive sequences causing misalignment of copies. a) Alignment of the SV region at 55,068,947 to 55,095,058 bp on Chr09 in the reference genome reveals the following structure: Segments S1, S2, S3, S4, S5, and S6 form Multiple segments 1 (dark brown dashed rectangle), while segments S7, S8, and S9 form Multiple segments 2 (pink dashed rectangle). These Multiple segments exist as a single copy and sub-segments of S0 in the reference genome but are duplicated in the query genome. **b)** UAS location after extending 18 kb upstream and 20 kb downstream in the reference genome. The alignment indicates that most of the sub-segments found unique alignments. It also shows that copies containing three small segments (S1, S2, and S3) are duplicated in both genomes; however, one copy in the reference genome lacks the small segment S2, causing misalignment. **c)** SV loci detected by multiple callers in this region result in three SV types, showing inconsistent identification.

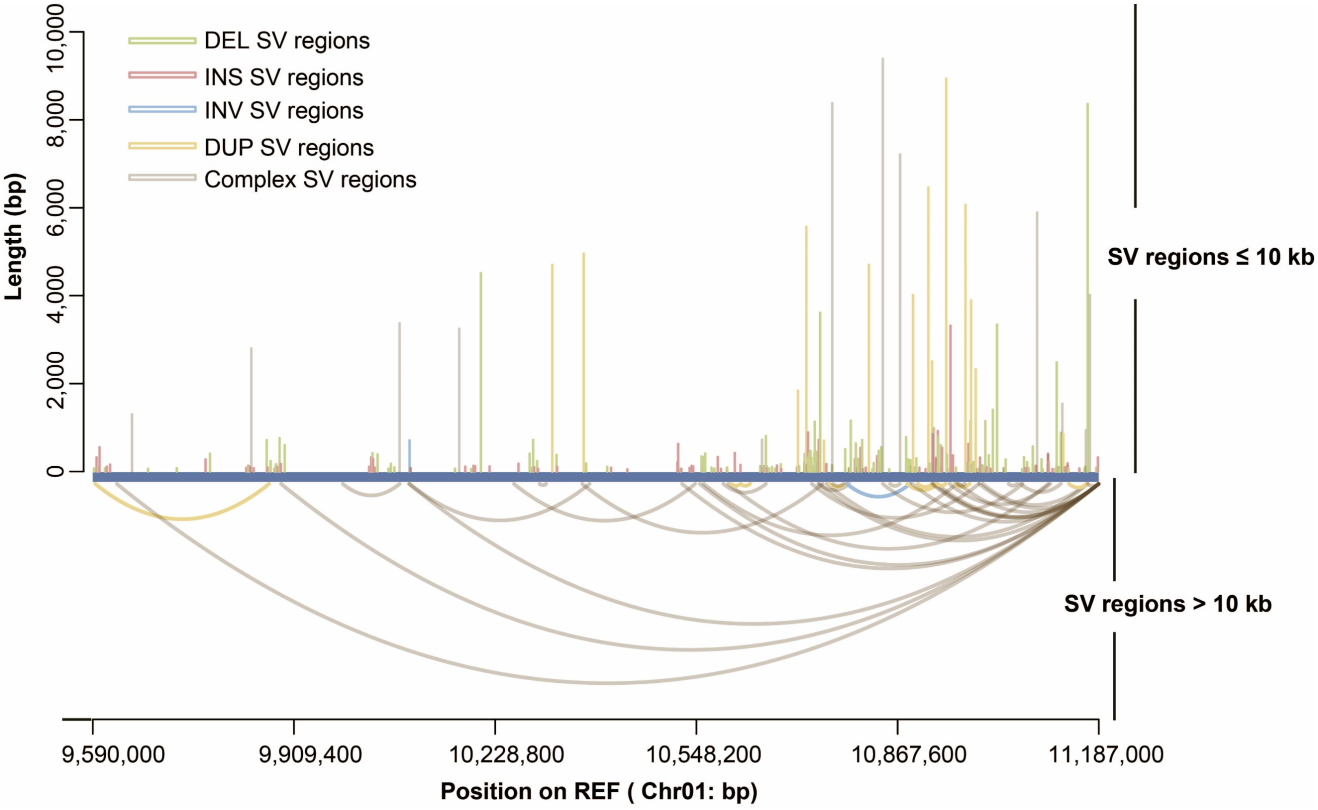

Supplementary Fig. 25 | SV regions detected in tyfonas-like region. A total of 289 SV regions were identified, encompassing multiple types. Among these, numerous complex SV regions are longer than 10 kb.

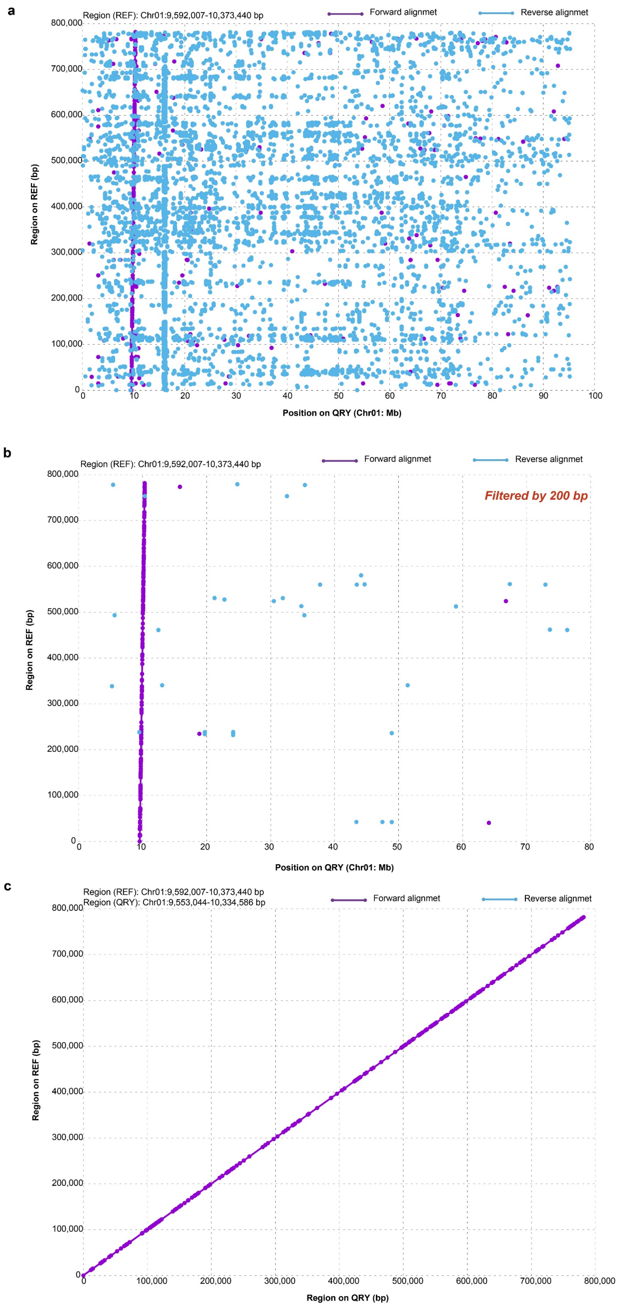

Supplementary Fig. 26 | Manual inspection of 70 SV regions in the tyfonas-like region. a) Alignments of all SV regions in the reference genome to Chr01 in the query genome revealed multiple segments in both forward (purple lines) and reverse (blue lines) orientations using MUMmer. **b)** After filtering out alignments smaller than 200 bp, only continuous forward-aligned segments were observed, indicating no SVs were detected in these 70 SV regions. **c)** The segment from 9,592,007 to 10,373,440 bp on Chr01 in the reference genome (from these 70 SV regions) uniquely forward-aligned with 9,553,044 to 10,334,586 bp in the query genome.

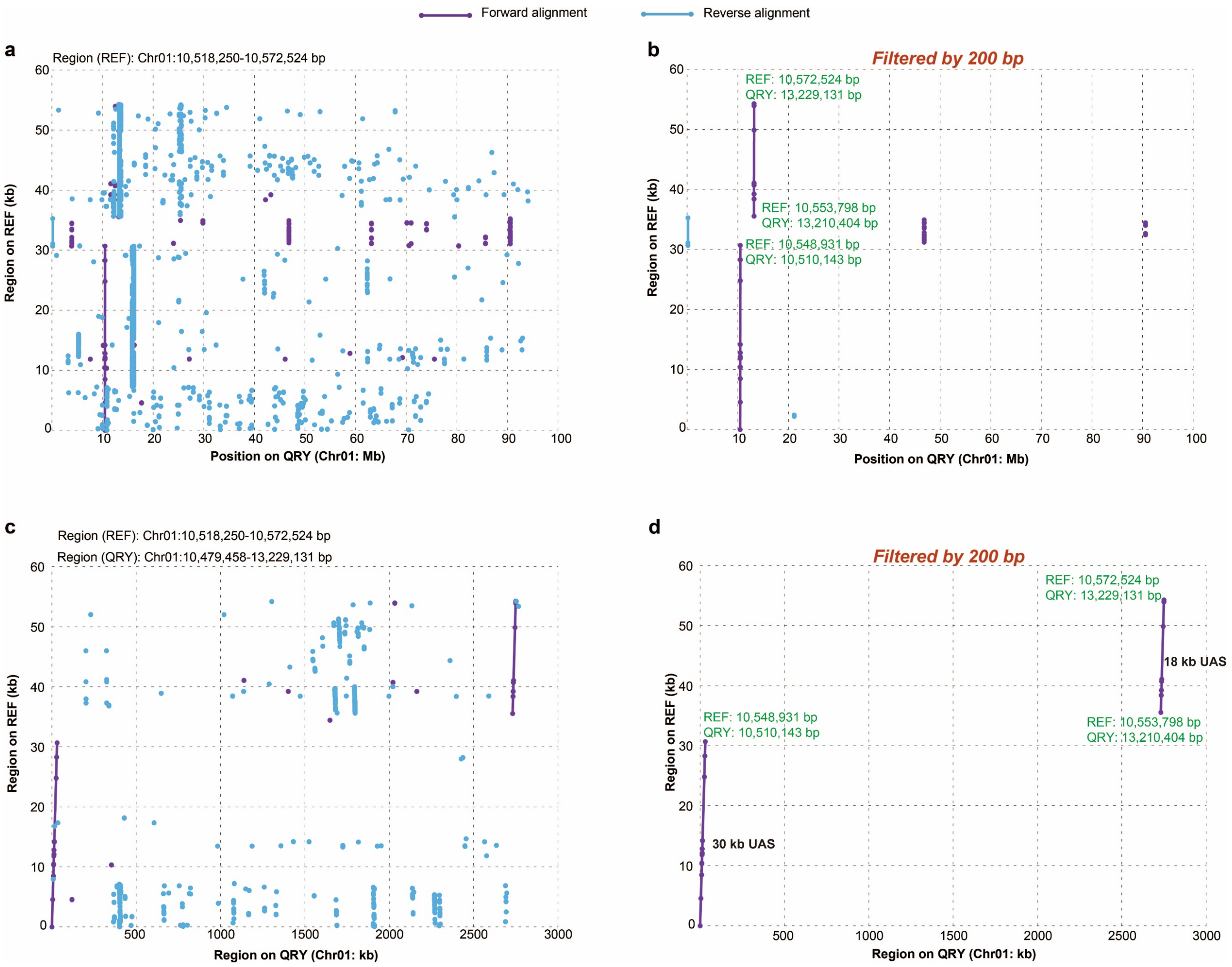

Supplementary Fig. 27 | Manual inspection of SV regions at 10.51 to 10.57 Mb in the tyfonas-like region. a) SV regions in the reference genome aligned to entire Chr01 in the query genome. **b)** Filtering segments shorter than 200 bp. **c)** SV regions in the reference genome aligned to 10,479,458 to 13,229,131 bp on Chr01 in the query genome. **d)** Filtering segments shorter than 200 bp revealed a large insertion (2.7 Mb) in the query genome.

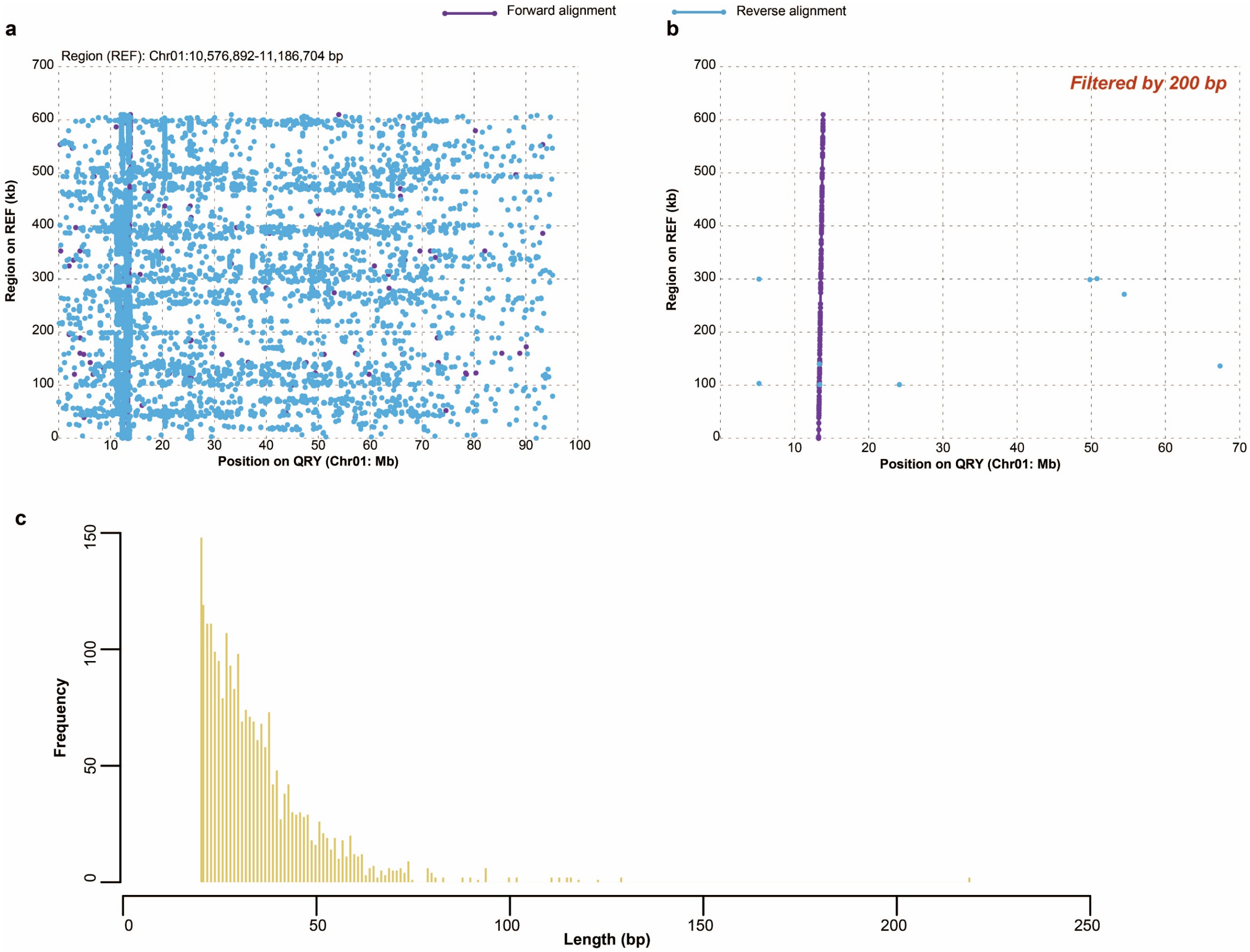

Supplementary Fig. 28 | Manual inspection of the remaining 201 SV regions in the tyfonas-like region. a) SV regions in the reference genome aligned to the entire Chr01 in the query genome revealed whispered small reverse-aligned segments. **b)** Filtering segments shorter than 200 bp showed that most reverse-aligned segments were repetitive sequences. These regions are aligned in both genomes in forward orientation without SVs. **c)** The length distribution of reverse-aligned segments.

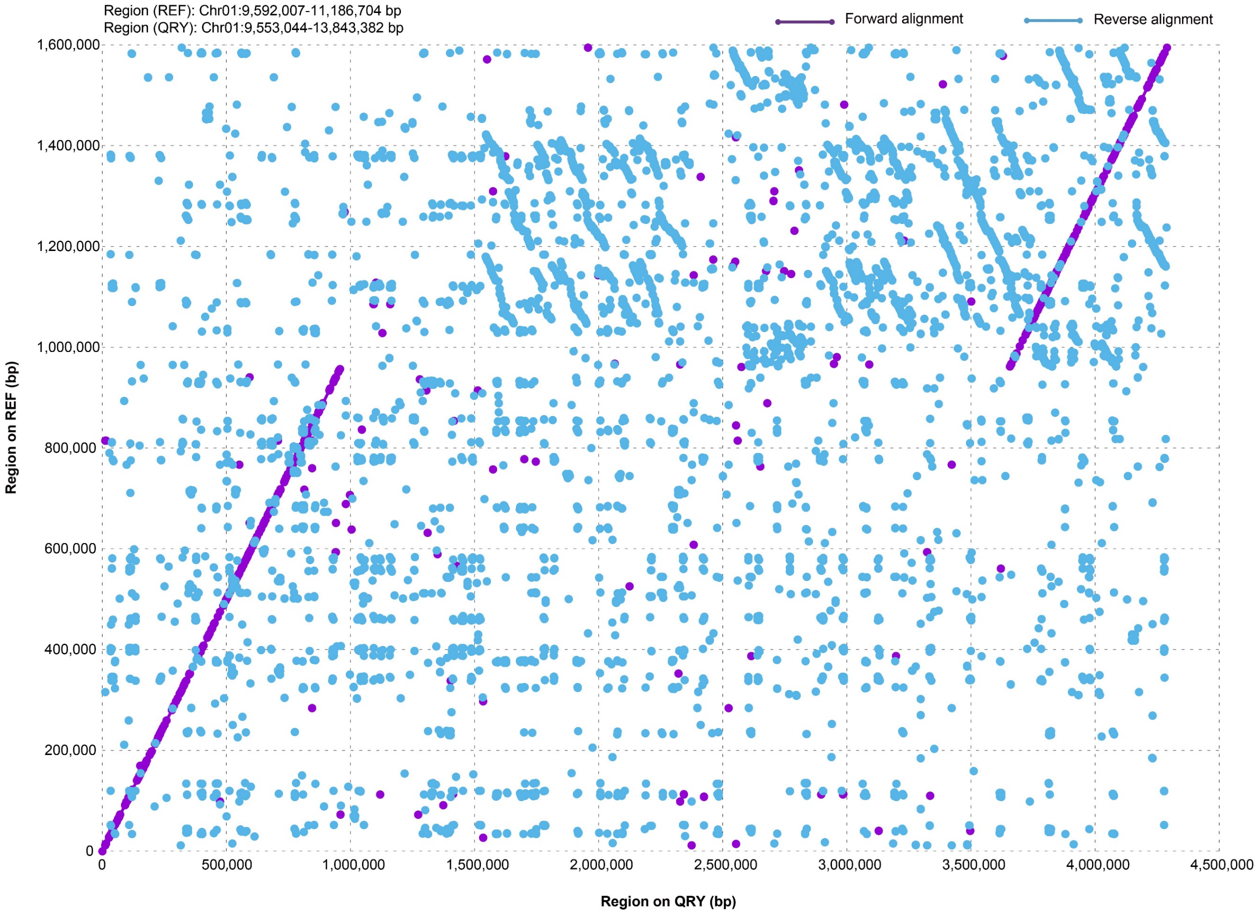

Supplementary Fig. 29 | Alignments of tyfonas-like region in both genomes.

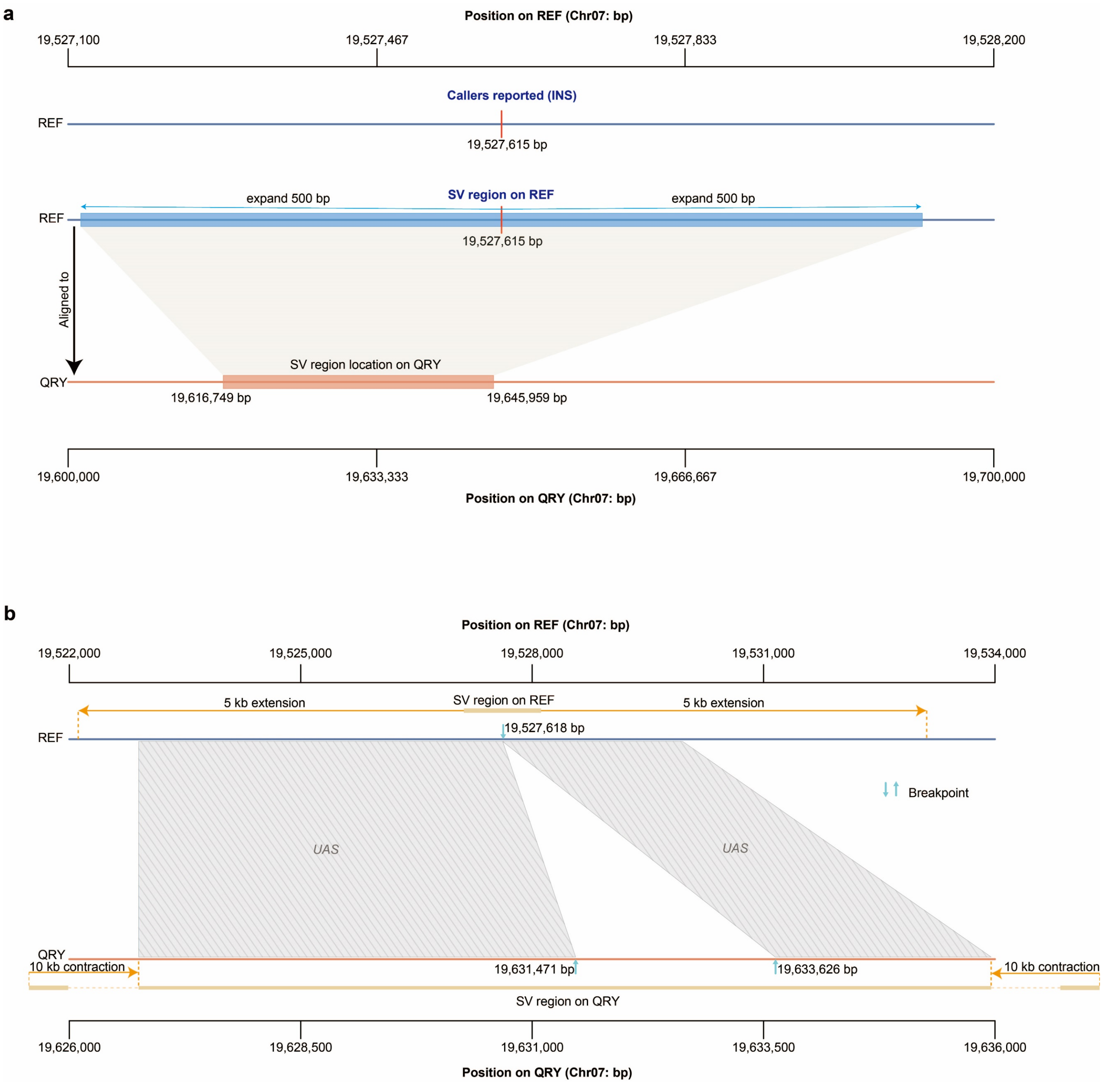

Supplementary Fig. 30 | SV breakpoints identification in the simple region. a) For the SV region at 19,527,615 bp on Chr07 in the reference genome, non-repetitive segments aligned to query genome. **b)** Localization of UAS after contracting 10 kb both upstream and downstream in the query genome, thereby SV boundaries was identified as breakpoints.

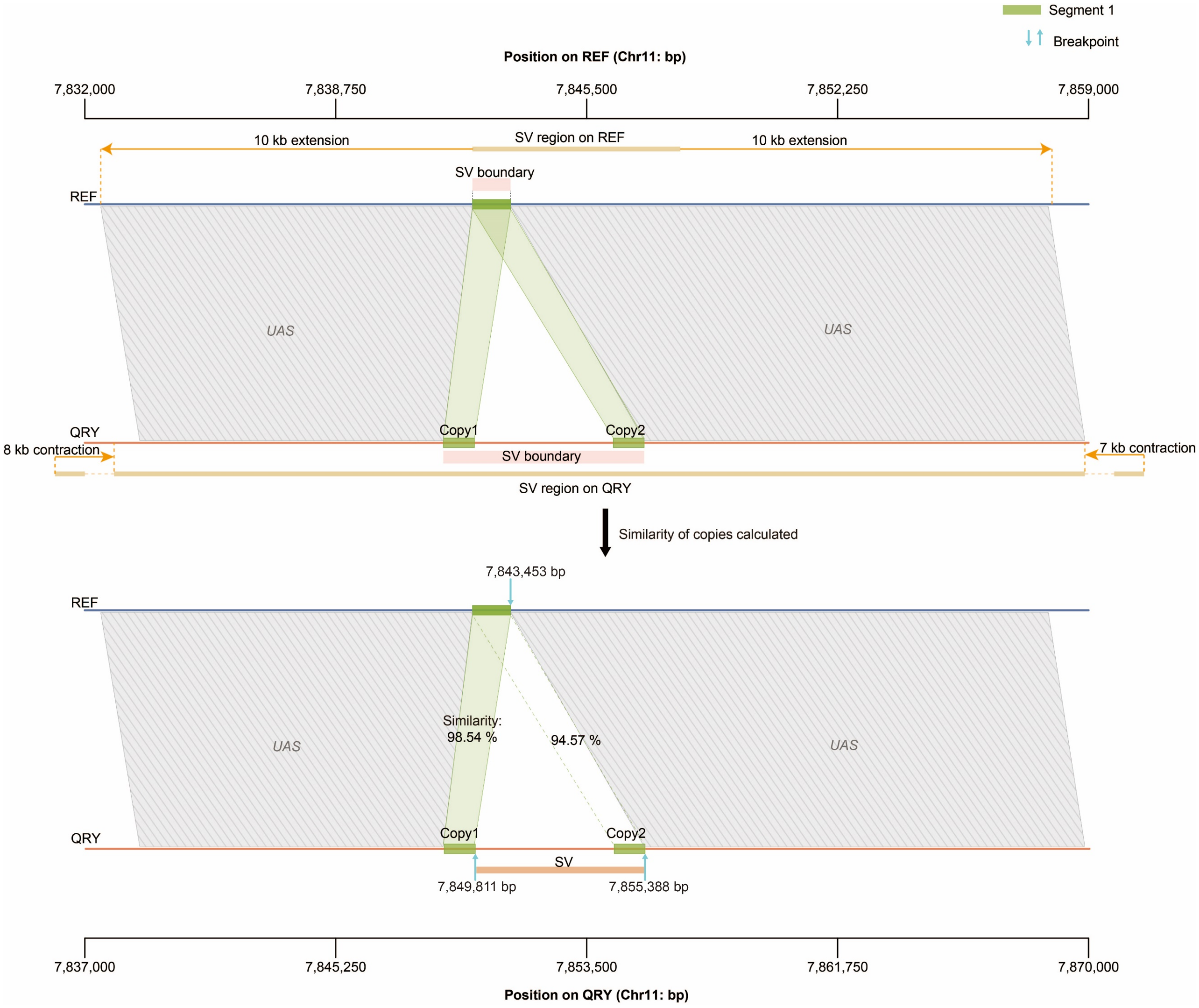

Supplementary Fig. 31 | SV breakpoints identification of copies with one segment. For the SV region spanning from 7,842,427 to 7,848,018 bp on Chr11, the reference genome contains one copy with a segment (depicted as green rectangles), whereas the query genome harbors two copies (labeled as Copy1 and Copy2). Following similarity calculations between the copies, the more similar copy (Copy1) was selected. Additionally, an insertion (depicted as pink rectangles) was detected between the end of Copy1 and Copy2 in the query genome.

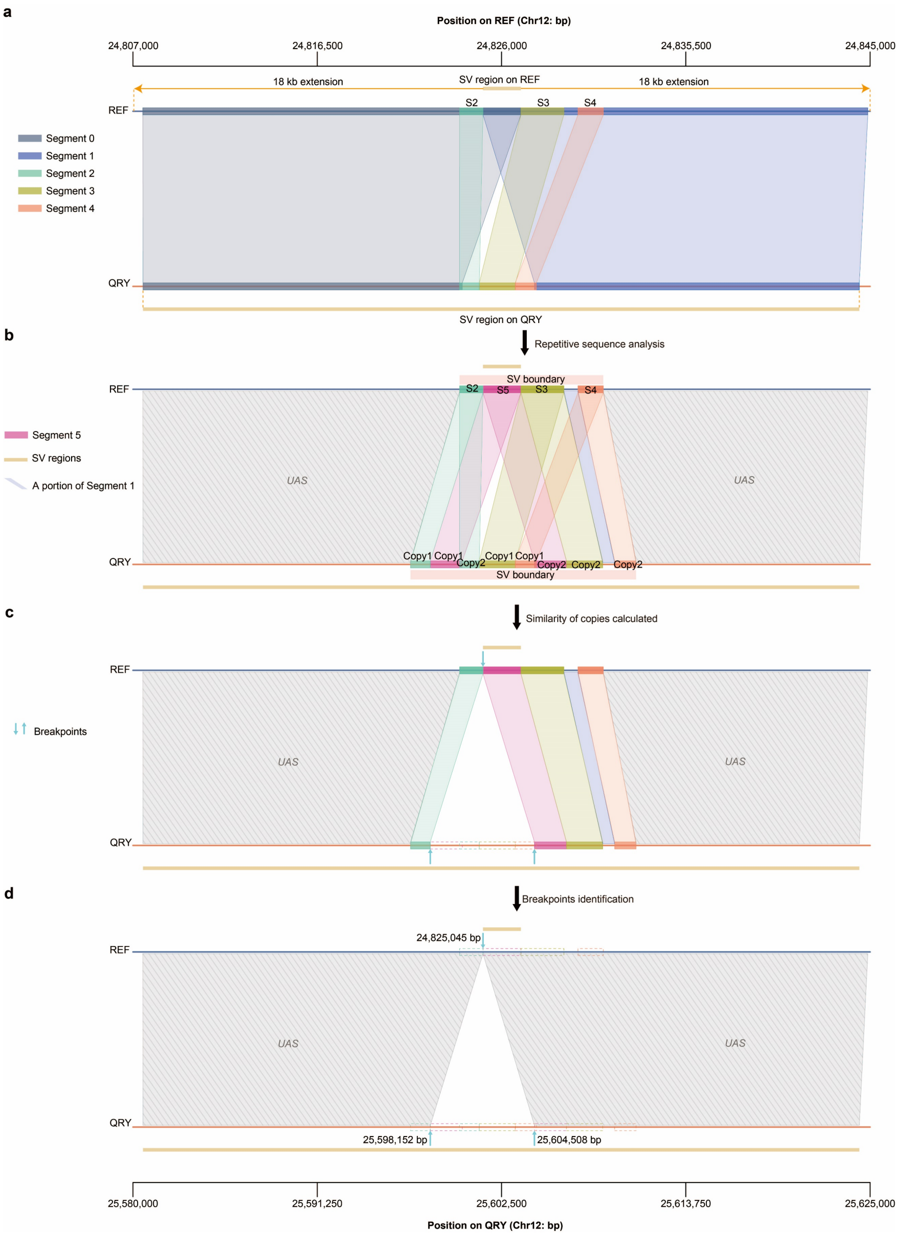

Supplementary Fig. 32 | SV breakpoints identification in copies with multiple segments. a) Alignment of SV regions on Chr12 in both genomes after an 18 kb extension upstream and downstream in the reference genome. **b)** Analysis of repetitive sequences in this SV region with clearly defined SV boundaries. Two copies with four segments are present in the query genome, whereas the reference genome contains one copy. **c)** By calculating the similarity of copies, one copy of each of the four segments in the query genome was identified as variant. **d)** A base-pair resolution SV breakpoint (blue arrows) was identified. The SV is positioned from 25,598,152 to 25,604,508 bp.

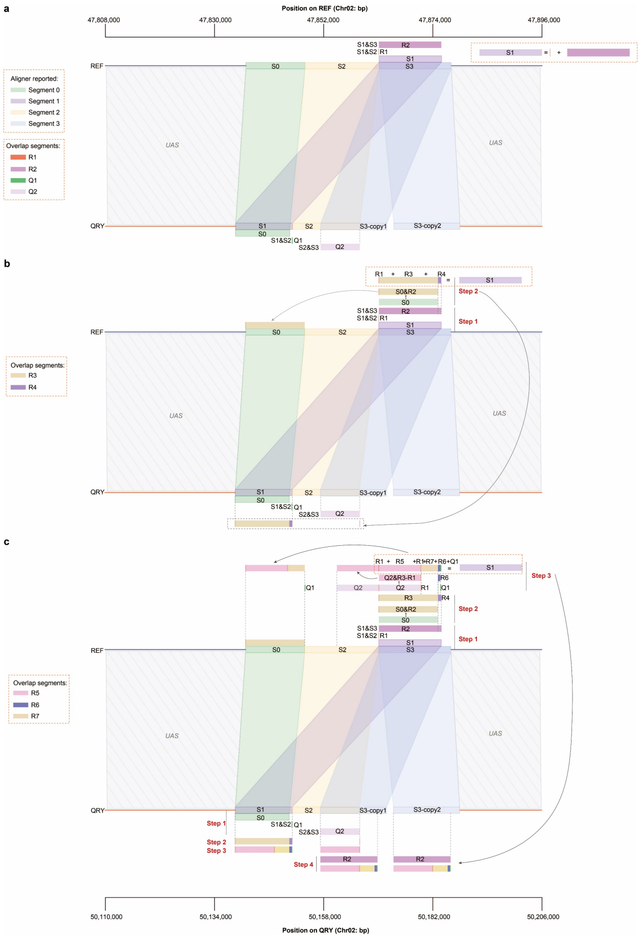

Supplementary Fig. 33 | Analysis of repetitive sequences in highly complex regions. a) In the SV region spanning 47,845,873 to 47,880,655 bp on Chr02, four overlapped segments (S1, S2, S3, and S4) are aligned in both genomes. S1 (purple rectangles) was initially analyzed as it overlaps with all other segments. During the first analysis step, S1 overlaps with S2 and S3 in the reference genome, causing it to split into two sub-segments (R1 and R2). Meanwhile, three overlapping segments (S0, Q1, and Q2) are identified in the query genome. **b)** Second step in resolving overlapped segments: Sub-segment R2 splits into R3 and R4 due to its overlap with S0 in the reference genome. As a result, S1 now consists of R1, R3, and R4, while S0 comprises R1 and R3. Additionally, R1 is also found in Q2 in the query genome. **c)** Third step in resolving overlapped segments: Q1 and Q2 each have two copies in the reference genome. One copy of Q2 overlaps with R3, splitting it into three sub-segments (R5, R1, and R7). Additionally, one copy of Q1 is entirely contained within R4, dividing R4 into Q1 and R6. Therefore, S1 consists of six sub-segments: two R1, one R5, one R7, one R6, and one Q1. These sub-segments also form other segments across both genomes.

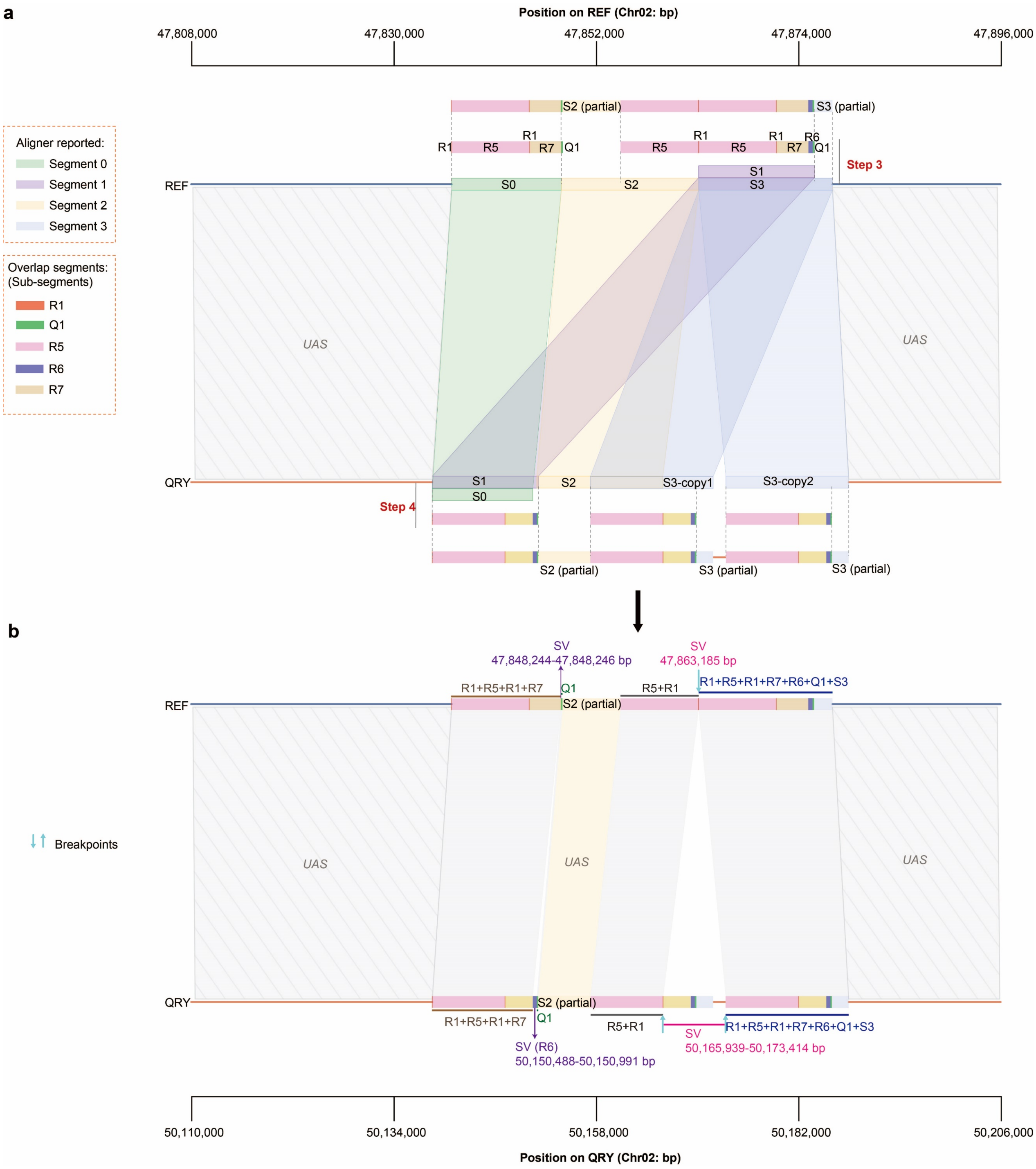

Supplementary Fig. 34 | SV breakpoints identification in highly repetitive regions. a) An SV region on Chr02 contains multiple copies with seven sub-segments (R1, R5, R6, R7, Q1, S2-partial, and S3-partial) in both genomes. **b)** Breakpoint identification relied on the similarity of these sub-segments, revealing two SVs detected in this complex region.

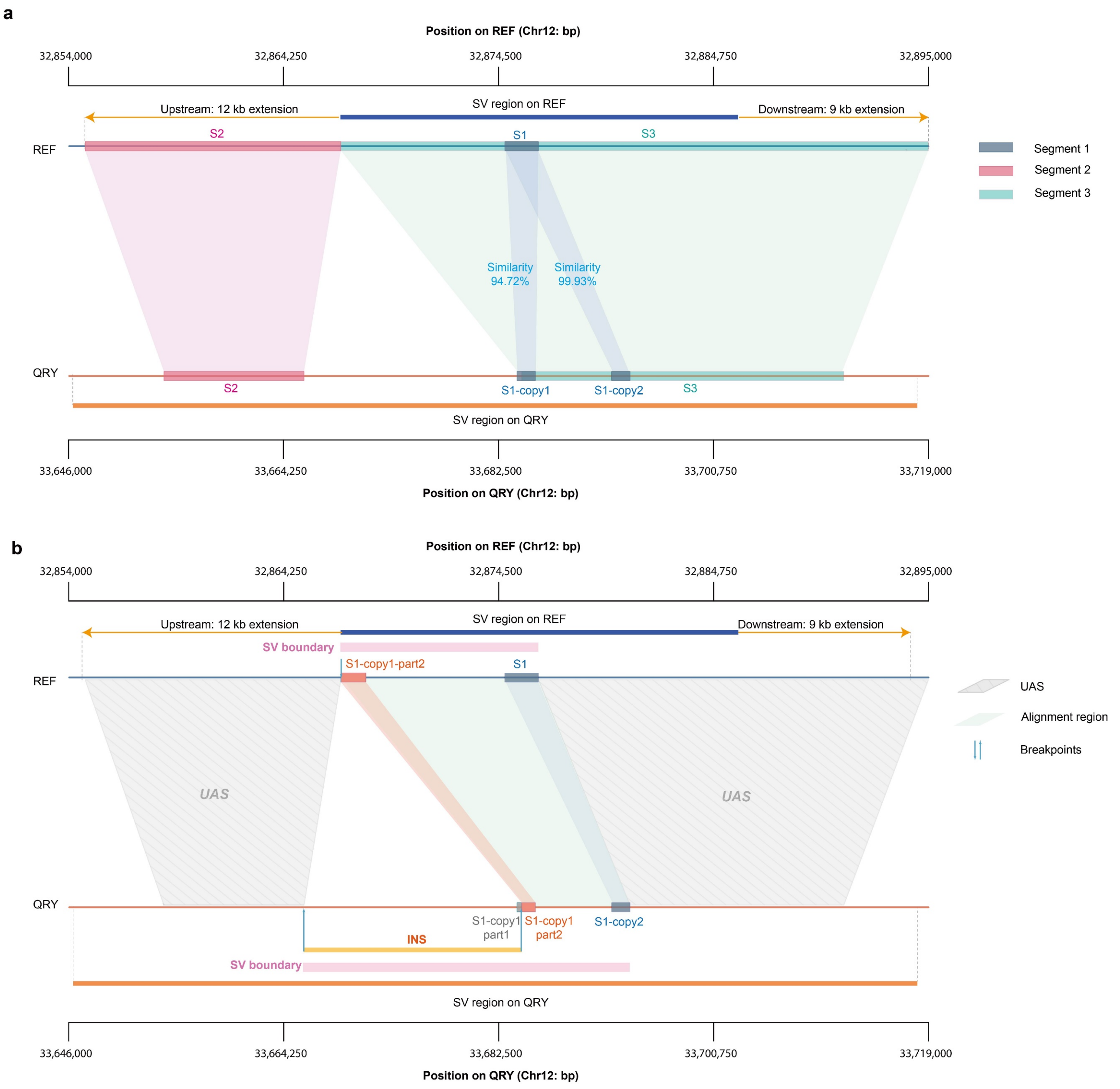

Supplementary Fig. 35 | INS detected in interspersed repetitive sequences. a) One segment (S1) is present in the reference genome, whereas two copies (S1-copy1 and S1-copy2) are observed in the query genome within an SV region on Chr12. **b)** The SV consists of unique sequences and a partial S1-copy1 in the query genome, defined as an INS.

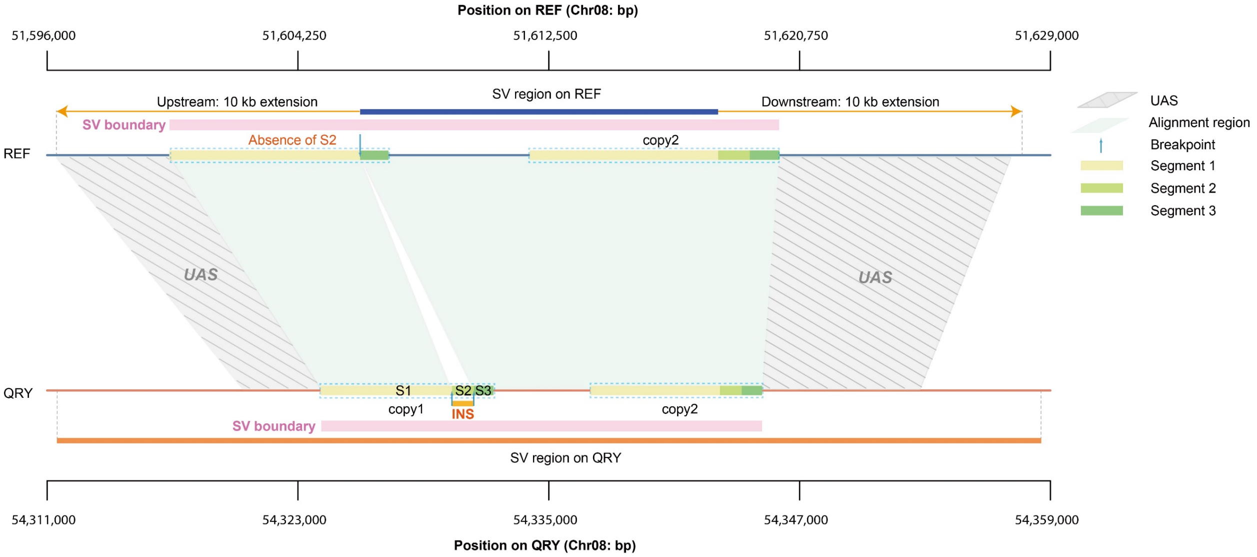

Supplementary Fig. 36 | INS influenced by copies with multiple segments. Two copies exhibit three closely junctional segments (S1, S2, and S3) in both genomes. However, one copy lacks segment S2 in the reference genome, resulting in an INS.

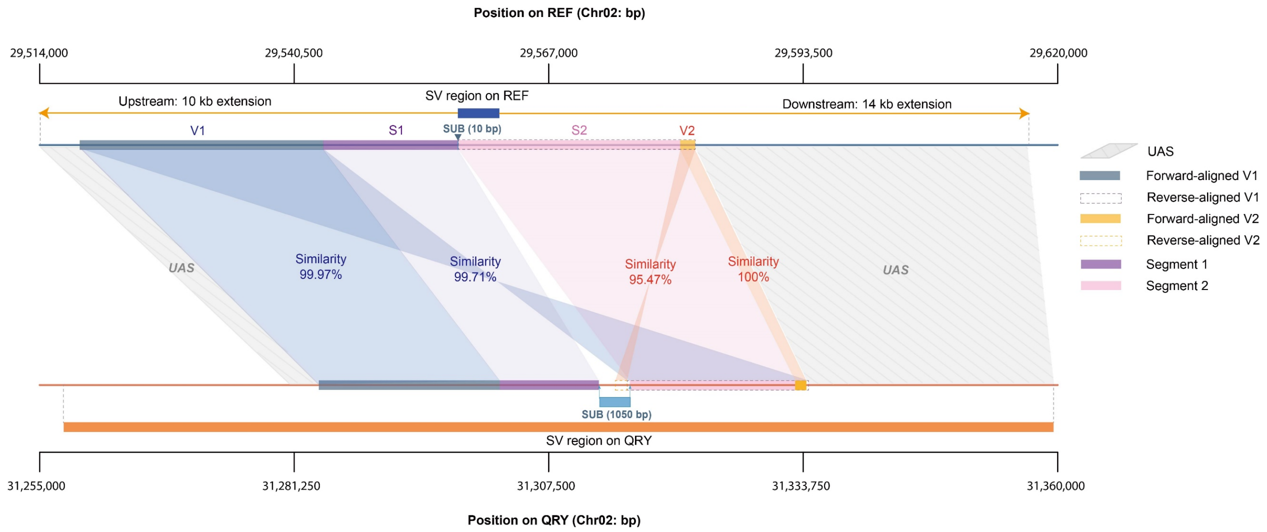

Supplementary Fig. 37 | Challenges in classifying reverse-aligned segments. In an SV region from 29,595,023 to 29,610,742 bp on Chr02, the segments V1 and V2 in the reference genome each have two copies with forward and reverse alignments in the query genome. However, the copies in reverse orientation were excluded based on calculated similarity, resulting in the identification of an INS.

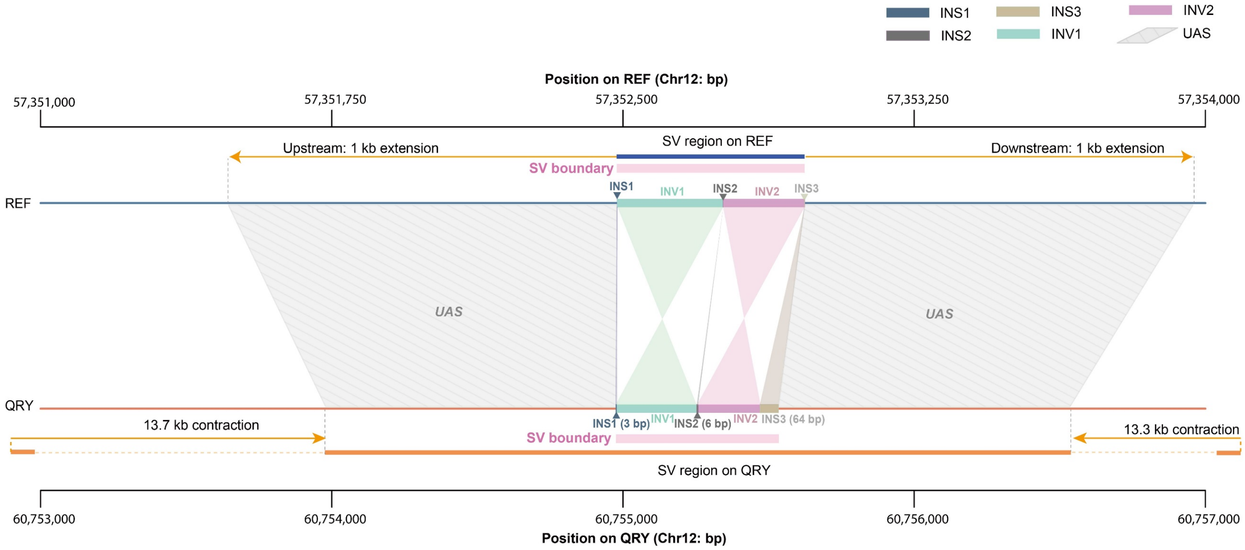

Supplementary Fig. 38 | Clustering of multiple SVs around INVs. The SV region from 57,352,484 to 57,352,970 bp on Chr12 contains five consecutive SVs, including INS1, INV1, INS2, INV2, and INS3.

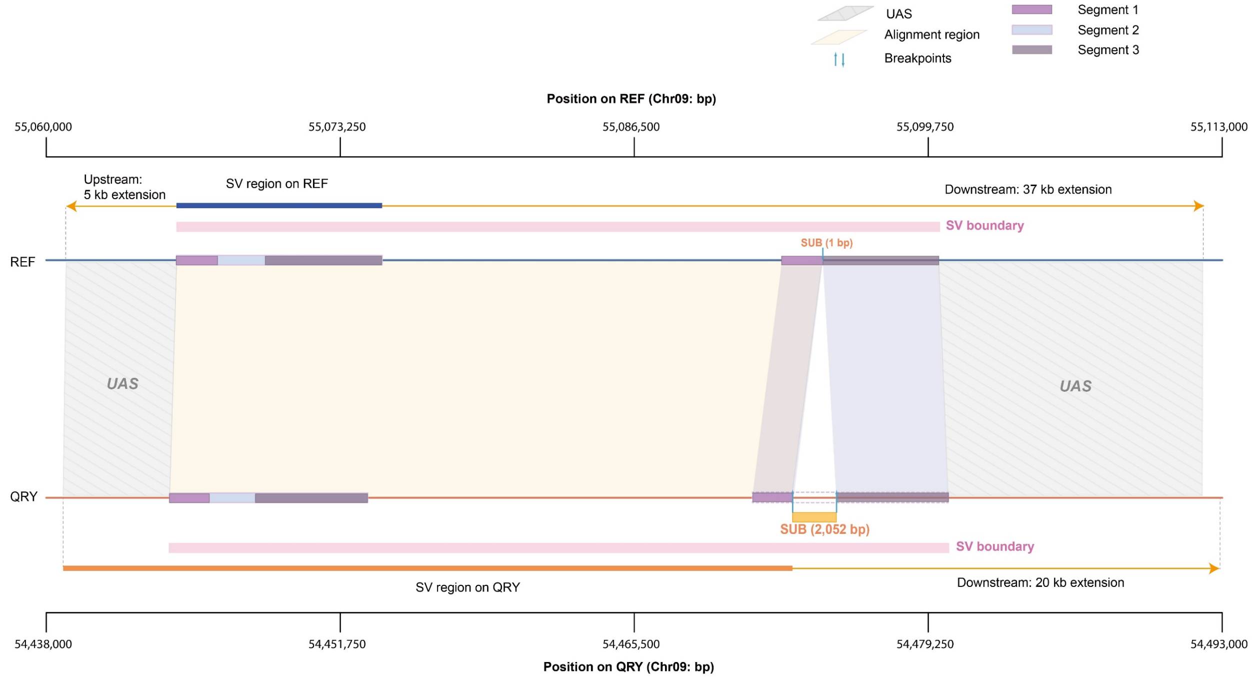

Supplementary Fig. 39 | A single base-pair in one genome for a SUB. A SUB detected in an SV region on Chr09 with a 1 bp variant in the reference genome and a 2,052 bp variant in the query genome.

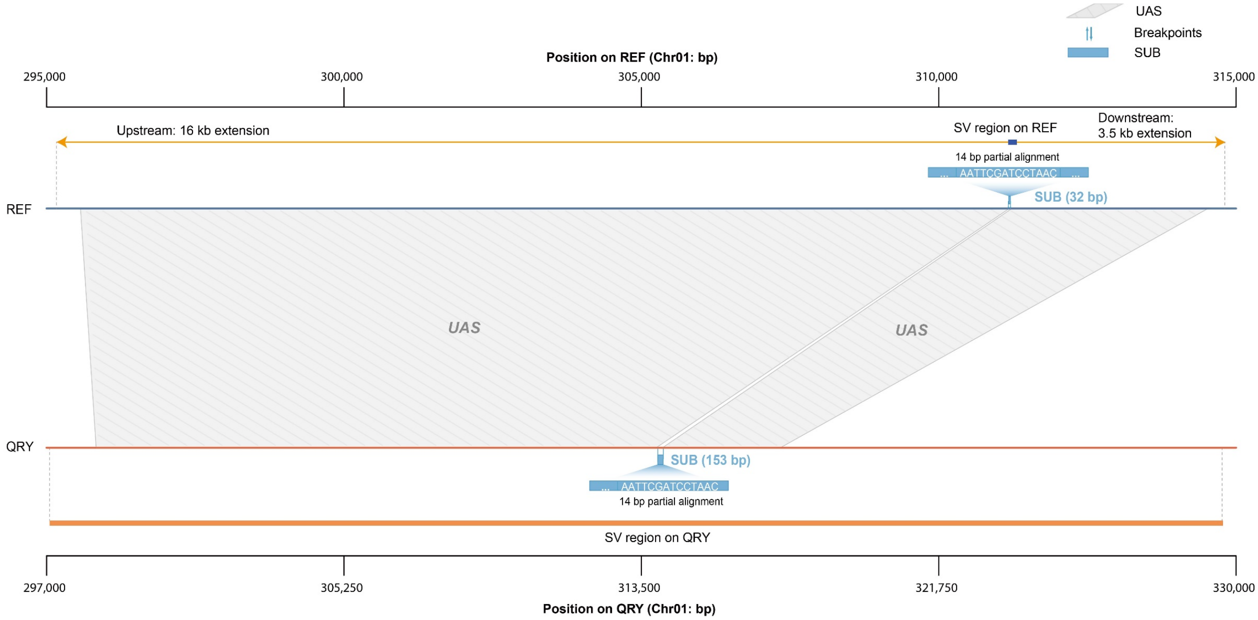

Supplementary Fig. 40 | Partial alignments of a SUB. A SUB with length of 32 bp in the reference genome and 153 bp in the query genome, with 14 bp of the sequences aligning.

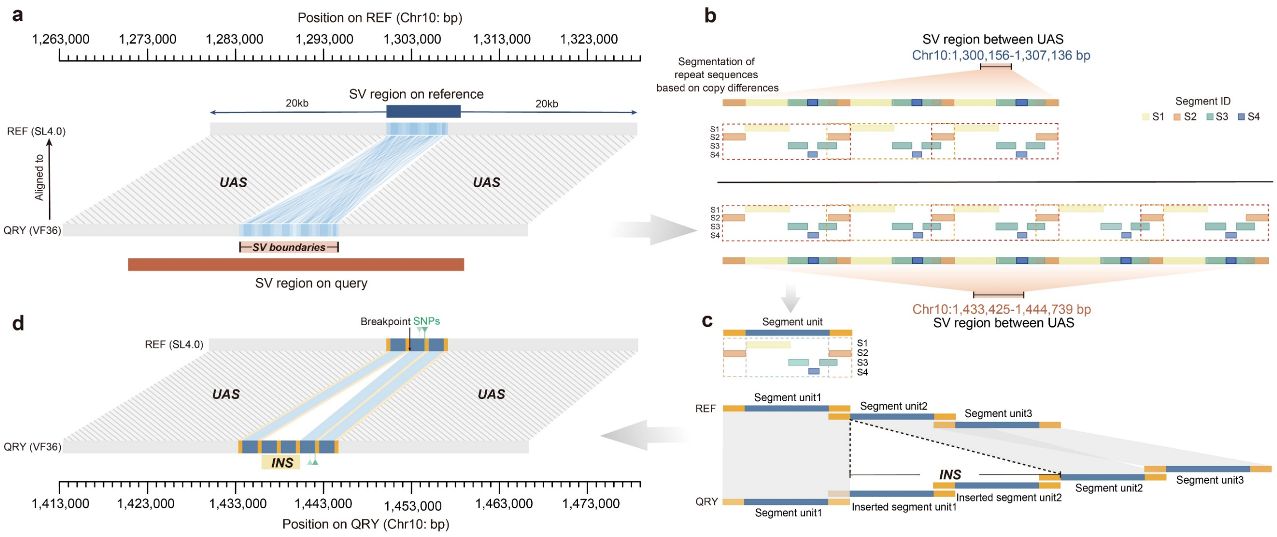

Supplementary Fig. 41 | SV identification in TR regions. a) Numerous overlapped segments aligned between upstream and downstream UAS within an SV region on Chr10. **b)** Analysis of all overlapped segments reveals repetitive sequences in this SV region consisting of multiple copies with four distinct segments (S1, S2, S3, and S4) across both genomes. **c)** The segment unit comprises one S1, two S2, two S3, and one S4, with varying numbers in the two genomes. S2 serves as the overlapped sequence between segment units. Two segment units were identified as an insert variant in the query genome based on similarity calculation. **d)** An INS (yellow rectangle) and five SNPs (green triangle) were detected in this SV region.

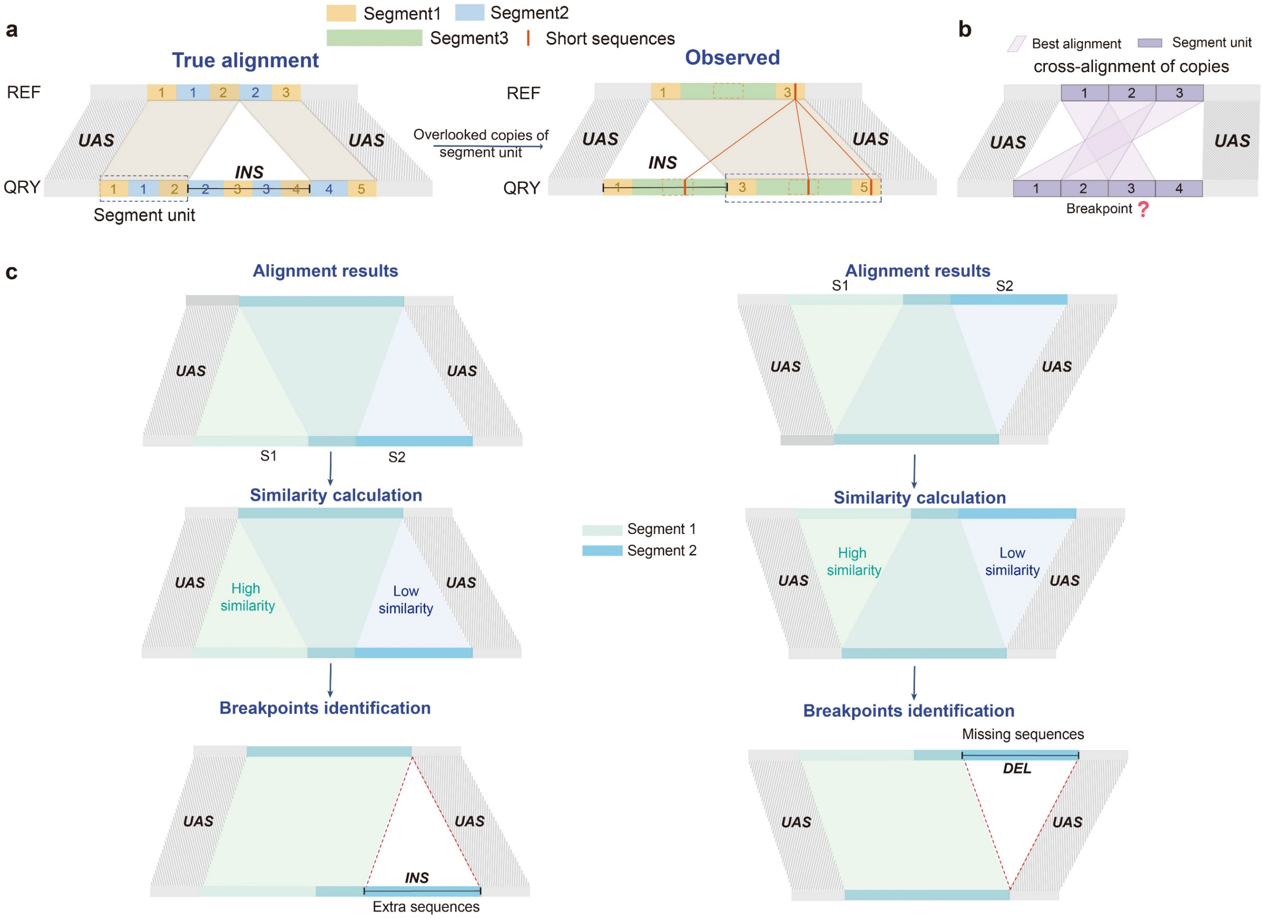

Supplementary Fig. 42 | Challenges and strategy for SV detection in TR regions. a) Misidentification of segment unit due to overlooked copies in complex TR regions, leading to discrepancies in SV breakpoints between true alignments and observed SVs. **b)** Cross-alignment of Segment unit 1 and Segment unit 3 in both genomes after similarity calculation, hindering accurate breakpoint identification. **c)** Strategy employed to address TR regions for detecting INS or DEL.

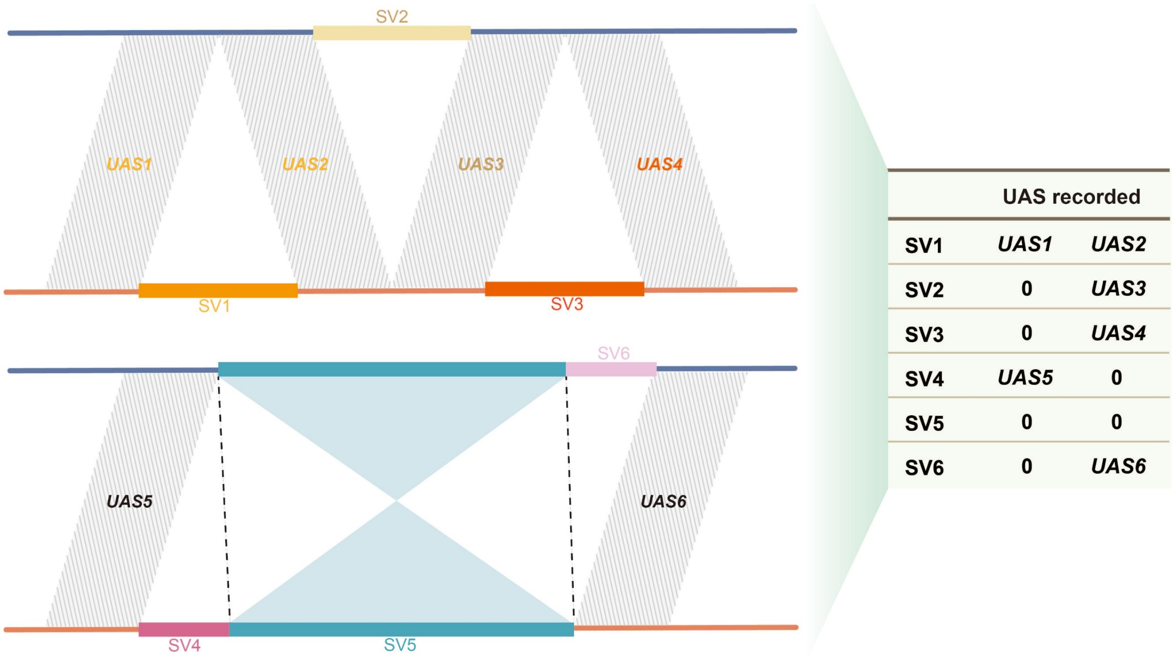

Supplementary Fig. 43 | Recording of UAS for multiple SVs within the same SV region. If UAS regions are shared between two SVs, only the first occurrence is recorded; subsequent occurrences are marked as “0”. Positions are also recorded as “0” if there is no UAS region between two consecutive SVs.

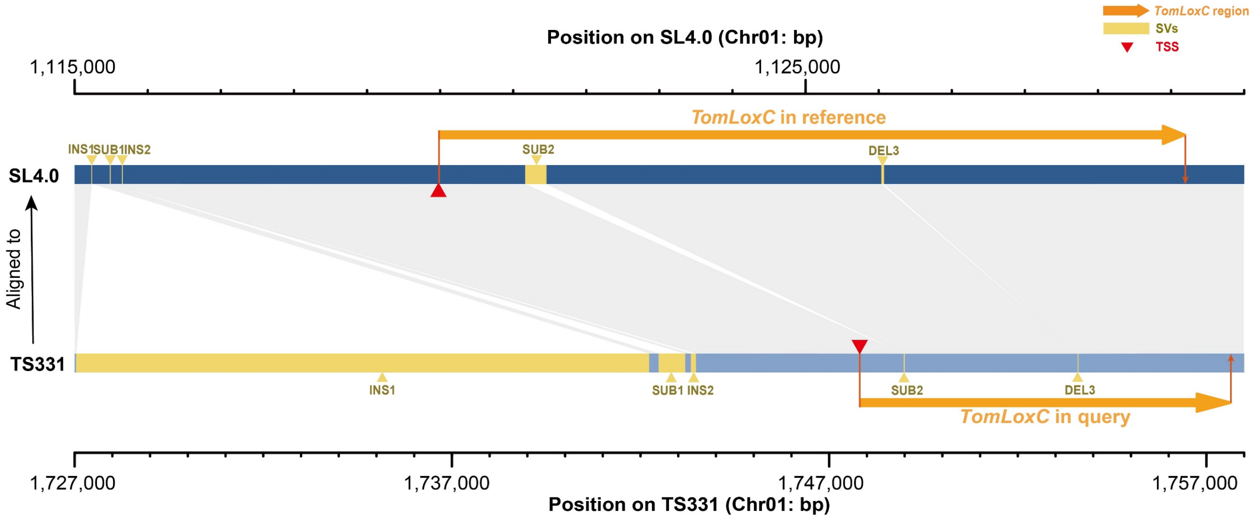

Supplementary Fig. 44 | Manual inspection of SVs in the promoter and gene region of *TomLoxC* in cultivated tomato TS331. The orange arrow indicates the *TomLoxC* region, the gray area indicates alignments, and light-yellow area indicates the SVs.

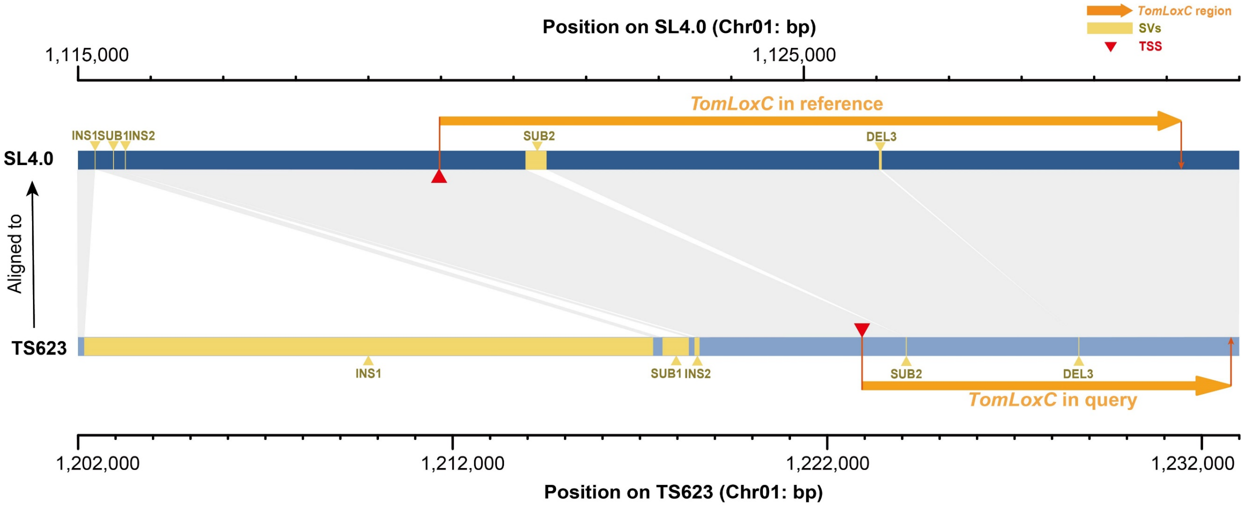

Supplementary Fig. 45 | Manual inspection of SVs in the promoter and gene region of *TomLoxC* in cherry tomato TS623.

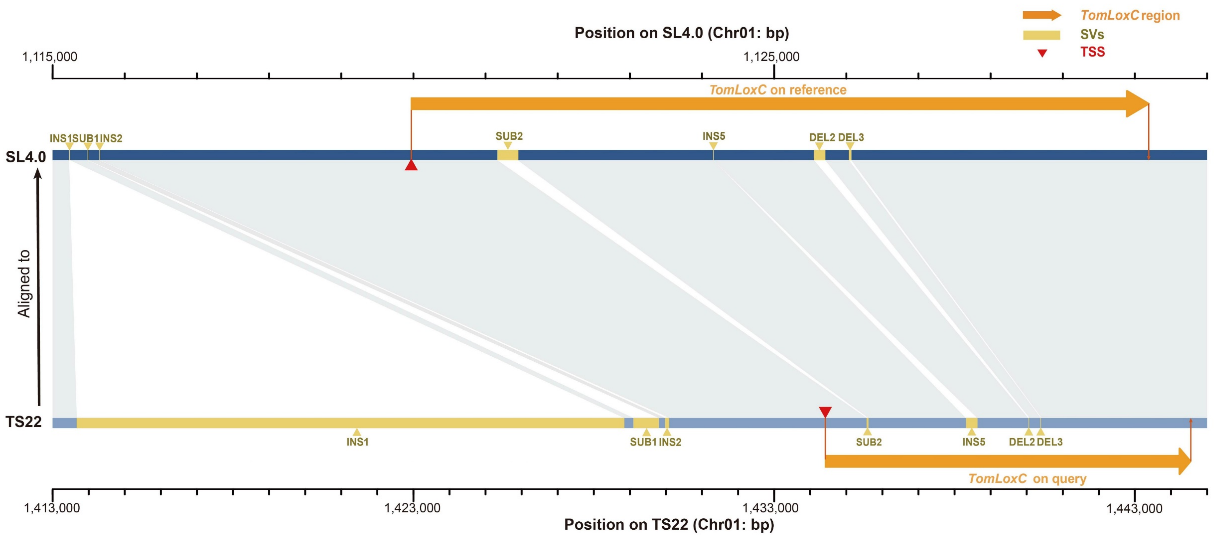

Supplementary Fig. 46 | Manual inspection of SVs in the promoter and gene region of *TomLoxC* in currant tomato TS22.

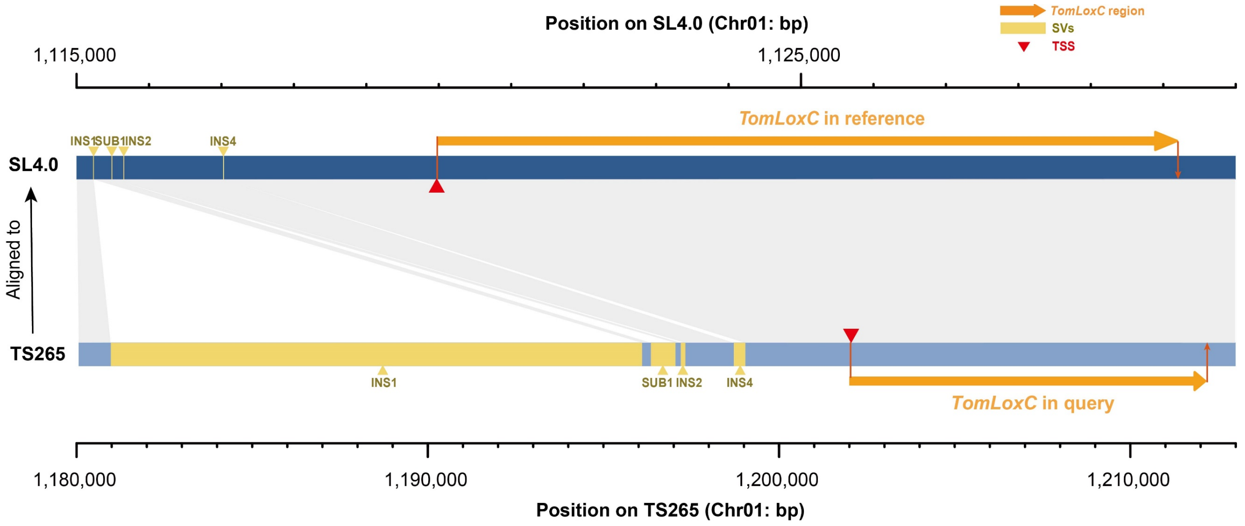

Supplementary Fig. 47 | Manual inspection of SVs in the promoter and gene region of *TomLoxC* in currant tomato TS265.

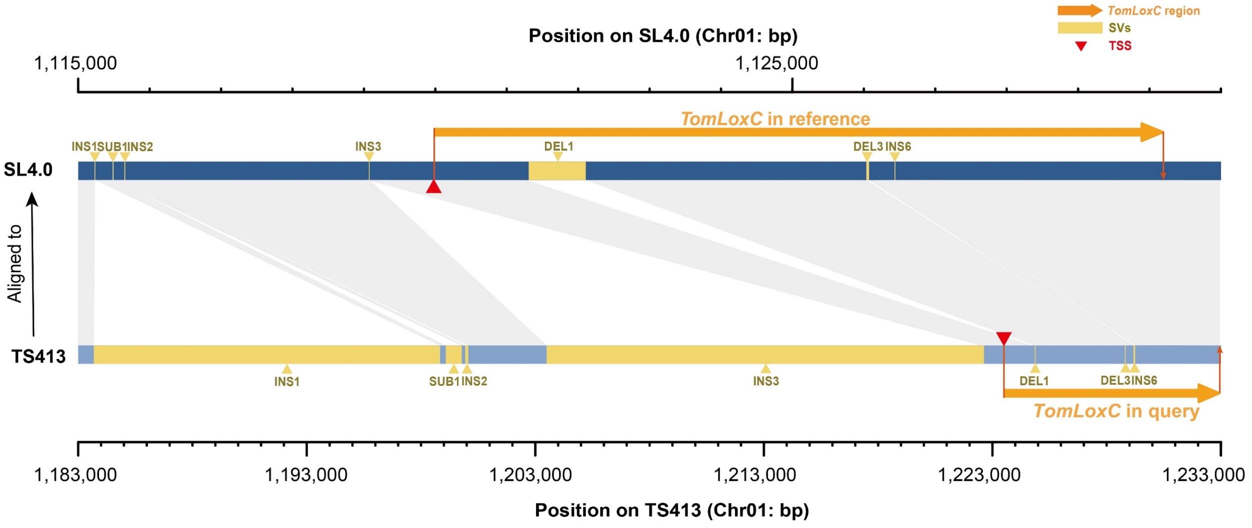

Supplementary Fig. 48 | Manual inspection of SVs in the promoter and gene region of *TomLoxC* in currant tomato TS413.

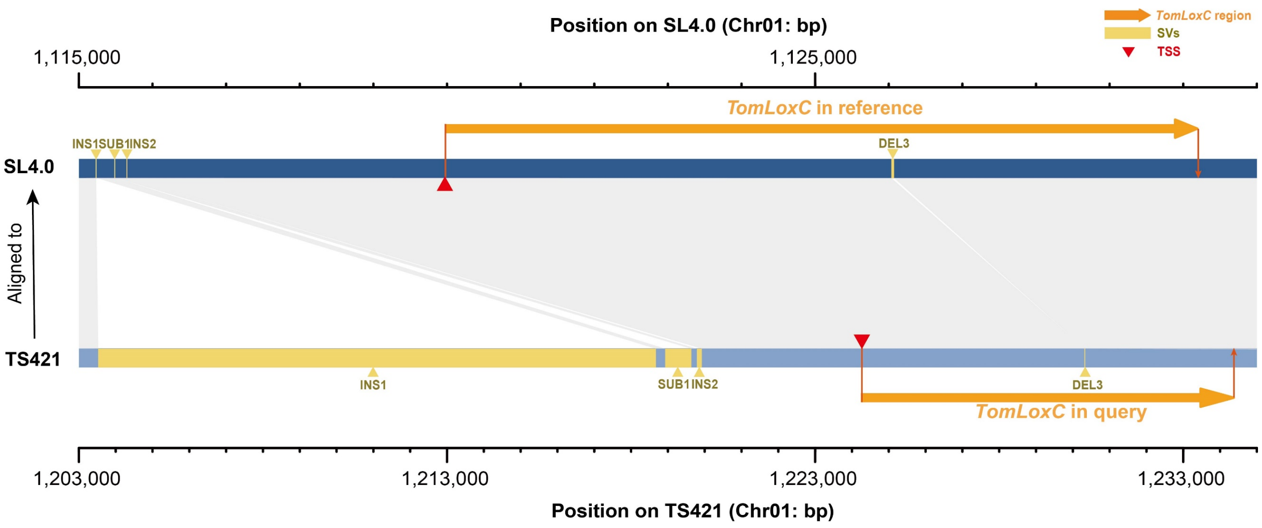

Supplementary Fig. 49 | Manual inspection of SVs in the promoter and gene region of *TomLoxC* in currant tomato TS421.

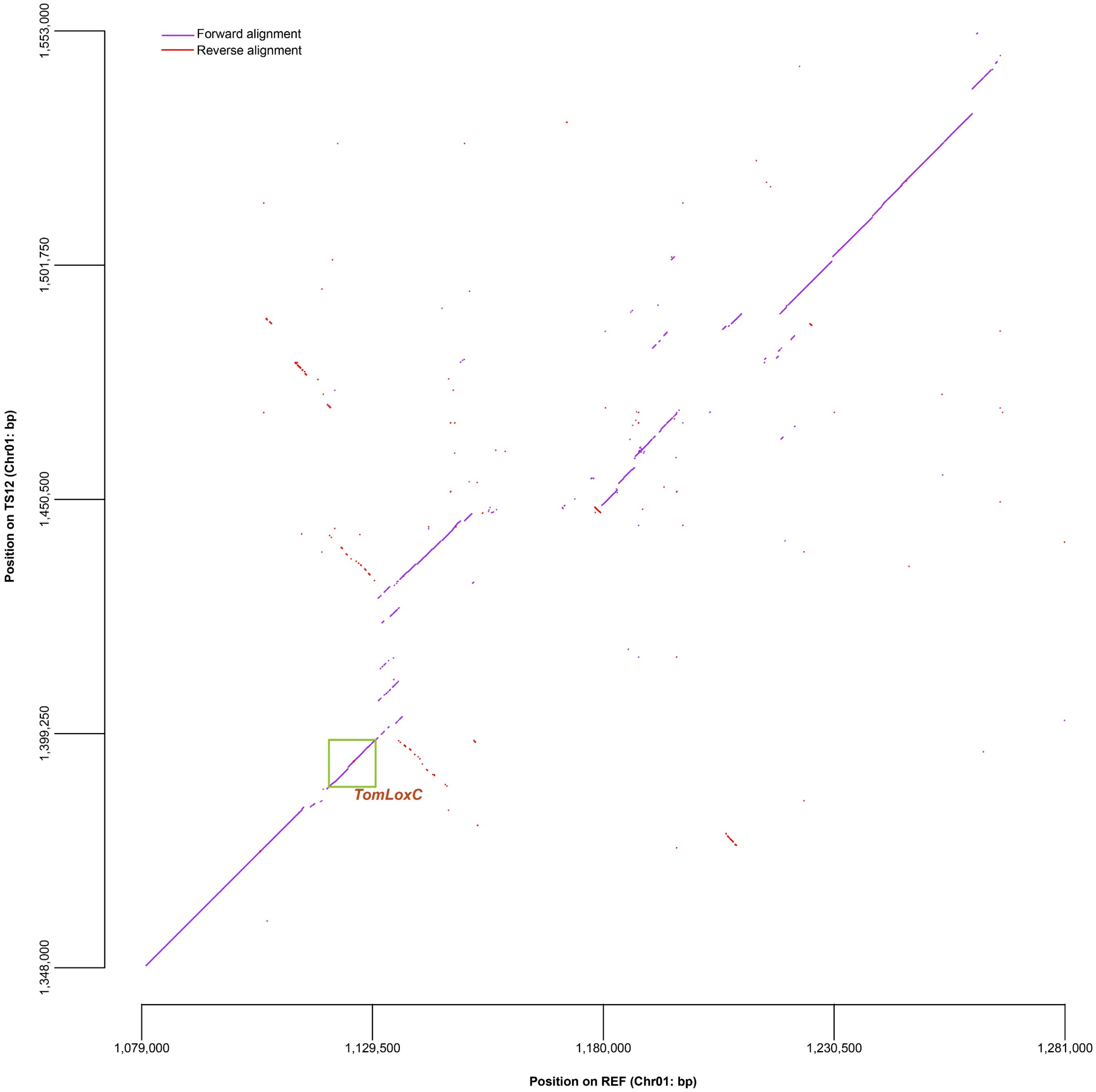

Supplementary Fig. 50 | Location of *TomLoxC* in cultivated tomato TS12.

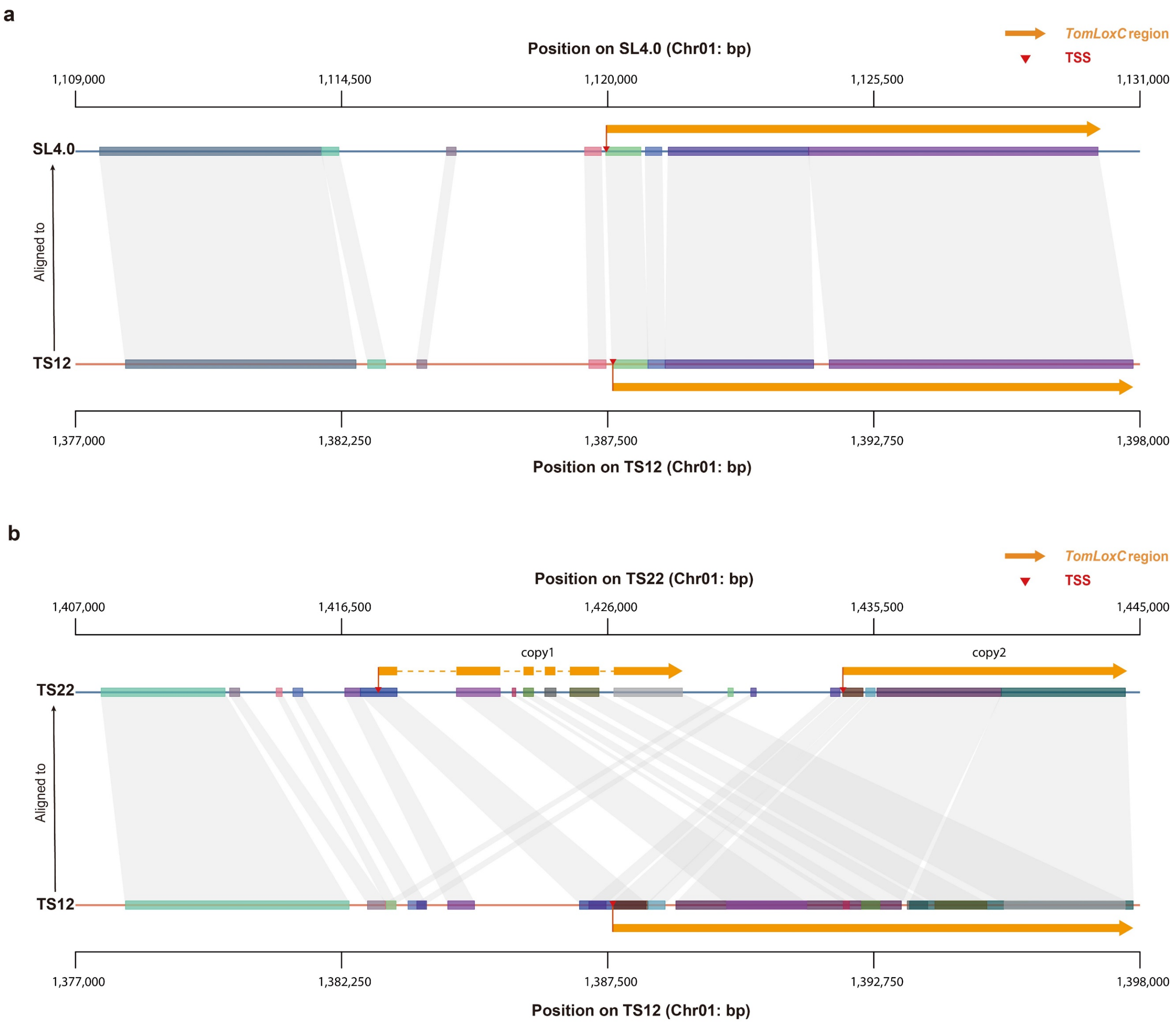

Supplementary Fig. 51 | Identification of SVs and copies of *TomLoxC* in TS12. a) Alignments between the cultivated tomato TS12 genome and the reference genome reveal two SUBs in the promoter region that do not align with the gene region, suggesting the loss of one copy of *TomLoxC* in TS12. **b)** Alignments between the cultivated tomato TS12 genome and the currant tomato TS22 genome indicates that the *TomLoxC* gene in TS12 is designated as copy2 due to fewer SVs compared to copy1 in the promoter region.

Reference

1. Cheng, H., Concepcion, G. T., Feng, X., Zhang, H. & Li, H. Haplotype-resolved *de novo* assembly using phased assembly graphs with hifiasm. *Nat. Methods* **18**, 170–175 (2021).
2. Kolmogorov, M., Yuan, J., Lin, Y. & Pevzner, P. A. Assembly of long, error-prone reads using repeat graphs. *Nat. Biotechnol.* **37**, 540–546 (2019).
3. Nurk, S. et al. HiCanu: accurate assembly of segmental duplications, satellites, and allelic variants from high-fidelity long reads. *Genome Res.* **30**, 1291–1305 (2020).
4. Zhang, J. et al. Genome puzzle master (GPM): an integrated pipeline for building and editing pseudomolecules from fragmented sequences. *Bioinformatics* **32**, 3058–3064 (2016).
5. Hosmani, P. S. et al. An improved de novo assembly and annotation of the tomato reference genome using single-molecule sequencing, Hi-C proximity ligation and optical maps. Preprint at *bioRxiv* https://doi.org/10.1101/767764 (2019).
6. Zhou, Y. et al. Graph pangenome captures missing heritability and empowers tomato breeding. *Nature* **606**, 527–534 (2022).
7. Li, K. P., Xu, P., Wang, J. P., Yi, X. & Jiao, Y. N. Identification of errors in draft genome assemblies at single-nucleotide resolution for quality assessment and improvement. *Nat. Commun.* **14**, 6556 (2023).
8. Camacho, C. et al. BLAST+: architecture and applications. *BMC Bioinformatics* **10**, 421 (2009).
9. Marçais, G. et al. MUMmer4: A fast and versatile genome alignment system. *PLoS Comput. Biol.* **14**, e1005944 (2018).
10. Thorvaldsdóttir, H., Robinson, J. T. & Mesirov, J. P. Integrative Genomics Viewer (IGV): high-performance genomics data visualization and exploration. *Brief. Bioinform.* **14**, 178–192 (2013).
11. Simão, F. A., Waterhouse, R. M., Ioannidis, P., Kriventseva, E. V. & Zdobnov, E. M. BUSCO: assessing genome assembly and annotation completeness with single-copy orthologs. *Bioinformatics* **31**, 3210–3212 (2015).

12. Rhie, A., Walenz, B. P., Koren, S. & Phillippy, A. M. Merqury: reference-free quality, completeness, and phasing assessment for genome assemblies. *Genome Biol.* **21**, 245 (2020).

13. Jiang, T. et al. Long-read-based human genomic structural variation detection with cuteSV. *Genome Biol.* **21**, 189 (2020).

14. Sedlazeck, F. J. et al. Accurate detection of complex structural variations using single-molecule sequencing. *Nat. Methods* **15**, 461–468 (2018).

15. Heller, D. & Vingron, M. SVIM: structural variant identification using mapped long reads. *Bioinformatics* **35**, 2907–2915 (2019).

16. Lin, J. et al. SVision: a deep learning approach to resolve complex structural variants. *Nat. Methods* **19**, 1230–1233 (2022).

17. Nattestad, M. & Schatz, M. C. Assemblytics: a web analytics tool for the detection of variants from an assembly. *Bioinformatics* **32**, 3021–3023 (2016).

18. Chakraborty, M., Emerson, J. J., Macdonald, S. J. & Long, A. D. Structural variants exhibit widespread allelic heterogeneity and shape variation in complex traits. *Nat. Commun.* **10**, 4872 (2019).

19. Song, B. et al. AnchorWave: sensitive alignment of genomes with high sequence diversity, extensive structural polymorphism, and whole-genome duplication. *Proc. Natl Acad. Sci. USA* **119**, e2113075119 (2022).

20. Goel, M., Sun, H., Jiao, W.-B. & Schneeberger, K. SyRI: finding genomic rearrangements and local sequence differences from whole-genome assemblies. *Genome Biol.* **20**, 277 (2019).

21. Li, H. Minimap2: pairwise alignment for nucleotide sequences. *Bioinformatics* **34**, 3094–3100 (2018).

22. Belyeu, J. R. et al. Samplot: a platform for structural variant visual validation and automated filtering. *Genome Biol.* **22**, 161 (2021).

23. Li, H. et al. The Sequence Alignment/Map format and SAMtools. *Bioinformatics* **25**, 2078–2079 (2009).

24. Quinlan, A. R. & Hall, I. M. BEDTools: a flexible suite of utilities for comparing genomic features. *Bioinformatics* **26**, 841–842 (2010).

25. Racine, J. gnuplot 4.0: a portable interactive plotting utility. *J. Appl. Econ.* **21**, 133-141 (2006).

26. Gao, L. et al. The tomato pan-genome uncovers new genes and a rare allele regulating fruit flavor. *Nat. Genet.* **51**, 1044–1051 (2019).
